# Multiple evolutionary pressures shape identical consonant avoidance in the world’s languages

**DOI:** 10.1073/pnas.2316677121

**Published:** 2024-06-25

**Authors:** Chundra A. Cathcart

**Affiliations:** ^a^Department of Comparative Language Science, University of Zurich, Zürich CH-8050, Switzerland; ^b^Center for the Interdisciplinary Study of Language Evolution, University of Zurich, Zürich CH-8050, Switzerland; ^c^Deutsche Forschungsgemeinschaft Center “Words, Bones, Genes, Tools”, University of Tübingen, Tübingen 72074, Germany

**Keywords:** linguistic evolution, sound patterns, phylogenetic modeling

## Abstract

Languages disfavor word forms containing sequences of similar or identical consonants, due to the biomechanical and cognitive difficulties posed by patterns of this sort. However, the specific evolutionary processes responsible for this phenomenon are not fully understood. Words containing sequences of identical consonants may be more likely to arise than those without; processes of word form mutation may be more likely to remove than create sequences of identical consonants in word forms; finally, words containing identical consonants may die out more frequently than those without. Phylogenetic analyses of the evolution of homologous word forms indicate that words with identical consonants arise less frequently than those without. However, words with identical consonants do not die out more frequently than those without. Further analyses reveal that forms with identical consonants are replaced in basic meaning functions more frequently than words without. Taken together, results suggest that the underrepresentation of sequences of identical consonants is overwhelmingly a by-product of constraints on word form coinage, though processes related to word usage also serve to ensure that such patterns are infrequent in more salient vocabulary items. These findings clarify aspects of processes of lexical evolution and competition that take place during language change, optimizing communicative systems.

## Significance

Languages are communicatively efficient systems, but little is known about the forces that optimize them. Phylogenetic modeling sheds light on the evolutionary mechanisms responsible for the low frequency of words containing sequences of identical consonants, a communicatively suboptimal sound pattern. Word forms without identical consonants are far more likely to be created than those with identical consonants. Mutational processes affecting word forms show a tendency to remove rather than create sequences of identical consonants, though not at greater-than-chance levels. Interestingly, however, word forms with identical consonants survive for as long as those without. Results indicate that the cross-linguistic underrepresentation of this sound pattern is overwhelmingly due to constraints on the production of variants, though multiple forces underlie this phenomenon.

The world’s spoken languages vary considerably in terms of the combinations of sounds they allow within words, as well as the frequencies of different static sound patterns they display. Preferences for specific combinations of sounds within words are highly stable within groups of closely related languages (1). At the same time, a number of quasi-universal patterns have been identified in large numbers of genetically diverse languages with respect to the sound patterns they display (2–4). One such phenomenon is the statistical underrepresentation of proximate similar or identical consonants within lexical items, documented in a diverse sample of languages: all else being equal, a sequence of identical consonants separated by a vowel is far less likely to be found in the vocabularies of the world’s languages than would be expected according to chance. In some language-specific cases, restrictions on such sequences are categorical: for instance, Arabic contains no words in which the first two consonants are identical (5, 6).

The avoidance of similar and identical adjacent consonants is reported in a variety of languages from different language families (7–11) and is connected with a more general avoidance of similar elements in human language (12). Experimental findings indicate that forms containing identical consonants are difficult to process and produce. Participants in lexical decision tasks are slower to recognize words and faster to reject nonwords containing identical consonants (13). Listeners are less likely to perceive ambiguous synthesized stimuli as containing identical consonants (14). Utterance onset times occur later for words containing similar sounds than for words containing dissimilar sounds in production experiments (15). Additionally, while repeated syllables are easier for children to produce and learn (16), adults exhibit a faster speech rate for sequences of different syllables (17), striking given that identical consonants are common in nursery words (e.g., mama, cookie, etc.).

Despite the well-documented nature of this phenomenon and ample experimental evidence that avoidance of this type is beneficial for both word production and comprehension, very little is known about the specific diachronic mechanisms involved in the emergence and maintenance of this pattern. There are several orthogonal processes of language change that may exert pressure on linguistic systems to disfavor word forms containing identical consonants, but the role of these different mechanisms remains unexplored. One possibility is that words containing identical consonants arise in languages with low frequency, seldom coined or borrowed from other speech varieties; if an individual innovates such a form, the chances of its conventionalization within a speech community and survival for an interval of time are low. Given the large body of psycholinguistic evidence that words containing identical consonants are more difficult to produce and process than those without, it may be the case that they are less likely to enter a language’s vocabulary and that when a word form arises on a phylogenetic lineage, it is unlikely to contain a sequence of identical consonants.

A second possibility is that mutational processes such as sound changes, analogical changes, and other developments operating upon word forms frequently remove sequences of identical consonants when present, and rarely introduce them when they are absent. Mutational processes sometimes give rise to sequences of identical consonants within words; for instance, a regular sound change involving consonant cluster simplification gave rise to Sundanese dedek “rice bran,” which descends from earlier Proto-Malayo-Polynesian *dekdek. The first consonant of the form directly ancestral to Latin bibet “he/she drinks” was most likely *p-* (cf. Sanskrit pibati, with the same meaning), but changed to *b-*, likely on analogy with reduplicated verbs for which the consonant of the reduplicant matches the consonant of the base, before reduplication in verbs ceased to be a productive process. Similar morphological mutations can also be responsible for the blurring of boundaries between transparent subword units (morphemes), creating tautomorphemic (within-morpheme) sequences of identical consonants from heteromorphemic (across-morpheme) ones [e.g., Aklanon babáyi “woman” is descended from a complex reduplicated form *ba-bahi but is analyzed synchronically as a single morpheme (18)]; sequences of identical consonants are tolerated to a lesser degree within morpheme boundaries than across them (19, 20). At the same time, mutations are also capable of removing sequences of identical consonants within word forms. While developments specifically dedicated to removing sequences of identical consonants are infrequent in surveys of sound changes (8, 21), more general sound changes may be responsible for the relative rarity of such sequences (e.g., Latin bibere drink developed to Old French beivre due to a general weakening of word-medial *-b-* to *-v-* that affected other forms, not just those containing two instances of *b*).

A third view found in the literature but untested empirically on a large scale hypothesizes that words containing sequences of identical consonants are rare due to dynamics of lexical replacement. While a number of processes may lead to the presence of identical consonants in a word, a lexical item may be phased out of use relatively rapidly once such a pattern arises in it, losing ground to competitor forms that do not contain the same disfavored sound pattern (7, 8, 22). This view of lexical change invites clear analogies with biological notions of selectional pressure, a force invoked in previous work on lexical evolution (23): while a number of processes may give rise to variation in forms corresponding to a given meaning, language users will select against forms that are deleterious from the perspective of language production and processing.

**Fig. 1.**
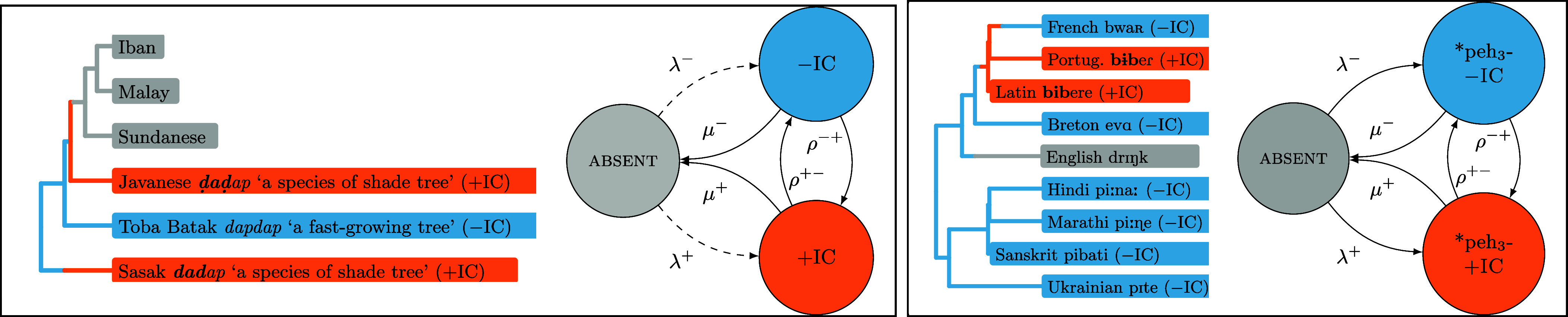
Schemata of continuous-time Markov models of evolution for a cognate class trait representing the Proto-Malayo-Polynesian etymon *dapdap (*Left*) and a cognate-concept trait representing whether languages use the Proto-Indo-European root *peh_3_- in the meaning “drink” (*Right*). Both trait types undergo transitions between states representing absence, presence without identical consonants, and presence with identical consonants. Tree branch colors represent hypothetical but unobserved character histories (i.e., evolutionary trajectories) involving transitions between states (color changes on branches). Transition rates (representing frequencies of transitions between states) can be inferred on the basis of 1) data attested in languages and 2) language phylogenies. Parameters governing the evolution of the traits given here can be subdivided into birth rates of traits without ($\lambda_{0}^{-}$) and with ($\lambda_{0}^{+}$) identical consonants (transitions from absent to ±IC), rates involving mutations introducing ($\rho_{0}^{-+}$) or removing ($\rho_{0}^{+-}$) sequences of identical consonants (transitions between ±IC), and loss rates involving the death of cognate classes or concept-cognate traits with ($\mu_{0}^{+}$) and without ($\mu_{0}^{-}$) sequences of identical consonants (transitions from ±IC to absent). The dashed lines in the schema in the lefthand panel represent the understanding that cognate classes are born only once.

Finally, more than one of the three pressures identified above may be involved in persistence of identical consonant avoidance. Phylogenetic comparative methods (Fig. 1) were used to model the evolutionary dynamics of cognate class (i.e., homologous, etymologically related words that share a common origin but may differ in meaning) as well as cognate-concept traits (i.e., features which register whether a language uses a cognate class in a particular meaning function, alternatively referred to as root-meaning traits) in a diverse sample of the world’s language families. Analyzing both of these data types sheds light on complementary dynamics of lexical evolution. On one hand, cognate classes provide a picture of the full evolutionary trajectory of homologous formal elements across related languages, but do not provide explicit information regarding lexical competition and replacement: a word form may die out conceivably because it loses out to a competitor, but as it is challenging to pinpoint the specific semantic function in which the word served before dying out, we have no information about the form that came to replace it. Cognate-concept traits provide an explicit way to model lexical competition and replacement in that we can explicitly track the forms that replace each other in particular meaning functions; at the same time, models based on these features do not allow us to make inferences regarding the trajectory of a form before it comes to serve in a meaning function or after it is replaced: a form may be replaced in a specific meaning function but may go on to express another concept rather than die out. The two sets of analyses conducted are designed to disentangle the role of the three mechanisms outlined above. Key results are summarized in Table 1.

**Table 1.** Interpretation of parameters used in analyses of cognate class and cognate-concept traits, along with the research questions they are used to address as well as answers

Rate 1	Rate 2	Question addressed
Cognate class traits		
$\lambda_{0}^{-}$ (birth rate, −IC)	$\lambda_{0}^{+}$ (birth rate, +IC)	Do word forms without IC arise more frequently than forms with IC? (Yes, more frequently than at chance)
$\rho_{0}^{+-}$ (mut. rate, +IC→−IC)	$\rho_{0}^{-+}$ (mut. rate, −IC→+IC)	Are +IC→−IC changes more frequent than −IC→+IC changes? (Yes, but not more frequently than at chance)
$\mu_{0}^{+}$ (loss rate, +IC)	$\mu_{0}^{-}$ (loss rate, −IC)	Are forms with +IC more likely to die out than forms with −IC? (No)
Cognate-concept traits		
$\lambda_{0}^{-}$ (birth rate, −IC)	$\lambda_{0}^{+}$ (birth rate, +IC)	Do word forms without IC enter the basic vocabulary more frequently than forms with IC? (Yes, but not more frequently than chance in 4/5 families)
$\rho_{0}^{+-}$ (mut. rate, +IC→−IC)	$\rho_{0}^{-+}$ (mut. rate, −IC→+IC)	Are +IC→−IC changes more frequent than −IC→+IC changes in basic vocabulary items? (More frequent than chance in only 1/5 families)
$\mu_{0}^{+}$ (loss rate, +IC)	$\mu_{0}^{-}$ (loss rate, −IC)	Are forms with +IC phased out of basic meaning functions more often than forms with −IC? (Yes)

Subscript zeros indicate that parameters represent log mean rates around which rates for individual traits are log-normally distributed (with the exception of $\lambda_{0}^{\pm }$ for cognate class traits; see text). Each hypothesis is assessed by computing the ratio between rates 1 and 2 after exponentiating them.

## Cognate Class Traits

Bayesian phylogenetic models were used to disentangle the mechanisms that shape the evolutionary trajectories of individual cognate classes (e.g., forms descending from Proto-Malayo-Polynesian *dapdap) in three families (Austronesian, Semitic, and Uralic). Over the course of a language family’s phylogenetic history, ancestral word forms are born, undergo processes of word form mutation and differentiation (as the speech varieties in which they exist diversify phylogenetically), and die out on different phylogenetic lineages. Analyses of the evolution of morpheme-internal identical consonants within cognate class traits in three language families were carried out using a hierarchical phylogenetic model containing six parameters of interest (schematized in Fig. 1): $\lambda_{0}^{-}$, the log birth rate of forms without identical consonants; $\lambda_{0}^{+}$, the log birth rate of forms with identical consonants; $\rho_{0}^{-+}$, the log mean rate at which sequences of identical consonants arise within forms; $\rho_{0}^{+-}$, the log mean rate at which sequences of identical consonants are lost within forms; $\mu_{0}^{-}$, the log mean loss rate of forms without identical consonants; and $\mu_{0}^{+}$, the log mean loss rate of forms with identical consonants. The hierarchical model used allows parameters to vary at the level of individual cognate classes, which undergo change according to evolutionary rates that are log-normally distributed around the mean parameters $\rho_{0}^{-+}$, $\rho_{0}^{+-}$, $\mu_{0}^{-}$, $\mu_{0}^{+}$, or in the case of birth rates, according to which all cognate classes arise and which are shared across all cognate classes, set to $\exp\left(\lambda_{0}^{-}\right)$ and $\exp\left(\lambda_{0}^{+}\right)$. Parameters that vary at the level of individual cognate classes are analogous to random effects in mixed-effects regression models, in that they account for individual cognate-level idiosyncrasies, while the mean parameters listed above are comparable to fixed effects, as they capture global trends in the evolutionary system. Pairwise comparisons between parameters allow us to assess whether forms with and without identical consonants are born at different rates ($\lambda_{0}^{+}$ vs. $\lambda_{0}^{-}$), whether identical consonants are gained and lost within forms at different rates ($\rho_{0}^{-+}$ vs. $\rho_{0}^{+-}$), and whether forms with and without identical consonants are lost at different rates ($\mu_{0}^{+}$ vs. $\mu_{0}^{-}$). Strengths of differences in rates were quantified by taking the ratio of the two mean rates in question, i.e., by inspecting the posterior distributions of the quantities $\exp\left(\lambda_{0}^{-}-\lambda_{0}^{+}\right)$, $\exp\left(\rho_{0}^{+-}-\rho_{0}^{-+}\right)$, and $\exp\left(\mu_{0}^{+}-\mu_{0}^{-}\right)$. Evidence for a difference is taken to be decisive if the 95% highest-density interval of ratios does not contain values representing the null hypothesis (24). A standard null value is 1: ratios greater than 1 indicate that one change type is more frequent than another. However, in some cases, skewed distributions are expected even under null models of language generation (25). For this reason, posterior ratios are also compared to quantities representing baseline asymmetries in frequencies of change types that would be expected under neutral processes of language evolution.

**Fig. 2.**
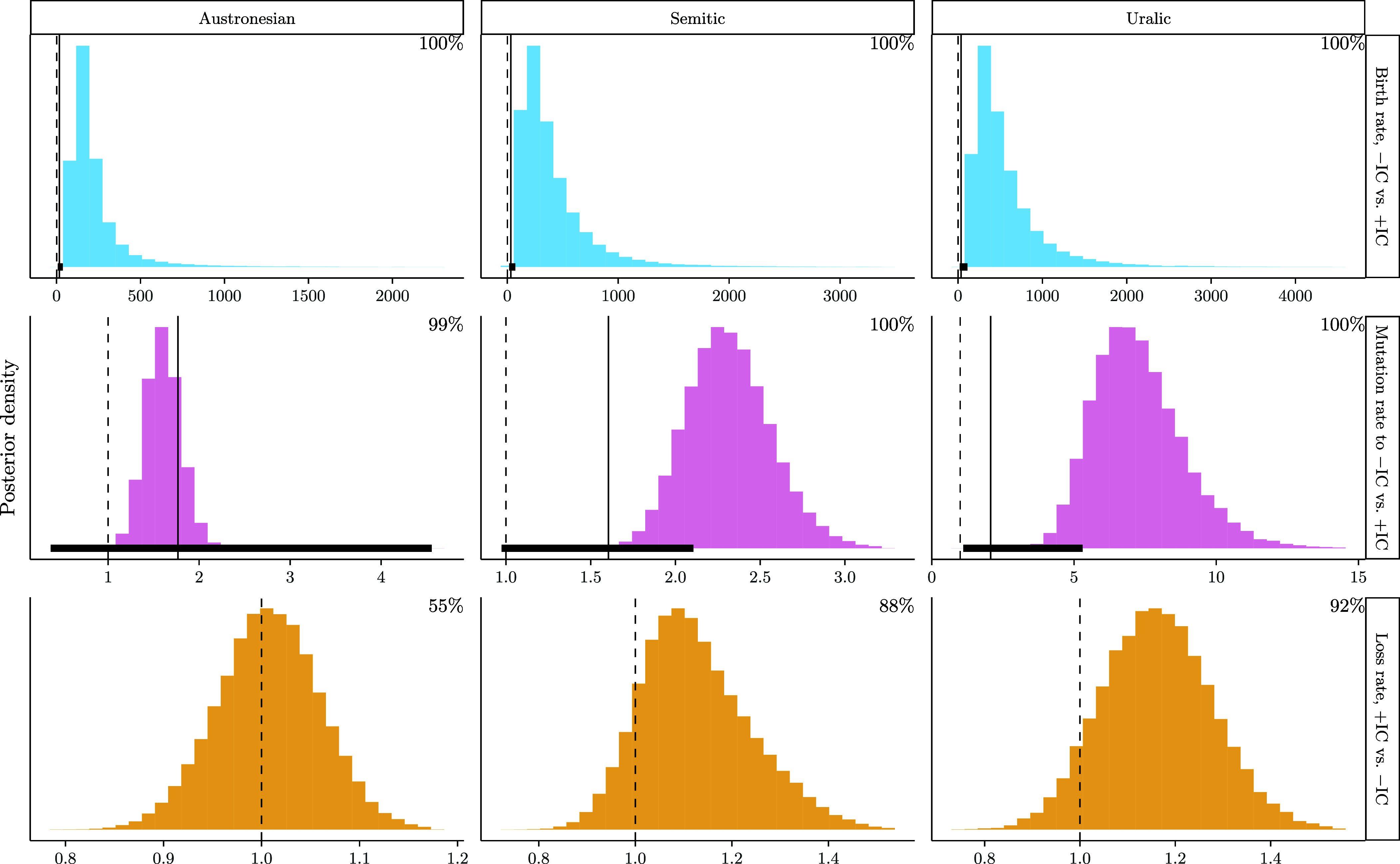
Histograms from analyses of cognate traits displaying posterior distributions of ratios of parameters of interest for different families: birth rate of words with value −IC (no identical consonants) vs. +IC (with identical consonants; *Top*), rate of +IC →−IC vs. −IC →+IC change (*Middle*), and loss rate of words with +IC vs. −IC (*Bottom*). Histograms are annotated with percentages of samples for which ratios are greater than 1 (given by vertical dashed lines). Solid black vertical lines in upper two rows represent median baseline quantities; horizontal lines represent ranges of baseline quantities.

Fig. 2 shows posterior distributions of ratios of interest. Distributions are annotated with the percentage of posterior samples for which the ratio is greater than one (represented by dashed lines). Distributions of ratios pertaining to birth rates and mutation rates are also annotated with values representing ranges of ratios (and median values thereof) that would be expected under neutral models of language change. These quantities are estimated from data from each family under analysis, assuming that distributions of features found in contemporary languages are representative of those encountered during the history of the language family to which they belong (26). Under a neutral process in which words are generated by randomly sampling segments with uniform probabilities, the ratio of words born without versus with sequences of identical consonants is no greater than the number of consonants in a language’s segmental inventory, minus one (*Materials and Methods*). This quantity is provided for languages in each family for which such data are available. Baseline values for ratios between mutations that remove versus introduce sequences of identical consonants are estimated by simulating the effects of neutral models of sound change (27, 28) using word lists of languages in the families under study. For loss rates, a baseline value of 1 is sufficient for the purpose of interpreting posterior ratios.

Across all three families, there is decisive evidence that forms without identical consonants are born more frequently than those with identical consonants (median: 171.49, 95% HDI: [51.67, 526.09]; 303.07, [53.17, 1017.29]; and 428.73, [89.93, 1365.98] times more frequently in Austronesian, Semitic, and Uralic, respectively). Additionally, there is decisive evidence that these ratios are greater than would be expected under a chance baseline based on sizes of segmental inventories, as posterior HDIs are consistently greater in value than ranges of baselines expected under a neutral process of word generation (median: 17, total range: [8, 37]; 32, [16, 73]; and 35, [21, 112] times more frequently in Austronesian, Semitic, and Uralic, respectively). Mutational changes to forms that remove sequences of identical consonants are decisively more frequent than mutations that introduce them, although the ratios between transition rates pertaining to these changes are far lower than asymmetries in birth rates of forms with and without identical consonants (1.6, [1.23, 1.99]; 2.30, [1.85, 2.82]; and 7.09, [4.44, 10.45] in Austronesian, Semitic, and Uralic, respectively). Of note, 95% HDIs overlap with ranges of ratios expected under neutral models of sound change in all three families (1.76, [0.36, 4.55]; 1.60, [0.97, 2.10]; and 2.06, [1.11, 5.30] more frequently in Austronesian, Semitic, and Uralic, respectively), indicating that these ratios do not unambiguously exceed what is expected at chance levels. Posterior distributions do not support the idea that forms with sequences of identical consonants die out more frequently than those without them (1.01, [0.9, 1.11]; 1.11, [0.92, 1.37]; and 1.16, [0.94, 1.39] in Austronesian, Semitic, and Uralic, respectively.

These results indicate that asymmetries in birth rates of words play a major and consistent role in the underrepresentation of sequences of identical consonants in word forms, and to a weaker extent processes that mutate word forms, though this latter effect is not found in all families studied when interpreted according to a principled, conservative baseline. Crucially, however, word forms containing such sequences are no more likely to fall entirely out of use than those without: they exhibit as much longevity as their counterparts that do not contain identical consonants, though it is not clear from these results whether they survive in more marginal functions and restricted distributions.

## Cognate-Concept Traits

A related set of phylogenetic models were used to analyze the evolution of morpheme-internal sequences of identical consonants within cognate-concept traits in five language families (Dravidian, Indo-European, Sino-Tibetan, Turkic, and Uto-Aztecan). These analyses shed light on the conditions under which cognate word forms enter and fall out of use in basic meaning functions, and the nature of the processes affecting word forms during the time in which they occupy such roles. Analyses focused on cognate-concept traits pertaining to one hundred concepts representing basic vocabulary items, chosen to maximize comparability of results across families (29). Parameters of interest have interpretations analogous to those in the models described in the previous section (Fig. 1). As above, posterior parameter values were compared to assess whether word forms without identical consonants enter basic vocabulary meaning functions more frequently than those with them ($\lambda_{0}^{-}$ vs. $\lambda_{0}^{+}$), whether identical consonants are lost within forms used in the basic vocabulary more frequently than they are gained ($\rho_{0}^{+-}$ vs. $\rho_{0}^{-+}$), and whether forms containing identical consonants are removed from the basic vocabulary more frequently than those without ($\mu_{0}^{+}$ vs. $\mu_{0}^{-}$). The baselines against which ratios for birth and mutation rates are compared differ from those employed for cognate class traits. Ratios of birth rates (i.e., between the rates at which forms without and with identical consonants enter languages’ basic vocabulary) are compared to ratios between frequencies of forms without versus containing identical consonants in contemporary languages’ general vocabularies (comprising basic and nonbasic items pooled together); this comparison tells us whether forms with identical consonants enter the basic vocabulary at a rate lower than would be expected from a neutral process in which basic vocabulary items are sampled randomly from the lexicon of a language. Ratios between mutation rates are compared to baselines generated via simulations of neutral sound change, as for cognate class traits, but restricted to forms expressing the one hundred concepts under analysis. As with cognate class traits, ratios between rates at which forms with and without identical consonants are removed from the basic vocabulary do not require interpretation against a baseline other than the standard null value of 1.

**Fig. 3.**
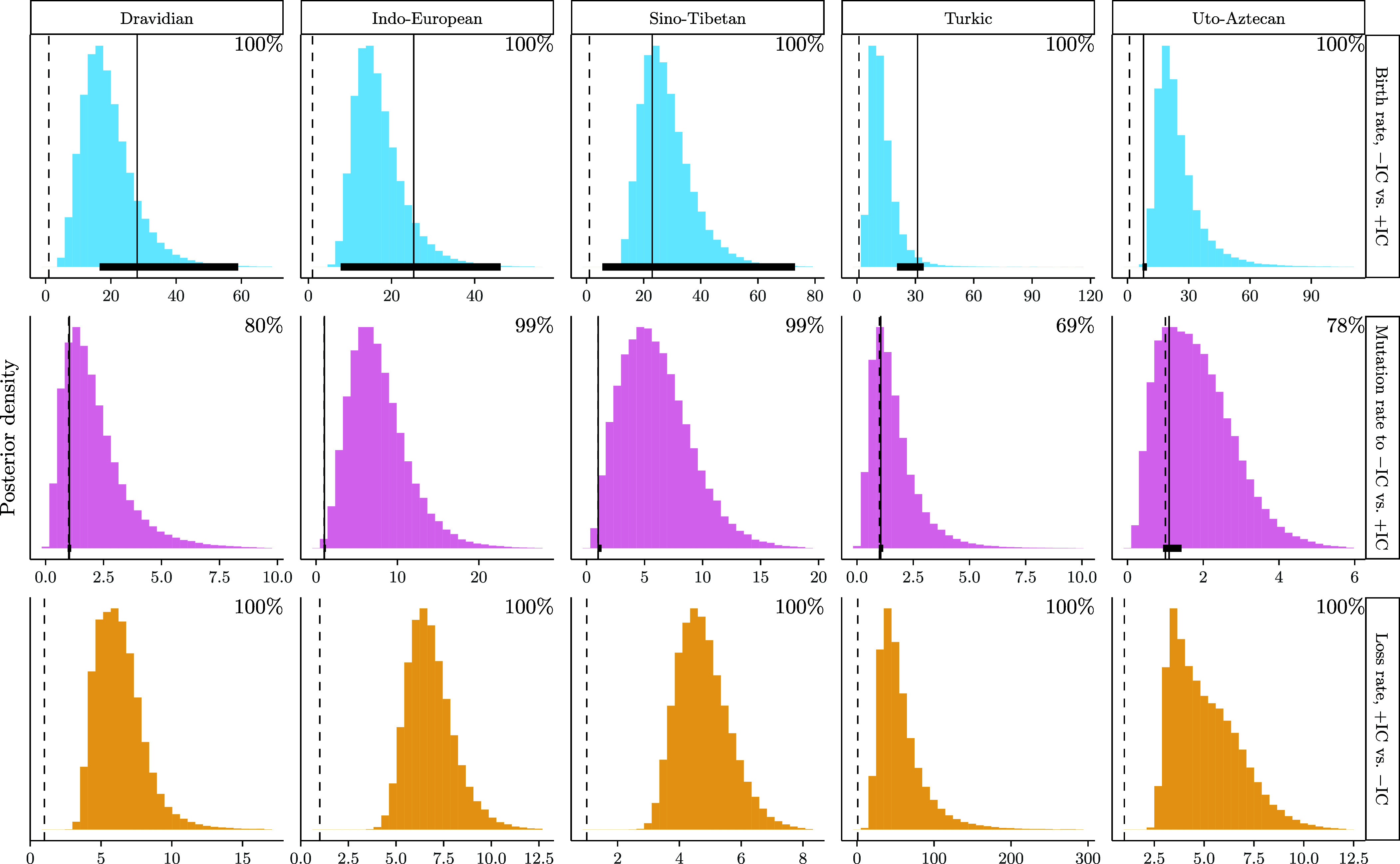
Histograms from analyses of cognate-concept traits displaying posterior distributions of ratios of parameters of interest for different families: birth rates of cognate-concept traits with −IC vs. +IC (*Top*), rates of +IC →−IC vs. −IC →+IC change (*Middle*) within cognate-concept traits, and loss rates of cognate-concept traits with +IC vs. −IC (*Bottom*). Histograms are annotated with percentages of samples for which ratios are greater than 1 (given by vertical dashed lines). Solid black vertical lines in upper two rows represent median baseline quantities; horizontal lines represent ranges of baseline quantities.

Fig. 3 shows posterior distributions of ratios of interest. Distributions are annotated as in Fig. 2. All families show decisive evidence that forms without identical consonants enter the basic vocabulary more frequently than forms with identical consonants (Dravidian: 17.85, [6.03, 35.98]; Indo-European: 15.85, [7.36, 29.69]; Sino-Tibetan: 26.56, [13.33, 46.81]; Turkic: 11.59, [3.41, 27.09]; Uto-Aztecan: 21.95, [9.47, 47.15]); however, these distributions overlap with ranges of ratios expected under a random sampling process from the general vocabulary in all families (Dravidian: 28, [16.5, 59]; Indo-European: 25.3, [7.78, 46.2]; Sino-Tibetan: 23.0, [5.56, 73]; Turkic: 31.1, [20.6, 34.2]; Uto-Aztecan: 7.89, [7.42, 9.46]) except for Uto-Aztecan, where usable digitized word lists comprising basic and nonbasic vocabulary items were available for only three languages.

Indo-European is the only family exhibiting decisive evidence that mutational processes remove sequences of identical consonants from basic vocabulary items more frequently than they introduce them (Dravidian: 1.77, [0.18, 4.81]; Indo-European: 7.14, [1.55, 15]; Sino-Tibetan: 5.66, [0.95, 11.81]; Turkic: 1.38, [0.17, 3.76]; Uto-Aztecan: 1.7, [0.25, 3.67]); for Sino-Tibetan, the less conservative 89% HDI ([1.24, 10.18]) does not overlap with one. Additionally, Indo-European posterior ratios do not overlap with ranges that would be expected under neutral processes of sound change affecting the basic vocabulary (Dravidian: 1.02, [0.94, 1.10]; Indo-European: 1.01, [0.92, 1.19]; Sino-Tibetan: 1.00, [0.97, 1.28]; Turkic: 1.05, [0.97, 1.16]; Uto-Aztecan: 1.09, [0.94, 1.42]), but there is considerable overlap for other families. It is not immediately clear why Indo-European is an outlier; the Indo-European family exhibits well-documented dissimilatory sound changes that occur in parallel in multiple phylogenetic lineages (30), but such changes are phonetically natural and there is no reason not to expect them to a similar degree in other families.

All families show decisive support for the idea that cognate-concept traits are lost more frequently when the form expressing the concept in question contains identical consonants than when it does not (Dravidian: 6.11, [3.59, 9.59]; Indo-European: 6.67, [4.65, 9.36]; Sino-Tibetan: 4.68, [3.27, 6.44]; Turkic: 49, [16.56, 118.88]; Uto-Aztecan: 4.77, [2.65, 8.34]). This indicates that while word forms with identical consonants do not exhibit less overall longevity than word forms without identical consonants, they are phased out of basic meaning functions more frequently than those without.

The rates reported above characterize the dynamics of lexical replacement within the basic vocabulary as a whole. Variation among rates was inspected at the concept level in order to investigate the extent to which relative strengths of asymmetries between rates vary across concepts. Pairwise comparisons of asymmetry strength between concepts were carried out by computing the percentage of samples for which a ratio between rates (birth, mutation, and loss) was greater in one concept than another, with evidence for a contrast taken to be decisive for percentages of 95% or more (31). Hardly any comparisons involving ratios between birth rates exhibit decisive evidence for a difference (Dravidian: 0 out of 4,278 pairwise comparisons; Indo-European: 0/4,560; Sino-Tibetan: 1/3,403; Turkic: 83/4,005; Uto-Aztecan: 0/4,186), along with mutation rates (Dravidian: 0/4,278; Indo-European: 30/4,560; Sino-Tibetan: 48/3,403; Turkic: 2/4,005; Uto-Aztecan: 6/4,186). On the other hand, ratios between loss rates (+IC vs. −IC) exhibit a higher number of decisive contrasts (Dravidian: 1,469/4,278; Indo-European: 2,122/4,560; Sino-Tibetan: 2,038/3,403; Turkic: 666/4,005; Uto-Aztecan: 2,132/4,186), indicating that while on the whole, forms with sequences of identical consonants are phased out of the basic vocabulary at a higher rate than forms without, the strength of this tendency differs considerably across concepts.

**Fig. 4.**
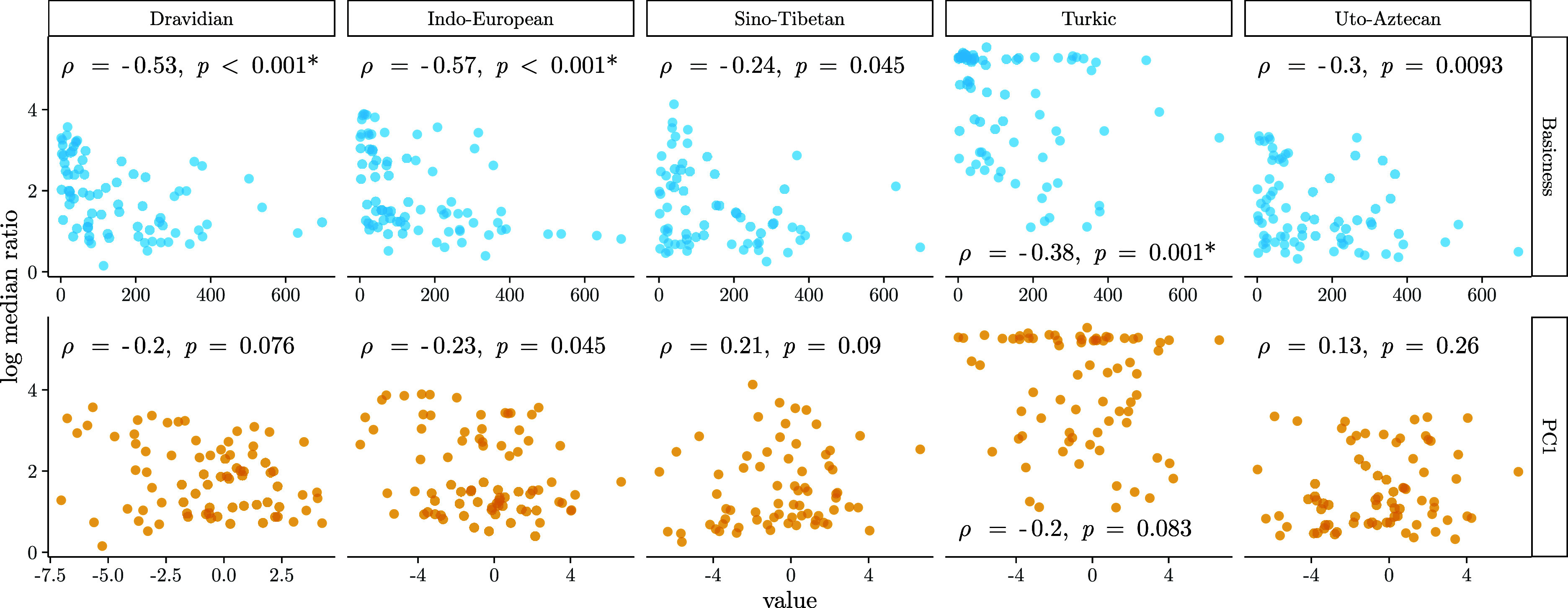
Scatter plots annotated with correlations (Spearman’s *ρ*) between median ratios (log-transformed for better visibility) between cognate-concept loss rates with vs. without IC (*y* axis) and basicness (*x* axis, top row) and the first principal component of cross-linguistic concept frequencies (*x* axis, bottom row), for all five families analyzed in this paper. Negative correlations between log median rates and each metric indicate that the tendency to replace forms with identical consonants (relative to those without identical consonants) is weaker in less basic/frequent concepts than in more basic/frequent ones. *P*-values for correlations that are significant after correcting for multiple testing (at α=0.005=0.05/10) are marked with an asterisk (*).

Fig. 4 shows correlations between median loss rate asymmetries (representing the frequency with which a word containing identical consonants in replaced in a meaning function relative to a word without them) and two metrics related to the need probabilities (32–34) of concepts in usage. The first of these metrics represents the basicness of a concept (35) and is based on word form information content and diachronic stability (lower values indicate more basic concepts). The second metric is the first principle component (PC1) derived from a cross-linguistic dataset of basic concept frequencies (36), with lower values indicating more frequent concepts. Both metrics are positively correlated with each other (Spearman’s ρ=0.47, P<0.001) and negatively correlated with the notion of concept-level need probability. Loss rate asymmetries are negatively correlated with both metrics for the majority of families, lending support to the idea that the tendency to replace forms containing identical consonants (relative to those without identical consonants) is weaker in concepts with lower need probability than in those with higher need probability. However, correlations between loss rate asymmetries and basicness are significant for only three out of five families after correcting for multiple testing, and correlations between loss rate asymmetries and PC1 of concept frequencies are not significant for any family after the same correction. These results present mixed support for the idea that identical consonants are more tolerated in less basic, frequent concepts. It is not immediately clear why basicness correlates more strongly with loss rate asymmetries than a metric derived from frequencies; both metrics are aggregate measures incapable of capturing certain finer-grained dynamics of usage, so the discrepancy cannot be explained by this attribute alone. Regardless of how these relationships are interpreted, the fact remains that there is the greatest degree of interconcept variation in terms of loss rate asymmetries; therefore, it appears to be the case that vocabulary replacement is the component of change most capable of allowing forms containing identical consonants to remain in some cognate-concept traits but not others.

In sum, analyses of the evolution of concept-cognate traits show that while forms without sequences of identical consonants enter the basic vocabulary more frequently than forms containing such sequences, these ratios are comparable (in all but one family) to what would be expected from sampling at random from the lexicons of contemporary languages in each family. There is not clear evidence across families that mutation rates are more likely to remove sequences of identical consonants than introduce them. Loss rates for cognate-concept traits consistently display rate asymmetries in favor of forms without identical consonants. At the same time, for loss rates, there is some (albeit limited) evidence that the strength of these asymmetries is weaker for concepts with lower need probability. Extrapolating these dynamics beyond the basic vocabulary, forms with identical consonants can be expected to survive in concept slots that are infrequently used and marginal for roughly as long as forms without.

## Discussion

Various aspects of linguistic systems, including lexical items, are argued to be optimized for communicative efficiency (37, 38). This study introduces an explicit model designed to disentangle orthogonal mechanisms that work to make linguistic systems communicatively optimal. These include forces that govern births and losses of individual word forms, as well as pressures that introduce forms into and remove them from salient meaning roles; additionally, the model employed sheds light on processes that mutate word forms over the course of their lifetimes as well as when they occupy salient meaning roles. Results indicate that different evolutionary mechanisms impact on the introduction and maintenance of efficient patterns to different degrees.

Word forms containing sequences of identical consonants, a characteristic shown to pose problems for word production and comprehension, arise far less frequently than those without, and far less frequently than would be expected under a neutral process of word form generation. Forms with identical consonants enter languages’ basic vocabularies less frequently than those without, though not at a rate greater than would be expected from a random sample from a language’s vocabulary for all but one family analyzed. Over the course of word forms’ character histories, processes of word form mutation—a composite of regular sound changes, analogical changes, and in some cases changes to a word family that alter transparent morphological relationships—are more likely to remove sequences of identical consonants than they are to introduce them; however, this effect is not consistently greater than what would be expected under a neutral process of sound change (as operationalized here), and this effect is not detectable within the durations of time when forms serve as basic vocabulary items for most language families under study. Forms with identical consonants are phased out of languages’ basic vocabularies more frequently than those without; however, individual word forms with this pattern (serving in any meaning function) do not die out more frequently than forms without sequences of identical consonants. Word forms containing identical consonants can persist for a long time, likely in meaning functions with lower need probability, despite their communicatively suboptimal characteristics—in some functions, such as child and child-directed speech, such forms may in fact be optimal, given the ease with which children produce homogeneous syllables. Importantly, while competition between forms to fill various niches in the basic vocabulary favors forms without identical consonants (22), there is no evidence that word forms with this pattern are more likely to die out altogether, as claimed by some (7). This dovetails with theories in which language change is not driven solely by cognitive considerations (39, 40); there may be social pressures that favor the retention of lexical items, regardless of the sound patterns they contain.

This finding can be viewed through the lens of evolutionary and developmental perspectives on phenotypic evolution, which tease apart developmental constraints and sorting-related processes in evolutionary trajectories (41, 42). Developmental constraints involve limitations on the space of variants that can be produced, while variants are propagated through sorting-related processes. It is not clear whether the object of investigation of this study, lexical items, is directly analogous to biological notions of a phenotype, as human language itself is argued to be a phenotype (43). Regardless, an important insight is that the evolutionary trajectories of lexical items can be shaped by production biases (i.e., constraints on the creation of certain types) as well sorting-related pressures such as selection or drift (here, mutational processes changing the sound patterns found in word forms as well as extinction processes affecting word forms with different sound patterns). The cross-linguistic underrepresentation of word forms containing identical consonants is overwhelmingly due to a bottleneck in production. To a less pronounced extent not found across all families studied, mutational processes favor the removal of sequences of identical consonants within word forms over their lifetimes. Given the ambiguous nature of these results, we are not in a position to characterize this mutational asymmetry as a selectional process driven by sound changes that explicitly target sequences of identical consonants rather than an epiphenomenon of other change types that happened to remove such sequences in the course of affecting a particular sound in a wider range of contexts (as in the case of Latin bibere > Old French beivre). Furthermore, the method used here detects asymmetric pressures in evolution but may not reliably distinguish between signatures of drift and selection (44, 45). Crucially, after surviving the production bottleneck to conventionalization and being subject to mutational processes, forms with identical consonants have as much longevity as those without, even though they appear to be selected against in the basic vocabulary.

Identical consonant avoidance is one of a number of pressures that shape the evolution of sound patterns across languages. This avoidance itself is a multifaceted phenomenon, applying to different degrees across different sound classes. This issue could not be directly addressed in the current paper due to a lack of a unified phonemic transcription of the data used, but as phonemically normalized etymological datasets grow, it will be increasingly possible to probe the more general phenomenon of similar consonant avoidance (9, 10, 46). Paradoxically, beyond the domain of similarity avoidance, languages are often subject to harmony processes affecting vowels and under some highly specific conditions, consonants (47–50). Future work can help to tease apart how these seemingly contradictory patterns impact specific aspects of change, possibly within a unified phylogenetic model.

Despite the general flexibility human languages have in assigning forms to meaning functions (51), the distribution of sound patterns in languages’ lexicons are still shaped by a drive toward communicative efficiency. However, there are multiple components to this general pressure with roles that remain poorly understood. Phylogenetic approaches like the one employed here have the potential to shed light on the origins and maintenance of other plausibly efficiency-driven phenomena (52–54).

## Materials and Methods

### Cognate Class Traits.

The evolution of cognate classes was analyzed in the Austronesian, Semitic, and Uralic families using data from digitized etymological dictionaries (55–60), which organize etymologically related forms in contemporary languages according to cognate classes and provide a reconstructed ancestral form for each cognate class. For each language in the three datasets, cognate classes were coded according to whether or not they were absent or present (e.g., Latin *manducare* “chew” survives into French as *manger* “eat” but has been lost in Spanish), and if present, whether they contained two adjacent (i.e., separated by a vowel) identical consonants or not.

The search for identical consonants was restricted to sequences which co-occurred within and not across active morpheme boundaries, e.g., boundaries between members of complex words such as compounds (5, 61–63). In the Semitic and Uralic datasets, hyphens were taken to mark active morpheme boundaries in words where they were present. Detecting synchronically active morpheme boundaries was a greater challenge for the Austronesian data, as the Austronesian Comparative Dictionary (ACD) marks affix and infix boundaries that were active in ancestral forms but not necessarily active in the reflexes where they are marked. In order to address this issue, for a group of etymologically related forms in a given language sharing a transparent semantic relationship and a clear derivational relationship, the longest common subsequence was extracted and treated as the basic reflex of the etymon in question.

Each reconstructed etymon in each dataset was aligned with the portion of each corresponding entry most likely to descend from it using an iterative version of the Needleman–Wunsch algorithm (64, 65). Aligned forms were orthographically normalized in order to facilitate the automatic extraction of the presence of identical consonants separated by a single vowel. Each cognate class according to the states {absent, −IC, +IC} in each language. In some cases, a cognate class attests both of the states −IC and +IC in a given language, if the language attests synchronically unrelated reflexes of an etymon with and without identical consonants. Datasets were converted into likelihood matrices, setting state values for a given etymon in a given language to 1 and all unattested values to 0. For phylogenetic comparative analyses conducted on these datasets, published tree samples of the Austronesian, Semitic, and Uralic families were used (66–68).

A risk of using etymological dictionaries is that the absence of a cognate class coded for a language may be due to artifacts of the etymologization process, and not because the cognate class died out in the language. To ensure that well-etymologized languages and secure cognate classes were used for analyses, datasets contain only languages with more than 250 reflexes in the etymological database in which they are found and cognate classes found in more than 10% of languages in a given family. The Austronesian dataset consisted of 1,693 cognate sets from 54 languages. The Semitic dataset consisted of 1,378 cognate sets in 23 languages. The Uralic dataset consisted of 1,872 cognate sets in 15 languages.

### Phylogenetic Analysis of Cognate Class Traits.

Cognate class traits were assumed to evolve over phylogenies according to a continuous-time Markov (CTM) chain, a stochastic process where between-state transitions take place according to transition rates. A crucial difference between comparable biological phenomena (69, 70) and cognate class traits is that cognate classes are nonhomoplastic; they are generally born once on a phylogeny (except in the case of extensive borrowing or parallel derivational processes) and cannot be revived once they die out, in the absence of a strong philological tradition similar to that of contemporary times. In order to ensure that the evolutionary model used has the single-birth behavior described above, I use a modified version of the Stochastic Dollo model of character evolution (71, 72) that does not suffer from well-known problems of this method, in that the initial character state is independent of the character’s long-term behavior, and the likelihood of *D* attested cognate classes under a phylogeny Ψ and evolutionary rate parameters Q, $\prod_{d=1}^{D}P\left(x_{d}|\Psi ,Q\right)$ can be efficiently computed using the standard pruning algorithm (73). The model used in this paper satisfies the single-birth criterion by allowing transitions from the state absent to the states ±IC but not from the states ±IC to the state absent on potential birth loci, i.e., branches ancestral to the most recent common ancestor (MRCA) of all languages where the cognate class is present, and from ±IC to absent but not absent to ±IC on all other branches. This ensures that a cognate class will be born once on a phylogeny, and not be revived once it dies out.

Reconstructions in the etymological resources used were generated by experts via careful application of the comparative method of historical linguistics; therefore, care was taken to ensure that the initial state (±IC) of each cognate class character matched the presence or absence of identical consonants in the reconstructed form. This involved grafting a branch of infinitesimal length to the MRCA of all languages where the cognate class is present leading to a node containing the state found in the expert reconstruction. Additionally, transitions between the states ±IC were not allowed on birth loci, ensuring that the birth state of each cognate class matched the state found at the tip of the grafted branch.

For an individual cognate class with index d∈{1,...,D}, transitions between the states {absent, −IC, +IC} take place according to the following rate matrix on birth loci (diagonal cells are equal to the negative sum of off-diagonal cells in the same row):

$$Q_{d}^{b}=\left(\begin{array}{ccc} \text{–} & \lambda_{d}^{-} & \lambda_{d}^{+}\\ 0 & \text{–} & 0\\ 0 & 0 & \text{–} \end{array}\right)$$

On nonbirth loci, the rate matrix takes the following form:

$$Q_{d}^{\neg b}=\left(\begin{array}{ccc} \text{–} & 0 & 0\\ \mu_{d}^{-} & \text{–} & \rho_{d}^{-+}\\ \mu_{d}^{+} & \rho_{d}^{+-} & \text{–} \end{array}\right)$$

The birth rate parameters $\lambda_{d}^{-}$ and $\lambda_{d}^{+}$ represent transitions from the state absent to the states −IC and +IC, respectively; $\rho_{d}^{-+}$ and $\rho_{d}^{+-}$ represent transitions between the states −IC and +IC; and $\mu_{d}^{-}$ and $\mu_{d}^{+}$ represent transitions from the states −IC and +IC, respectively, to the state absent. As in other modifications to the Stochastic Dollo model (74), cognate traits cannot be born again after they have been active and lost.

Because cognate classes are born only once, the birth rates $\lambda^{-}$ and $\lambda^{+}$ are kept invariant across cognate classes. The remaining evolutionary parameters, which pertain to the evolution of cognate classes once they are born, are allowed to vary according to a hierarchical model for each cognate class d∈{1,...,D}, since individual cognate classes may have different evolutionary trajectories. According to this model, cognate class-specific transition rates are composed of a global rate and a local cognate class-specific multiplier that allows rates to vary across classes as needed. Rates are distributed as described below.

Priors over the parameters $\lambda_{0}^{-}$, $\lambda_{0}^{+}$, $\rho_{0}^{-+}$, $\rho_{0}^{+-}$, $\mu_{0}^{-}$, $\mu_{0}^{+}$, which represent log mean rates around which cognate class-level rates are distributed, follow the standard normal distribution. For a given cognate class with indexd∈{1,...,D}, evolutionary rates have the following form. The global birth rates are transformed via an exponential link function:


$$\begin{array}{l} \lambda_{d}^{-}=\text{exp}(\lambda_{0}^{-})\\ \lambda_{d}^{+}=\text{exp}(\lambda_{0}^{+}) \end{array}$$

The remaining transition rates are log-normally distributed:


$$\begin{array}{ll} \rho_{d}^{-+}\left\{ \begin{array}{l} \sim \text{LogNormal}(\rho_{0}^{-+},\sigma^{\rho^{-+}})\\ =0 \end{array}\right. & \begin{array}{l} \text{if }x_{d}\in \{\text{absent},\;-\text{IC},\text{+IC}\}\\ \text{otherwise} \end{array}\\ \rho_{d}^{+-}\left\{ \begin{array}{l} \sim \text{LogNormal}(\rho_{0}^{+-},\sigma^{\rho^{+-}})\\ =0 \end{array}\right. & \begin{array}{l} \text{if }x_{d}\in \{\text{absent},\;-\text{IC},\text{+IC}\}\\ \text{otherwise} \end{array}\\ \begin{array}{l} \mu_{d}^{-}\sim \text{LogNormal}(\mu_{0}^{-},\sigma^{\mu^{-}})\\ \mu_{d}^{+}\sim \text{LogNormal}(\mu_{0}^{+},\sigma^{\mu^{+}}) \end{array} & \end{array}$$

HalfNormal(0,1) priors are placed over SD parameters σ. Not all cognate classes attest all three states; some only express the pairs of states (abs,−IC) and (abs,+IC). These characters do not provide information that bears on transitions between the states −IC and +IC, but provide information regarding the birth rates and death rates of cognate classes displaying these patterns. For characters of this sort, transitions to and from the unattested state are set to zero, as shown above.

The likelihood of each trait $P(x_{d}|\Psi ,Q_{d})$ was corrected for ascertainment bias. This correction is intended to account for the fact that the observed cognate classes represent only a fraction of the cognate classes that have existed during the course of each family’s history, as many will have died out before being recorded (73, 75–77). This amounts to conditioning the trait likelihood on the probability that the trait would be observed in the first place under the CTM process that governs its evolution. The corrected likelihood is equal to the following:

$$\frac{P(x_{d}|\Psi ,Q_{d})}{1-P(x_{\text{abs}}|\Psi ,Q_{d})}$$

Above, $x_{\text{abs}}$ represents a trait likelihood matrix with the value absent for all tips in the phylogeny. For comparability between $P(x_{d}|\Psi ,Q_{d})$ and $P(x_{\text{abs}}|\Psi ,Q_{d})$, $x_{\text{abs}}$ is augmented to contain a tip descending from a branch of infinitesimal length grafted to the MRCA of all languages where the cognate class is present, the value of which is equal to the reconstructed value.

### Baselines for Cognate Class Traits.

#### Baseline birth rates of cognate class traits.

Under a process where sequences are generated by randomly sampling consonants from the uniform distribution, the probability of generating a sequence *w* containing at least two adjacent identical consonants is equal to the following, where |w| denotes sequence length and +IC∈w indicates the presence of adjacent identical consonants within a sequence:

$$P\left(+\text{IC}\in w\right)=\sum_{i=1}^{N}P\left(|w|=i\right)P\left(+\text{IC}\in w;|w|=i\right).$$

In a language with *S* segments, $P\left(+\text{IC}\in w;|w|=2\right)=\frac{1}{S}$. Since the probability of generating a sequence containing at least two adjacent identical consonants is higher for longer sequences, P(+IC∈w) will be higher when $P\left(|w|=i\right)=\frac{1}{N}$ for all i∈{1,...,N}. Assuming that shorter sequences are more frequently generated than longer ones, we expect this quantity to approach $\frac{1}{S}$ as P(|w|=2) approaches 1, allowing us to derive a lower bound $P\left(+\text{IC}\in w\right)\geq \frac{1}{S}$. The expected ratio between words without and words with identical consonants will then be less than or equal to S−1. In the case of a theoretical language requiring that a minimal word consist of more than two consonants, this ratio will be even smaller. Numbers of consonants for languages in each family (Afro-Asiatic was taken as a proxy for Semitic) were taken from the PHOIBLE database (78).

#### Baseline +IC →−IC vs. −IC →+IC mutation rates.

A simulation procedure was used to estimate the frequencies at which neutral models of sound change are expected to introduce sequences of identical consonants into lexical items versus remove them. Frequencies of such changes depend on existing frequencies of sound patterns found across the lexicon. To ensure that frequencies of word lists to which simulated sound changes were realistic, word lists from languages in each dataset were used (simulations were applied to languages with 500 or more entries). Unconditioned, word-initial, word-medial, and word-final changes were extracted from a compendium of consonantal sound changes (21) and modified to ensure that they would apply to the datasets used in this paper. For each language, a rule capable of applying to forms in the language was chosen at random and applied to the language’s word list, substituting the output segment for the input segment in the relevant environment. Subsequently, the number of changes removing versus introducing tautomorphemic sequences of IC were tabulated, and a ratio computed by dividing the former number by the latter number (with a smoothing constant of 1 added to each number to prevent zero division). This procedure was carried out 500 times per language, with ratios averaged at the language level.

### Cognate-Concept Traits.

The evolution of cognate-concept (alternatively root-meaning) traits (77, 79, 80) was analyzed using data from a subset of the Lexibank repository (81) that has been processed to normalize orthographic forms and link records in different languages to the Concepticon semantic taxonomy (82). Data from five families were analyzed. These were Dravidian (83–85), Indo-European (77, 86, 87), Sino-Tibetan (88, 89), Turkic (90, 91), and Uto-Aztecan (92).

Forms in different languages were automatically coded according to whether or not they contained a sequence of identical consonants separated by a single vowel within morpheme boundaries (demarcated by the symbol +). This was relatively straightforward thanks to the space-delimited orthographic normalization of forms. The cross-linguistic transcription systems (CLTS) database (93) was used to determine which segments in each string were consonants. The geminate marker: was stripped from geminate segments and sequences of identical segments were simplified to one segment before a script was used to detect the presence of adjacent identical consonants within morphological boundaries.

A language expresses a given semantic concept using formal material corresponding to one or more cognate classes, in which sequences of identical consonants can be present or absent. For instance, Portuguese expresses the concept DRINK with the form /bibeR/, which contains identical consonants and is a reflex of the Proto-Indo-European etymon *peh_3_-. Thus, for each language in a family, cognate-concept traits are coded according to the states {absent,−IC,+IC}.

Cognate-concept characters for different families were transformed into binarized likelihood matrices. In the case of lexical polymorphism (i.e., in which a language attests multiple forms for a meaning), multiple likelihoods were set to one. Analyses were restricted to data corresponding to 100 basic concepts (29) available through Concepticon (82). Concept-level basicness rankings were taken from NorthEuraLex v. 0.9 (94). Cross-linguistic frequency values (36) were available via Concepticon (82). The Dravidian dataset consisted of 709 concept-cognate traits corresponding to 93 concepts from 20 languages. The Indo-European dataset consisted of 686 concept-cognate traits corresponding to 96 concepts from 19 languages. The Sino-Tibetan dataset consisted of 1517 concept-cognate traits corresponding to 83 concepts from 44 languages. The Turkic dataset consisted of 225 concept-cognate traits corresponding to 90 concepts from 31 languages. The Uto-Aztecan dataset consisted of 1,087 concept-cognate traits corresponding to 92 concepts from 33 languages.

### Phylogenetic Analysis of Cognate-Concept Traits.

Cognate-concept traits were modeled as evolving according to a CTM process. Since they are homoplastic (i.e., a cognate class can come to express the same meaning independently on two different lineages), standard models used to analyze morphologically dependent traits are applicable without the need to account for the single-birth criterion. As above, hierarchical models were used to jointly analyze the evolution of cognate-concept traits jointly within separate families. Transition rates were assumed to vary at the concept level; the likelihood for a given cognate-concept trait with index d∈{1,...,D} under a phylogeny Ψ, $P(x_{d}|\Psi ,Q_{\text{concept}[d]})$ depends on the transition rates for the concept which the trait expresses and can be computed using the pruning algorithm.

For each concept c∈{1,...,C}, transitions between the states {absent, −IC, +IC} take place according to the following rate matrix:

$$Q_{c}=\left(\begin{array}{ccc} \text{–} & \lambda_{c}^{-} & \lambda_{c}^{+}\\ \mu_{c}^{-} & \text{–} & \rho_{c}^{-+}\\ \mu_{c}^{+} & \rho_{c}^{+-} & \text{–} \end{array}\right)$$

Here, all rates (including the birth rates $\lambda^{-}$ and $\lambda^{+}$) vary across concepts, since cognate-concept traits are homoplastic, and concept-cognate traits for certain concepts may arise more frequently than for others.

Priors over the parameters $\lambda_{0}^{-}$, $\lambda_{0}^{+}$, $\rho_{0}^{-+}$, $\rho_{0}^{+-}$, $\mu_{0}^{-}$, $\mu_{0}^{+}$, which represent log baseline rates, follow the standard normal distribution. For a given concept with index c∈{1,...,C}, evolutionary rates are distributed as follows:


$$\begin{array}{l} \lambda_{c}^{-}\sim \text{LogNormal}(\lambda_{0}^{-},\sigma^{\lambda^{-}})\\ \lambda_{c}^{+}\sim \text{LogNormal}(\lambda_{0}^{+},\sigma^{\lambda^{+}})\\ \rho_{c}^{-+}\sim \text{LogNormal}(\rho_{0}^{-+},\sigma^{\rho^{-+}})\\ \rho_{c}^{+-}\sim \text{LogNormal}(\rho_{0}^{+-},\sigma^{\rho^{+-}})\\ \mu_{c}^{-}\sim \text{LogNormal}(\mu_{0}^{-},\sigma^{\mu^{-}})\\ \mu_{c}^{+}\sim \text{LogNormal}(\mu_{0}^{+},\sigma^{\mu^{+}}) \end{array}$$

HalfNormal(0,1) priors are placed over SD parameters σ. The rate parameters for concept-cognate trait d∈{1,...,D} are equal to the rate parameters for concept[d], if $x_{d}$ attests all three states {absent,−IC,+IC}; otherwise, $\rho_{d}^{-+}$ and $\rho_{d}^{+-}$ are set to zero, as in the previous study.

Trait likelihoods were corrected for ascertainment bias in the manner described above. Here, $x_{\text{abs}}$ represents a trait likelihood matrix with the value absent for all tips in the phylogeny.

### Baselines for Cognate-Concept Traits.

#### Baseline birth rates of cognate class traits.

Under a null model in which basic vocabulary items are sampled from the general (i.e., basic and nonbasic) vocabulary at random with no sensitivity to the sound patterns displayed by individual forms, the ratio of birth rates of cognate-concept traits without versus with sequences of identical consonants should be comparable to the ratio between forms without and with identical consonants in the lexicon from which basic vocabulary items are sampled. These ratios are estimated for languages in each family under study on the basis of large word lists comprising basic as well as nonbasic items. Dravidian, Indo-European, and Turkic ratios were estimated from NorthEuraLex (94). Sino-Tibetan ratios were estimated from the Sino-Tibetan Etymological Dictionary and Thesaurus (95). Uto-Aztecan ratios were estimated from available digitized resources for Nahuatl (96), Yaqui (97), and the Bridgeport variety of Northern Paiute (98). For each language, the number of forms lacking sequences of identical consonants was divided by the number of forms containing sequences of identical consonants.

#### Baseline +IC →−IC vs. −IC →+IC mutation rates.

This simulation procedure was carried out as described for cognate class traits, with the difference that sound changes were applied only to the 100 basic vocabulary items under analysis rather than larger word lists.

### Inference.

Data were processed using Python 3 as well as version 0.6-99 of the R package phytools (99). Models were fitted using RStan version 2.26.13 (100), running the No U-Turn Sampler (NUTS) over 4 chains for 2,000 iterations, with the first half discarded as burn-in. Model convergence was assessed via the potential scale reduction factor (101), with values under 1.1 taken to indicate convergence. To incorporate phylogenetic uncertainty, the model was run on 25 trees from each tree sample and the resulting posterior samples for runs that reached convergence were concatenated together, yielding 100,000 samples per model. 95% HDIs were computed using the R package HDInterval (102).

## Supplementary Material

Appendix 01 (PDF)

## Data Availability

Databases, code, and summarized model fits have been deposited in https://github.com/chundrac/idcc (103).
