# Supplementary material for: Multiple evolutionary pressures shape identical consonant avoidance in the world’s languages
Source: Proc Natl Acad Sci U S A. 2024 Jun 25;121(27):e2316677121. doi: 10.1073/pnas.2316677121 (PMC11228491; doi:10.1073/pnas.2316677121)
Supplement: Supplementary file 1 — Appendix 01 (PDF) [file pnas.2316677121.sapp.pdf]

Supplementary information for “Multiple evolutionary pressures shape identical consonant avoidance in the world’s languages”

Chundra A. Cathcart[a,b,c]

[a]Department of Comparative Language Science, University of Zurich
[b]Center for the Interdisciplinary Study of Language Evolution, University of Zurich
[c]DFG Center “Words, Bones, Genes, Tools”, University of Tübingen

April 16, 2024

Contents

1 Materials and methods **2**
1.1 Cognate class traits . . . 2
1.1.1 Delimiting the search domain for IC . . . 4
1.1.2 Data processing workflow . . . 5
1.1.2.1 Alignment . . . 5
1.1.2.2 Further processing . . . 5
1.1.2.2.1 Austronesian . . . 6
1.1.2.2.2 Uralic . . . 7
1.1.2.2.3 Semitic . . . 7
1.1.2.3 Preparation for phylogenetic analysis . . . 8
1.1.3 Phylogenetic analysis of cognate class traits . . . 8
1.1.4 Baselines for cognate class traits . . . 15
1.1.4.1 Baseline birth rates of cognate class traits . . . 16
1.1.4.2 Baseline +IC → −IC vs. −IC → +IC mutation rates . . . 17
1.2 Cognate-concept traits . . . 18
1.2.1 Data processing . . . 19
1.2.2 Preparation for phylogenetic analysis . . . 19
1.2.3 Phylogenetic analysis of cognate-concept traits . . . 26
1.2.4 Baselines for cognate-concept traits . . . 27

1.2.4.1 Baseline birth rates of cognate class traits 27
1.2.4.2 Baseline +IC → −IC vs. −IC → +IC mutation rates 27
1.3 Inference 27

2 Results **28**
2.1 Cognate class traits 28
2.2 Cognate-concept traits 31
2.2.1 Inspection of concept-level rates 34

Appendices **36**

Appendix A HDIs of concept-level transition rate ratios **38**

Appendix B Simulation-based validation **52**

References **54**

1 Materials and methods

All code used in analyses can be found at `https://github.com/chundrac/idcc`. Code accompanied by fitted models can be accessed via a link available in the README of the github repository.

1.1 Cognate class traits

The evolution of cognate classes was analyzed in three families, Austronesian, Semitic, and Uralic. The range of available resources for the study of the evolution of cognate classes is somewhat limited, since electronic databases of cognacy (irrespective of semantics) are greatly outnumbered by electronic databases of cognate-concept pairings (which denote whether, e.g., French *manger* 'eat' is cognate to Italian *mangiare* 'id.' versus Spanish *comer* 'id.'). Resources of the former type are not only rare, but also vary in terms of reliability and coverage. This study uses the Austronesian Comparative Dictionary [1], available through Lexibank [2]; the online Semitic Etymological Database Online [3]; and Uralonet [4, 5]. These resources vary in size. The ACD is arguably the largest and most thorough resource. The SED is currently incomplete but contains significant coverage. Uralonet is based on a soon to be superseded etymological dictionary [6], and has been criticized at times for a tendency to unify etymologically unrelated forms under the same cognate class labels on the basis of debatable semantic connections [7]. Given this shortcoming, there is the possibility that idiosyncratic behavior found in results based on Uralic data is an artifact of data quality rather than indicative of variation in evolutionary behavior. The variable nature of

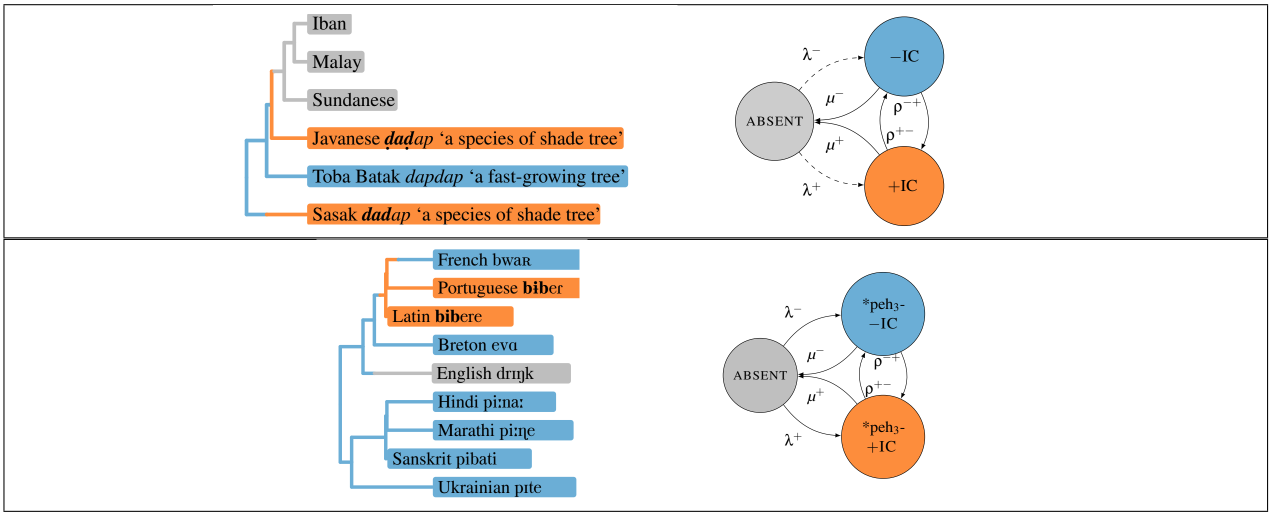

Figure SI 1: Schemata of continuous-time Markov models of evolution for a cognate class trait representing the Proto-Malayo-Polynesian etymon *dapdap (above) and a cognate-concept trait representing whether languages use the Proto-Indo-European root *peh$_3$- in the meaning ‘drink’ (below). Both trait types undergo transitions between states representing absence, presence without identical consonants, and presence with identical consonants. Tree branch colors represent hypothetical but unobserved character histories (i.e., evolutionary trajectories) involving transitions between states. Transition rates (representing frequencies of transitions between states) can be inferred on the basis of (1) data attested in languages and (2) language phylogenies. Parameters governing the evolution of the traits given here can be subdivided into birth rates λ_0^-, λ_0^+ (transitions from ABSENT to ±IC), rates involving mutations introducing or removing sequences of identical consonants ρ_0^{-+}, ρ_0^{+-} (transitions between ±IC), and loss rates μ_0^-, μ_0^+ involving the death of cognate classes or concept-cognate traits (transitions from ±IC to ABSENT). The dashed lines in the schema in the top panel represent the understanding that cognate classes are born only once.

these data sets is unavoidable, given the sparsity of digitized etymological data sets, and potential issues concerning coverage and quality are taken into account when interpreting model results. As it happens, the models employed by this paper show relatively consistent behavior across the three families.

These three resources organize etymologically related forms in contemporary languages according to cognate classes and provide a reconstructed etymon (i.e., ancestral form) for each cognate class. In some cases, etyma are reconstructed to subgroups within the tree (e.g., Proto-Malayo-Polynesian within Austronesian) but are not thought by experts to be ancestral to the family as a whole. The SED marks certain reconstructions as areal, thought to have spread among geographically proximate Semitic languages via language contact. The three resources make different distinctions in terms of the sets of forms that are treated as belonging a cognate class. The ACD assigns forms in Austronesian languages to cognate classes at both the root and word level. To illustrate this distinction, Acehnese *lakòë* 'husband', Tagalog *laláke* 'man, an adult male; male, masculine' and Malagasy *laláhy* 'man (provincial)' are all cognates at the root level, reflecting Proto-Malayo-Polynesian *laki, but only the latter two forms are cognates at the word level, descending from derived Proto-Western-Malayo-Polynesian *la-laki). Uralonet and the SED do not make this distinction and code cognacy at the root level alone.

For each language in the three data sets, root-level cognate classes were coded according to whether or not they were absent or present (e.g., Latin *manducare* 'chew' survives into French as *manger* 'eat' but has been lost in Spanish), and if present, whether it contained two adjacent (i.e., separated by a vowel) identical consonants or not. This yields three states that a language can express for a given cognate class: ABSENT, +IC, and −IC.

1.1.1 Delimiting the search domain for IC

The search for identical consonants was restricted to sequences which co-occurred within and not across active morpheme boundaries (e.g., boundaries between members of complex words such as compounds), since a number of key generalizations regarding identical consonant avoidance make reference to tautomorphemic violations of this constraint [8, 9, 10, 11]. In Austronesian languages in particular, co-occurrence rates of consonants with identical place of articulation differ across tautomorphemic and heteromorphemic contexts, given the frequent occurrence of reduplication and infixation processes that create identical adjacent consonants in derived forms [12, 13]. Accordingly, models may infer different degrees of diachronic tolerance for identical consonants, depending on whether only tautomorphemic sequences are taken into consideration. For this reason, care was taken to code for the presence of such sequences only within the appropriate subword unit

associated with a given form.

In the Semitic and Uralic data sets, hyphens were taken to mark active morpheme boundaries in words where they were present. Detecting synchronically active morpheme boundaries was a considerably greater challenge for the Austronesian data, as the ACD marks affix and infix boundaries that were active in ancestral forms but not necessarily active in the reflexes where they are marked. As an example, the ACD gives the Aklanon word for ‘woman’ as *ba-báyi* on the basis of reduplicated Proto-Austronesian *ba-bahi, even though a morpheme boundary is not marked in the source from which the word is taken [14] and the form is presumably synchronically tautomorphemic, as there are no other related forms that would facilitate the abstraction of a base *báyi*. Coding only the presence of identical consonants within hyphen-delimited forms after stripping out infixes runs the risk of severely under-counting tautomorphemic violations of IC avoidance. In order to address this issue, for a group of etymologically related forms in a given language that share a transparent semantic relationship and a clear derivational relationship (e.g., Javanese *niṭik* ‘to strike a light using flint and steel’ and *ṭiṭik* ‘flint and steel for starting fires’ < PAN *tiktik), the longest common subsequence was extracted (here *iṭik*) and treated as the basic reflex of the etymon in question.

1.1.2 Data processing workflow

1.1.2.1 Alignment Each data set divides up reflex words of each cognate class into hyphen-delimited subword units (which in most cases can be interpreted as morphemes). Each reconstructed etymon in each dataset was aligned with the subword portion of each corresponding entry most likely to descend from it using an iterative version of the Needleman-Wunsch algorithm [15, 16]. In the case of reflexes consisting of multiple space-delimited words, the word most likely to descend from the etymon was aligned with the etymon as well. The purpose of this procedure was to minimize the risk of extracting the presence of identical consonants in an element not homologous with the etymon whose evolution is being tracked. Family-level alignment scripts are found at the following URLs: `https://github.com/chundrac/idcc/blob/main/ACD_processing/process_data/align_forms.py`, `https://github.com/chundrac/idcc/blob/main/SED_processing/process_data/align_forms.py`, `https://github.com/chundrac/idcc/blob/main/Uralonet_processing/process_data/align_forms.py`

1.1.2.2 Further processing Following the alignment process, additional steps were needed to facilitation the extraction of the features used in phylogenetic modeling. This involved orthographic normalization and in the case of Austronesian, the extraction of subsequences. Workflows different

slightly across the data sets used, given their varying nature. In general, the process for each data set was largely heuristic and involved both consultation of sources on the languages in the data set as well as automated processes such as profiling orthographic characters based on the contexts in which they occur.

1.1.2.2.1 Austronesian Strings were tokenized in order to delimit groups of characters representing segments by whitespace. This necessitated combining certain graphical elements together. As initial tokenization steps, digraphs occuring in the data were identified, along with characters combining with the previous or following characters (e.g., representing aspiration, prenasalization, etc.). Characters were merged together into segments delimited by white space. Geminate sequences (e.g., *bb*) were simplified (e.g., to *b*) so that they could be identified with their singleton counterparts. Under most theoretical views, a sequence like *babba* contains two instances of *b* separated by a single vowel, even though the latter is geminate.

The aligned word elements of all reflexes of a given etymon in a language were collected. If there was only one reflex of an etymon in a language, then the aligned word was taken as string in which to search for sequences of identical consonants. In cases where an etymon had more than one reflex in a language, a procedure was carried out to extract the longest common substring of consonants. This involved converting each word string to a normalized Unicode NFD representation. Elements delimited by the characters <> (representing historical and in some cases synchronic infixes) were deleted. In word strings, hyphens (representing historical and in some cases synchronic morpheme boundaries) were deleted and geminate consonants separated by hyphens were simplified to singleton consonants. Subsequently, vowels were removed from each string, and the longest substring common to all reflexes was extracted. If there was no common substring of a length greater than zero, each aligned subword string was treated as a candidate string in which to search for identical consonants. A worked example follows, involving reflexes of root cognate ID 30416 (Proto-Malayo-Polynesian *maja 'dry') in Nakanai (naka1262) found in Table 1: the longest common

Table SI 1: Selected reflexes of root cognate ID 30416 (Proto-Malayo-Polynesian *maja 'dry') in Nakanai (naka1262)

Word cognate ID	Reflex	Gloss
7550	ma-mara	'to boil away or dry up, of water'
7550	ma-mara-ti	'dammed up, of water'

consonantal substring extracted is ***m m r***, which contains a sequence of identical consonants that

would not be detected if only the hyphen-delimited subword string *mara* were considered. The logic here is that given these etymologically related forms provide no evidence from which a subword unit *mara* can be abstracted. It is of course possible that listeners will be able to infer the presence of derivational morphology or derivational processes on the basis of unrelated groups of lexical items, but the forms present in the database do not facilitate an exhaustive analysis on the cues to morphological boundaries across sets of etymologically related reflexes, nor do they allow for the extraction of underlying representations arrived at via expert analyses that might bear on the presence of identical consonants. The process described here serves as a means of detecting likely morpheme boundaries without undertaking a synchronic morphological analysis of every language in the database. Code involved processing Austronesian data can be found here: `https://github.com/chundrac/idcc/blob/main/ACD_processing/process_data/process_data.py`

1.1.2.2.2 Uralic As above, strings were tokenized in order to delimit groups of characters representing segments by whitespace. As some languages (such as Hungarian, Finnish, and Estonian) are presented in their standard orthography, language-specific digraphs (e.g., Hungarian *cs*, *gy*, etc.) were merged together into individual segments. A number of elements combining with the preceding character were identified, allowing characters to be merged into segments delimited by white space. As above, geminate sequences were simplified so that they could be identified with their singleton counterparts. The hyphen-delimited subword strings aligned with the etyma were taken as the domain from which to extract sequences of identical consonants, after removing all vowels. Code involved processing Uralic data can be found here: `https://github.com/chundrac/idcc/blob/main/Uralonet_processing/process_data/process_data.py`

1.1.2.2.3 Semitic As above, strings were tokenized in order to delimit groups of characters representing segments by whitespace. A number of elements combining with the preceding character were identified, allowing characters to be merged into segments delimited by white space. As above, geminate sequences were simplified so that they could be identified with their singleton counterparts. For some languages, citation forms are presented as consonantal roots, so care was taken to ensure that in consonantal roots, adjacent identical consonants were not conflated with geminates, even though they may surface as geminates in certain inflected forms. The hyphen-delimited subword strings aligned with the etyma were taken as the domain from which to extract sequences of identical consonants, after removing all vowels. Code involved processing Uralic data can be found here: `https://github.com/chundrac/idcc/blob/main/SED_processing/process_data/process_data.py`

1.1.2.3 Preparation for phylogenetic analysis A final processing step for phylogenetic analysis was to convert the data sets into likelihood matrices, setting state values for a given etymon in a given language to 1 and all unattested values to 0. Since languages often attest more than one value for a given etymon, some languages had multiple likelihoods set to one for different etyma. It is worth highlighting that this is a method for dealing with data ambiguity in cladistics rather than actual polymorphism [17]. For phylogenetic comparative analyses conducted on these data sets, published tree samples of the Austronesian, Semitic and Uralic families were used [18, 19, 20]; see Figures 3–5.

To ensure that well-etymologized languages and secure cognate classes were used for analyses, datasets contain only languages with more than 250 reflexes in the etymological database in which they are found and cognate classes found in more than 10% of languages in a given family. The Austronesian data set consisted of 1693 cognate sets from 54 languages. The Semitic data set consisted of 1378 cognate sets in 23 languages. The Uralic data set consisted of 1872 cognate sets in 15 languages. Geographic distributions of families are given in Figure 1.1.2.3. Forms in individual cognate classes plotted on maximum clade credibility trees for each family can be found at `https://github.com/chundrac/idcc/tree/main/cognate_models/data_plots`.

1.1.3 Phylogenetic analysis of cognate class traits

Cognate class traits were assumed to evolve over phylogenies according to a continuous-time Markov (CTM) chain, a stochastic process where between-state transitions take place according to transition rates. A number of biological studies have used CTM models to analyze the evolution of morphologically dependent traits, such as tail color, which is only relevant if a tail is present in a species [22, 23]. A crucial difference between biological phenomena of this sort and cognate class traits is that cognate classes are non-homoplastic; they are generally born once on a phylogeny (except in the case of extensive borrowing or parallel derivational processes), and cannot be revived once they die out, in the absence of a strong philological tradition similar to that of contemporary times.

In order to ensure that the evolutionary model used has the single-birth behavior described above, I use a modified version of the Stochastic Dollo model of character evolution [24, 25] that does not suffer from well-known problems of this method, in that it allows the initial character state to be independent of its long-term behavior and has a likelihood that can be efficiently computed using the standard pruning algorithm [26]. in that the initial character state is independent of the character's long-term behavior, and the likelihood of D attested cognate classes under a phylogeny Ψ and evolutionary rate parameters Q, $\prod_{d=1}^{D} P(x_d|\Psi,Q)$ can be efficiently computed using the

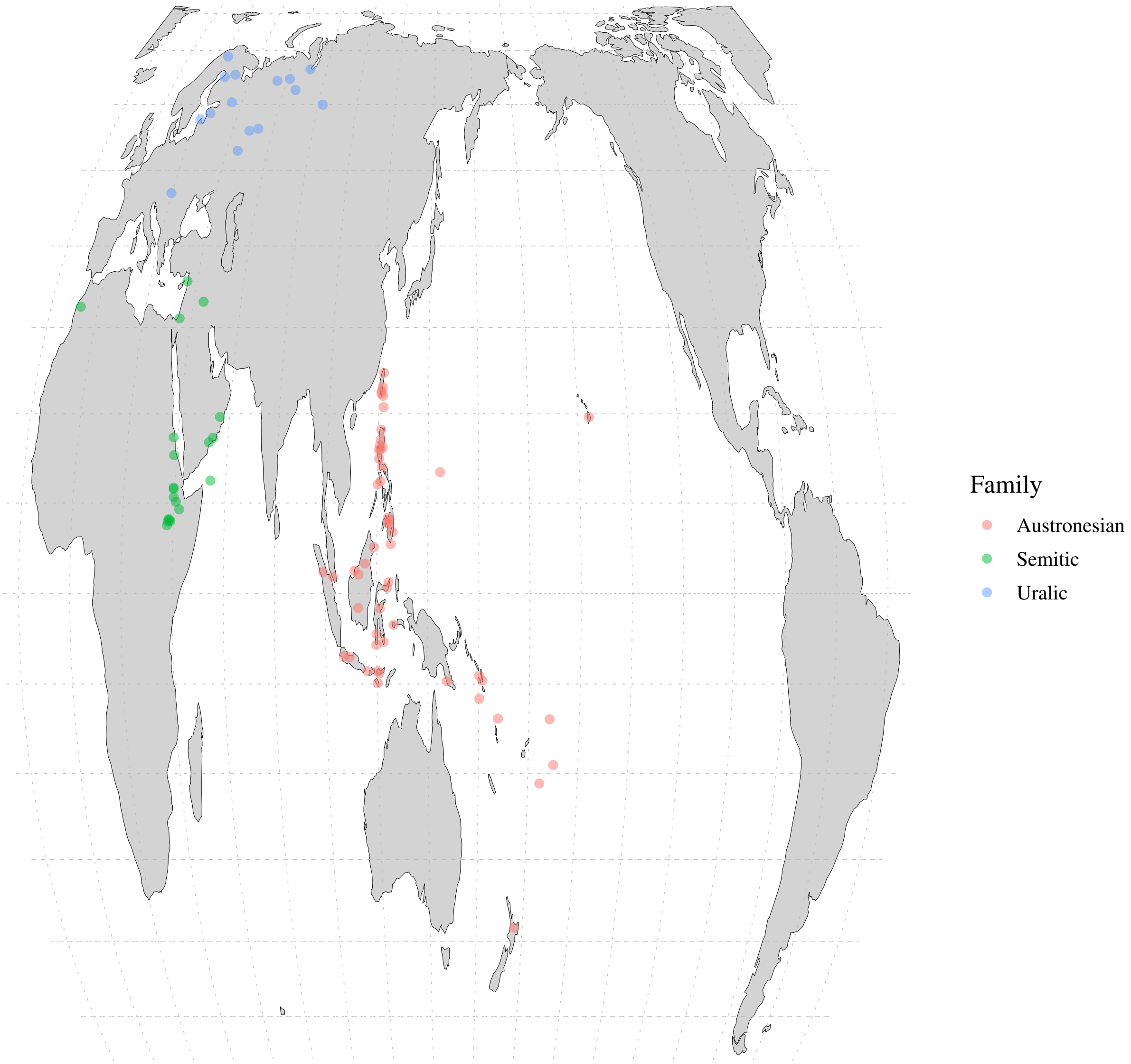

Figure SI 2: Geographic locations of languages used in analyses of cognate class traits, for languages with geographic coordinates in the Glottolog [21] database.

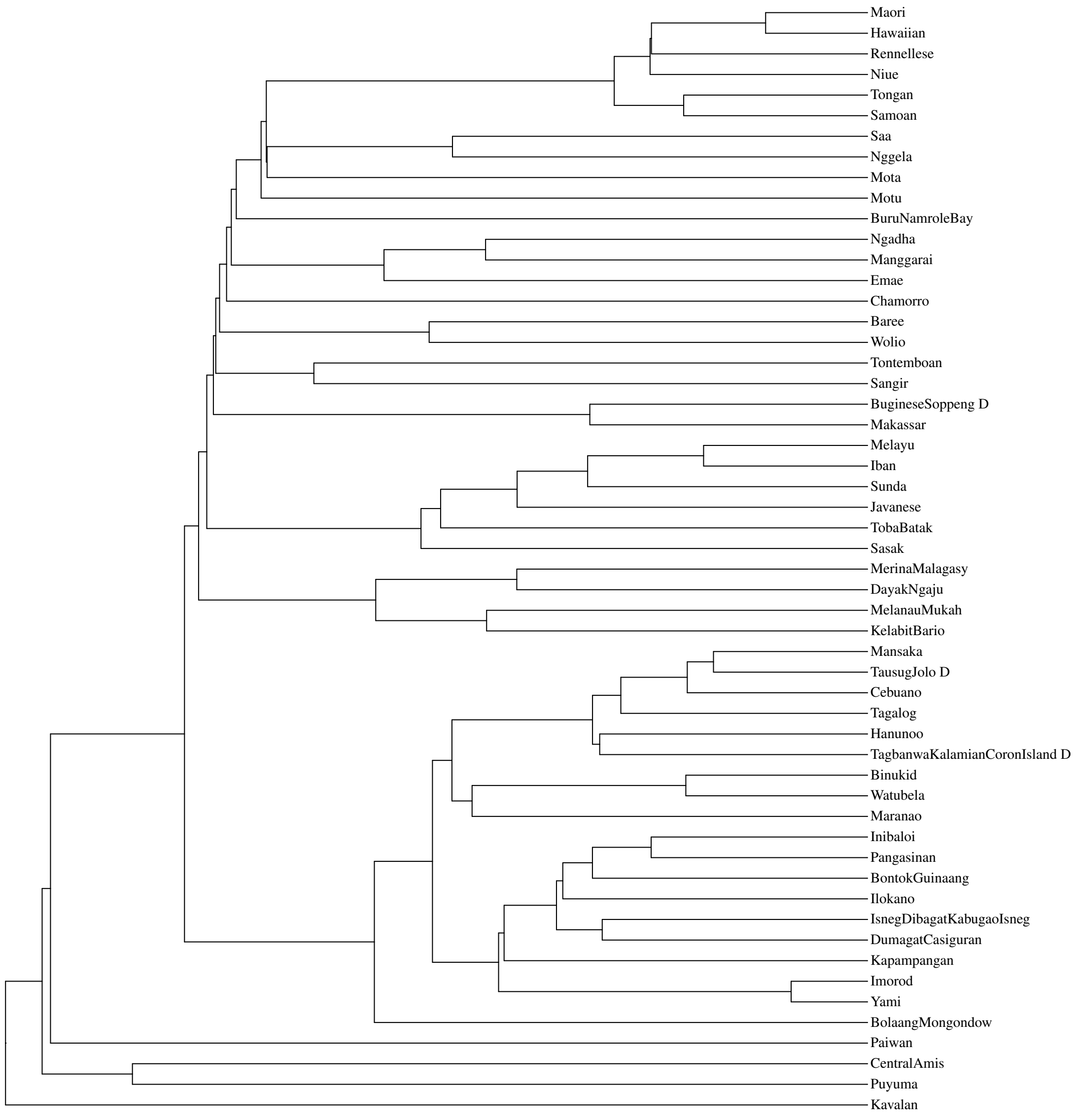

Figure SI 3: Austronesian maximum clade credibility (MCC) tree [18], with languages retained for study.

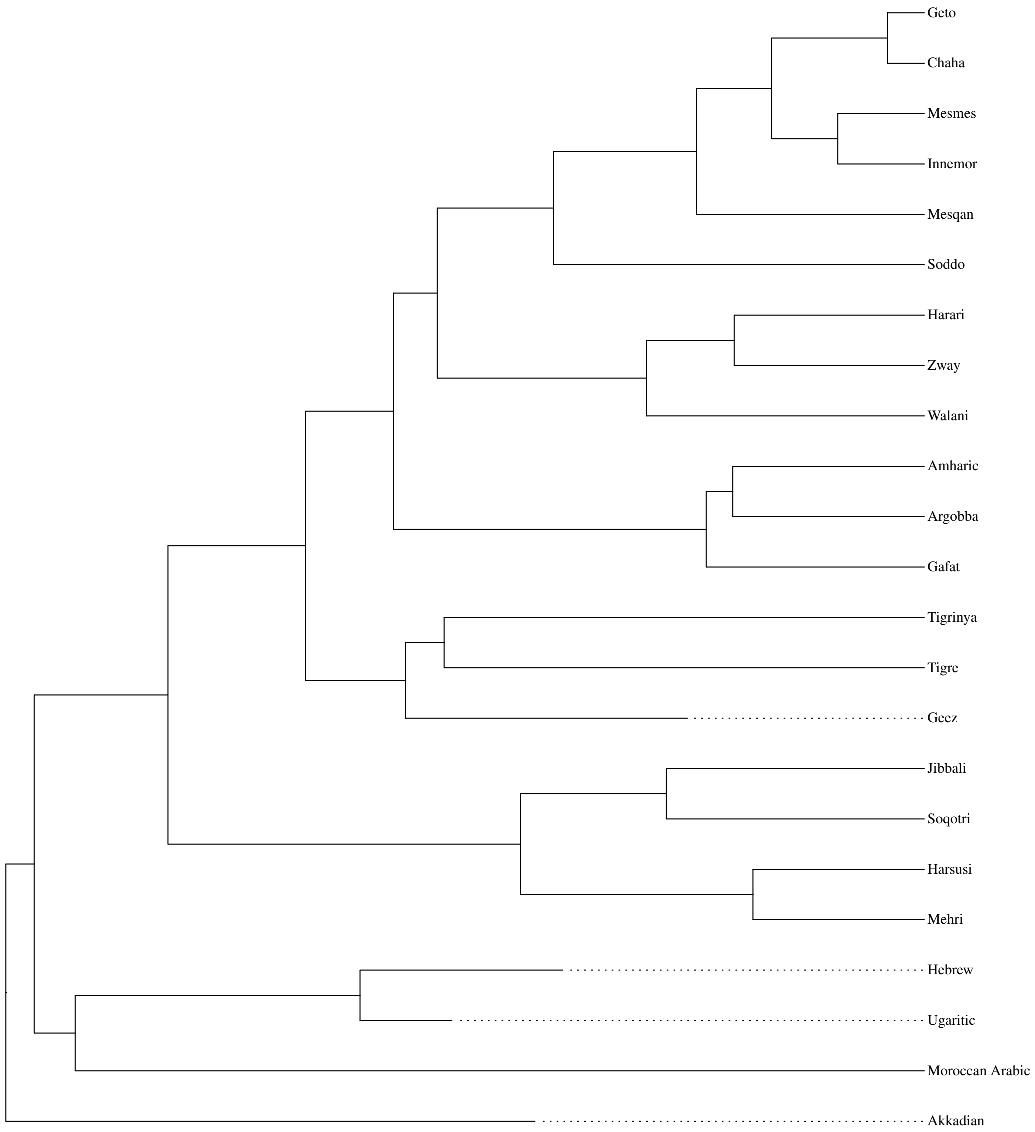

Figure SI 4: Semitic MCC tree [19], with languages retained for study.

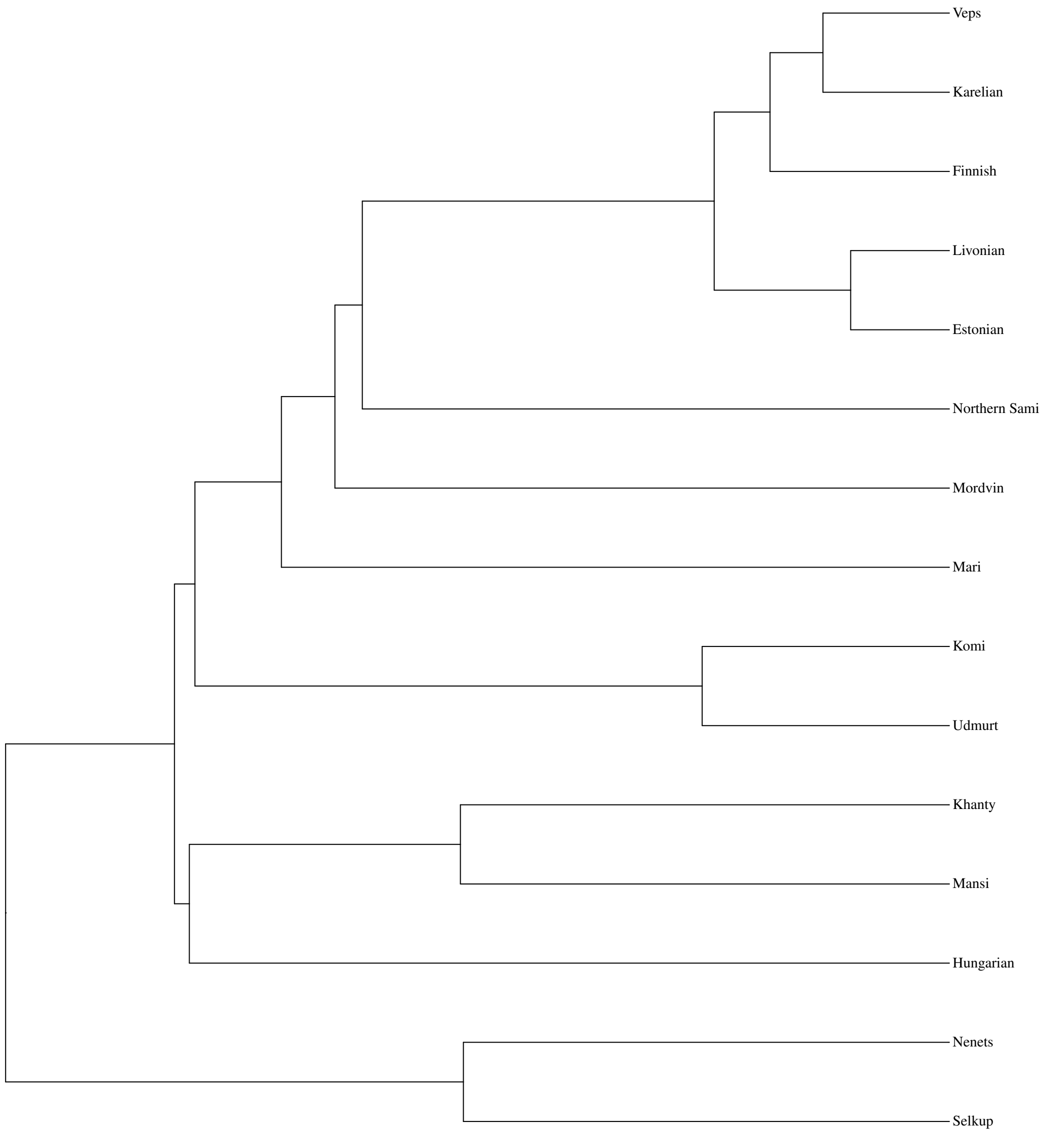

Figure SI 5: Uralic MCC tree [20], with languages retained for study.

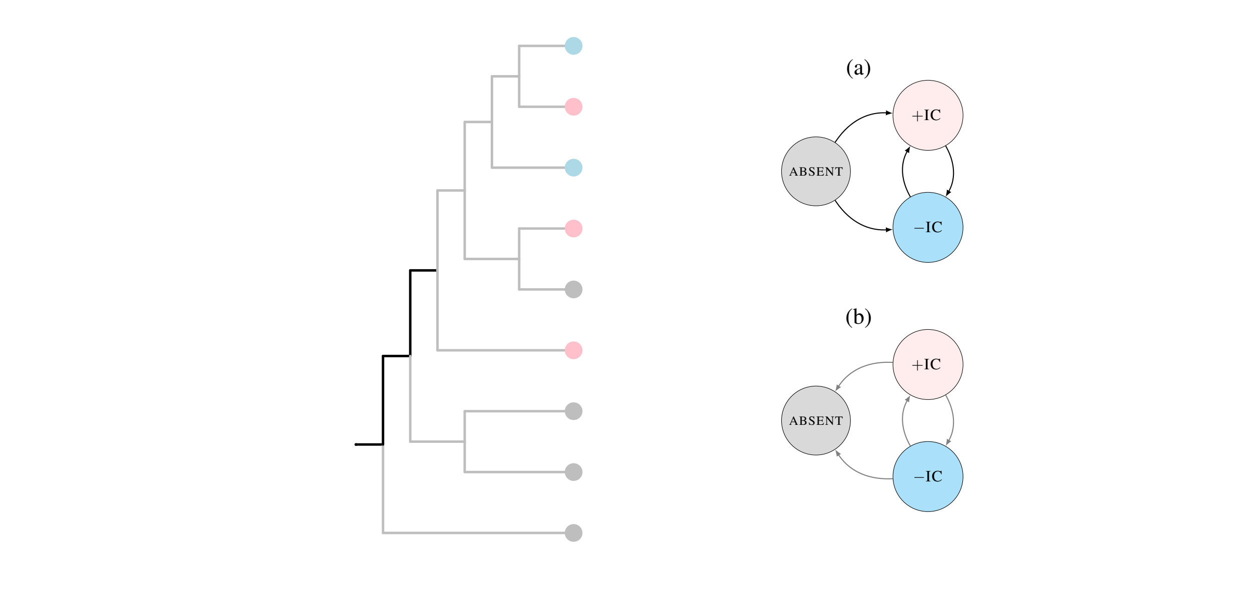

Figure SI 6: Schema of continuous-time Markov model of character evolution used for analyses: (a) transitions from ABSENT to ±IC but not ±IC to ABSENT possible on potential birth loci, i.e., (black) branches ancestral to all languages where the cognate class is present; (b) transitions from ±IC to ABSENT but not ABSENT to ±IC possible on all other (gray) branches.

standard pruning algorithm [26]. The model used in this paper satisfies the single-birth criterion by allowing transitions from the state ABSENT to the states ±IC but not from the states ±IC to the state ABSENT on potential birth loci, i.e., branches ancestral to the most recent common ancestor (MRCA) of all languages where the cognate class is present, and from ±IC to ABSENT but not ABSENT to ±IC on all other branches (Figure 6). This ensures that a cognate class will be born once on a phylogeny, and not be revived once it dies out.

Since the reconstructions found in the etymological resources used were arrived at by experts via careful application of the comparative method of historical linguistics, care was taken to ensure that the initial state (±IC) of each cognate class character matched the presence or absence of identical consonants in the reconstructed form. This involved grafting a branch of infinitesimal length to the MRCA of all languages where the cognate class is present leading to a node containing the state found in the expert reconstruction. Additionally, transitions between the states ±IC were not allowed on birth loci, ensuring that the birth state of each cognate class matched the state found at the tip of the grafted branch.

For an individual cognate class with index $d \in \{1,...,D\}$, transitions between the states $\{$ABSENT, −IC, +IC$\}$ take place according to the following rate matrix on birth loci (diago-

nal cells are equal to the negative sum of off-diagonal cells in the same row):

$$Q_d^b = \begin{pmatrix} — & \lambda_d^- & \lambda_d^+ \\ 0 & — & 0 \\ 0 & 0 & — \end{pmatrix}$$

On non-birth loci, the rate matrix takes the following form:

$$Q_d^{\neg b} = \begin{pmatrix} — & 0 & 0 \\ \mu_d^- & — & \rho_d^{-+} \\ \mu_d^+ & \rho_d^{+-} & — \end{pmatrix}$$

The birth rate parameters λ_d^- and λ_d^+ represent transitions from the state ABSENT to the states $-$IC and $+$IC, respectively; ρ_d^{-+} and ρ_d^{+-} represent transitions between the states $-$IC and $+$IC; and μ_d^- and μ_d^+ represent transitions from the states $-$IC and $+$IC, respectively, to the state ABSENT. As in other modifications to the Stochastic Dollo model [27], cognate traits cannot be born again after they have been active and lost.

Since cognate classes are born only once, the birth rates λ^- and λ^+ are kept invariant across cognate classes. The remaining evolutionary parameters, which pertain to the evolution of cognate classes once they are born, are allowed to vary according to a hierarchical model for each cognate class $d \in \{1,...,D\}$, since individual cognate classes may have different evolutionary trajectories. According to this model, cognate class-specific transition rates are composed of a global rate and a local cognate class-specific multiplier that allows rates to vary across classes as needed. Rates are distributed as described below.

Priors over the parameters λ_0^-, λ_0^+, ρ_0^{-+}, ρ_0^{+-}, μ_0^-, μ_0^+, which represent log mean rates around which cognate class-level rates are distributed, follow the standard normal distribution. For a given cognate class with index $d \in \{1,...,D\}$, evolutionary rates have the following form. The global birth rates are transformed via an exponential link function:

$$\lambda_d^- = \exp(\lambda_0^-)$$

$$\lambda_d^+ = \exp(\lambda_0^+)$$

The remaining transition rates are log-normally distributed:

$$\rho_d^{-+} \begin{cases} \sim \text{LogNormal}(\rho_0^{-+}, \sigma^{\rho^{-+}}) & \text{if } x_d \in \{\text{ABSENT}, -\text{IC}, +\text{IC}\} \\ = 0 & \text{otherwise} \end{cases}$$

$$\rho_d^{+-} \begin{cases} \sim \text{LogNormal}(\rho_0^{+-}, \sigma^{\rho^{+-}}) & \text{if } x_d \in \{\text{ABSENT}, \text{-IC}, \text{+IC}\} \\ = 0 & \text{otherwise} \end{cases}$$

$$\mu_d^- \sim \text{LogNormal}(\mu_0^-, \sigma^{\mu^-})$$

$$\mu_d^+ \sim \text{LogNormal}(\mu_0^+, \sigma^{\mu^+})$$

HalfNormal$(0,1)$ priors are placed over standard deviation parameters σ. Not all cognate classes attest all three states; some only express the pairs of states (ABS,$-$IC) and (ABS,$+$IC). These characters do not provide information that bears on transitions between the states $-$IC and $+$IC, but provide information regarding the birth rates and loss rates of cognate classes displaying these patterns. For characters of this sort, transitions to and from the unattested state are set to zero, as shown above.

The likelihood of each trait $P(x_d|\Psi, Q_d)$ was corrected for ascertainment bias. This correction is intended to account for the fact that the observed cognate classes represent only a fraction of the cognate classes that have existed during the course of each family's history, as many will have died out before being recorded [28, 26, 29, 30]. This amounts to conditioning the trait likelihood on the probability that the trait would be observed in the first place under the CTM process that governs its evolution. The corrected likelihood is equal to the following:

$$\frac{P(x_d|\Psi, Q_d)}{1 - P(x_{\text{ABS}}|\Psi, Q_d)}$$

Above, x_{ABS} represents a trait likelihood matrix with the value ABSENT for all tips in the phylogeny. For comparability between $P(x_d|\Psi, Q_d)$ and $P(x_{\text{ABS}}|\Psi, Q_d)$, x_{ABS} is augmented to contain a tip descending from a branch of infinitesimal length grafted to the MRCA of all languages where the cognate class is present, the value of which is equal to the reconstructed value. Code used in model inference is available here: `https://github.com/chundrac/idcc/blob/main/cognate_models/run_model.R`

1.1.4 Baselines for cognate class traits

In this paper, hypotheses regarding the nature of the evolution of sound patterns within lexical items are assessed by inspecting asymmetries between pairs of evolutionary rates. For instance, it may be the case that cognate classes are considerably more likely to be born with the value $-$IC than the value $+$IC; it could also be the case that changes affecting the form of cognate classes are more likely to bring about changes of the type $+\text{IC} \rightarrow -\text{IC}$ than changes of the type $-\text{IC} \rightarrow +\text{IC}$; it

could additionally be the case that cognate classes are more likely to die out when they have the value $+$IC than when they have the value $-$IC.

In a Bayesian framework, the strength of evidence in favor of an asymmetry can be assessed by determining whether the appropriate highest density interval (HDI) of the posterior distribution of ratios between the rates in question excludes the value corresponding to the null hypothesis that there is no difference in rates. Technically speaking, this value is 1, as a ratio of one indicates no difference between the rates under comparison. At the same time, it is possible that null models of lexical evolution could bring about asymmetries in rates, and that skews in rates represent the byproduct of chance occurrences. For instance, generating a word form containing adjacent identical consonants in a given language is highly dependent on the language's consonant inventory size — this would mean that asymmetries in birth rates of $\pm$IC should be compared to such a baseline. Additionally, the operation of sound changes which lack the specific goal of removing sequences of identical consonants may nonetheless remove sequences of identical consonants more frequently than it creates them — thus, asymmetries in mutation rates to $\pm$IC should be compared to a baseline representing this behavior. Loss rates of $\pm$IC are straightforwardly interpretable without needing an additional baseline of this sort. For this reason, I present additional baselines against which to compare posterior distributions of ratios between birth and mutation rates, which serve as a more conservative chance measure than the standard null value of 1 and give a clearer sense of the evolutionary pressure underlying these asymmetries.

1.1.4.1 Baseline birth rates of cognate class traits Under a process where sequences are generated by randomly sampling consonants from the uniform distribution, the probability of generating a sequence w containing at least two adjacent identical consonants is equal to the following, where $|w|$ denotes sequence length and $+\text{IC} \in w$ indicates the presence of adjacent identical consonants within a sequence:

$$P(+\text{IC} \in w) = \sum_{i=1}^{N} P(|w| = i) P(+\text{IC} \in w; |w| = i)$$

In a language with S segments, $P(+\text{IC} \in w; |w| = 2) = \frac{1}{S}$. Since the probability of generating a sequence containing at least two adjacent identical consonants is higher for longer sequences, $P(+\text{IC} \in w)$ will be higher when $P(|w| = i) = \frac{1}{N}$ for all $i \in \{1, ..., N\}$. Assuming that shorter sequences are more frequently generated than longer ones, we expect this quantity to approach $\frac{1}{S}$ as $P(|w| = 2)$ approaches 1, allowing us to derive a lower bound $P(+\text{IC} \in w) \geq \frac{1}{S}$. The expected ratio between words without and words with identical consonants under chance will then be less than or

equal to $S-1$. In the case of a hypothetical language requiring that a minimal word consist of more than two consonants, this ratio will be even smaller. Numbers of consonants for languages in each family (Afro-Asiatic was taken as a proxy for Semitic) were taken from the PHOIBLE database [31].

Concretely, if the HDI of the posterior distribution of ratios of birth rates of cognate classes with $-$IC versus $+$IC in a phylogeny is above 1, it means that cognate classes with $-$IC are born more frequently than cognate classes with $+$IC, but this may still be an artifact of consonant inventory size. However, if the HDI is greater than $S-1$ (derived from an inventory size representative of the phylogeny under analysis), it means that the frequency exceeds the maximum ratio expected under chance.

1.1.4.2 Baseline +IC → −IC vs. −IC → +IC mutation rates A simulation procedure was used to estimate the frequencies at which neutral models of sound change are expected to introduce sequences of identical consonants into lexical items versus remove them. Frequencies of such changes depend on existing frequencies of sound patterns found across the lexicon. To ensure that frequencies of word lists to which simulated sound changes were realistic, word lists from languages in each data set were used (simulations were applied to languages with 500 or more entries).

The sound changes employed consisted of 2002 individual diachronic changes or sets of synchronous changes extracted from a compendium of sound changes operating in the Indo-European, Semitic, and Uralic families [32], the most comprehensive collection of changes affecting consonants that also employs phylogenetic controls (e.g., by not counting a sound change multiple times if it occurs in languages deemed to be closely related). This phylogenetic control ensures that relative frequencies of sound changes within the data set are due to parallel occurrence of these changes and not one-off events in the shared history of multiple languages. These changes were restricted to include changes operating within conditioning environments general enough that they would be present in the data to which the changes were applied during the simulation process. To increase applicability, conditioning environments were further simplified: the environment _ was treated as UNCONDITIONED, i.e., affecting all segments of a particular type; the environment `#_` was treated as WORD-INITIAL; the environments `_V`, `C_`, `V_C`, `_C`, `V_`, and `V_V` were treated as WORD-MEDIAL; and the environments `V_#` and `_#` were treated as WORD-FINAL. Consonantal segments in each processed data set were identified with counterparts in the data set of sound changes, which employed a different notation.

Extracted changes have the format found in Table 2, as displayed for a handful of changes organized by change group number (an arbitrary number assigned to individual changes or groups

of simultaneously occurring changes).

Table SI 2: Selected sound changes extracted from [32].

Change group no.	Input	Output	Env. in source	Modified env.
2	f	v	#_	Word-initial
2	θ	ð	#_	Word-initial
2	s	z	#_	Word-initial
12	ʔ	β	V_V	Word-medial
12	θ	ð	V_V	Word-medial
12	x	ɣ	V_V	Word-medial
773	s	r	V_#	Word-final

For each language in each data set, a sound change or set of synchronous changes that was capable of operating in the language in question was sampled at random and applied to the language's word list, substituting the output segment for the input segment in the relevant environment. For the Semitic and Uralic data sets, changes were applied to etymologically aligned subword strings after processing subword strings to detect whether subword boundaries coincided with word boundaries. For the Austronesian data set, sound changes were applied to whole word strings, and the output of this process was subjected to the longest substring extraction procedure described above.

Subsequently, the number of $+\text{IC} \rightarrow -\text{IC}$ vs. $-\text{IC} \rightarrow +\text{IC}$ changes in the relevant subword unit were tabulated, and a ratio computed by dividing the former number by the latter number (with a smoothing constant of 1 added to each number to prevent zero division). This procedure was carried out 500 times per language, with ratios averaged at the language level.

Code used to simulate sound changes for cognate class data sets is found here: `https://github.com/chundrac/idcc/blob/main/ACD_processing/process_data/simulate_sound_change.py`, `https://github.com/chundrac/idcc/blob/main/SED_processing/process_data/simulate_sound_change.py`, `https://github.com/chundrac/idcc/blob/main/Uralonet_processing/process_data/simulate_sound_change.py`

1.2 Cognate-concept traits

The evolution of cognate-concept (alternatively root-meaning) traits [33, 34, 30] was analyzed using data from a subset of the Lexibank repository [35] that has been further processed to normalize orthographic forms as well as link forms in different languages to the Concepticon semantic taxonomy [36]. I used data sets for which cognacy was coded and for which reliable phylogenetic

tree samples have been published. Data from five families were analyzed. These were Dravidian [37, 38, 39], Indo-European [40, 41, 30], Sino-Tibetan [42, 43], Turkic [44, 45], and Uto-Aztecan [46]. Published tree samples used are found in Figures 8–12. Geographic distributions of families are given in Figure 1.2.2.

1.2.1 Data processing

Data processing was less involved that for cognate class traits given the comparable nature of the data sets under analysis. Forms in different languages were automatically coded according to whether or not they contained a sequence of identical consonants separated by a single vowel within morpheme boundaries (demarcated by the symbol $+$). This was relatively straightforward due to the space-delimited orthographic normalization of forms. The Cross-Linguistic Transcription Systems (CLTS) database [47] was used to determine which segments in each string were consonants. The geminate marker ː was stripped from geminate segments and sequences of identical segments were simplified to one segment before a script was used to detect the presence of adjacent identical consonants within morphological boundaries (represented by the character $+$).

1.2.2 Preparation for phylogenetic analysis

A language expresses a given semantic concept using formal material corresponding to one or more cognate classes, in which sequences of identical consonants can be present or absent. For instance, Portuguese expresses the concept DRINK with the form /bɨbeɾ/, which contains identical consonants and is a reflex of the Proto-Indo-European etymon *peh₃-. Thus, for each language in a family, cognate-concept traits are coded according to the states $\{$ABSENT,$-$IC,$+$IC$\}$.

Cognate-concept characters for different families were transformed into binarized likelihood matrices. In the case of lexical polymorphism (i.e., in which a language attests multiple forms for a meaning), multiple likelihoods were set to one. Analyses were restricted to data corresponding to 100 basic concepts [48] available through Concepticon [36]. Concept rankings were taken from NorthEuraLex [49]. The Dravidian data set consisted of 709 concept-cognate traits corresponding to 93 concepts from 20 languages. The Indo-European data set consisted of 686 concept-cognate traits corresponding to 96 concepts from 19 languages. The Sino-Tibetan data set consisted of 1517 concept-cognate traits corresponding to 83 concepts from 44 languages. The Turkic data set consisted of 225 concept-cognate traits corresponding to 90 concepts from 31 languages. The Uto-Aztecan data set consisted of 1087 concept-cognate traits corresponding to 92 concepts from 33 languages. Code used to process and prepare data for phylogenetic analysis is found here: `https://github.com/chundrac/idcc/blob/main/cognate_concept_models/process_data.R`

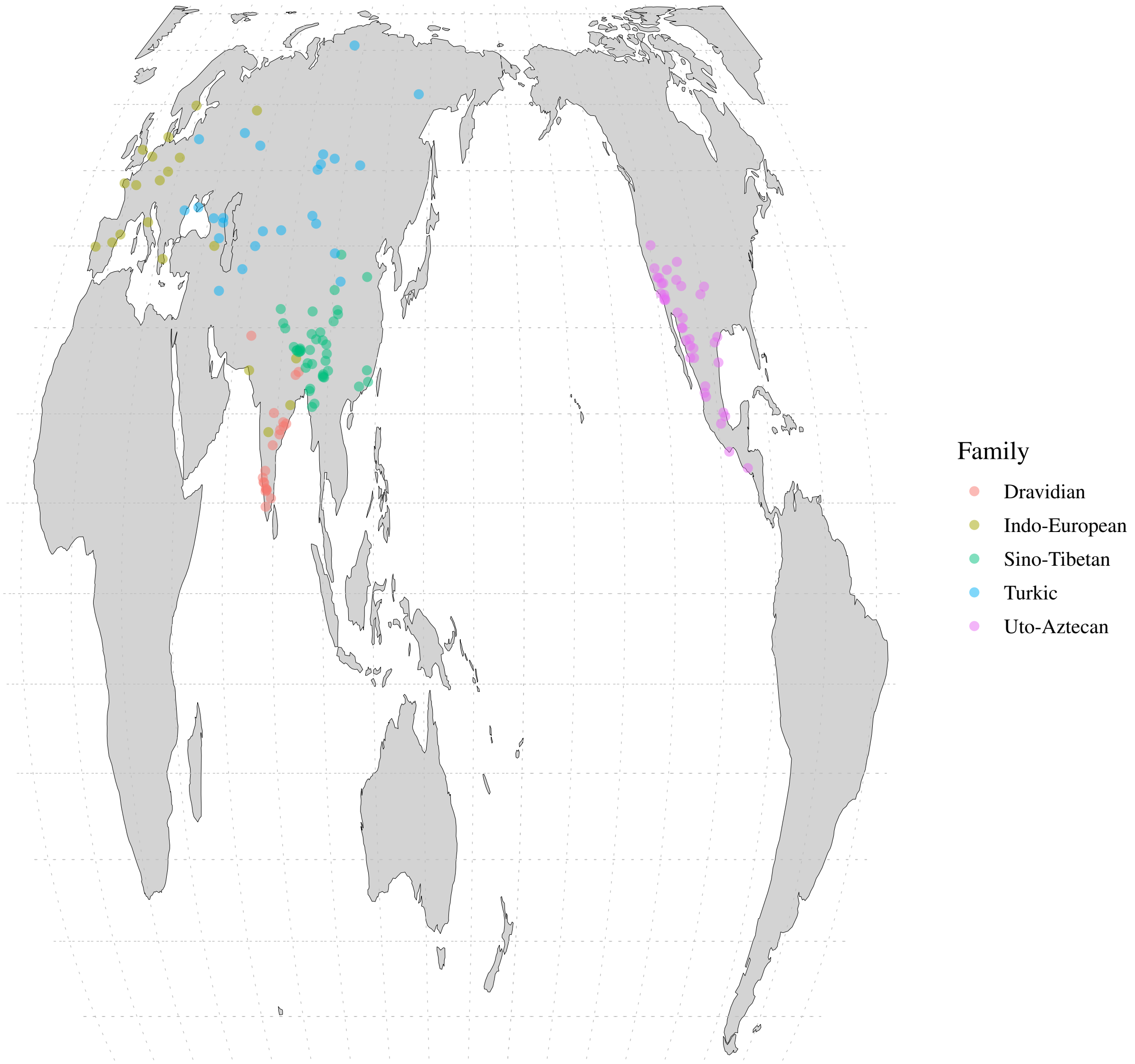

Figure SI 7: Geographic locations of languages used in analyses of cognate-concept traits, for languages with geographic coordinates in the Glottolog [21] database.

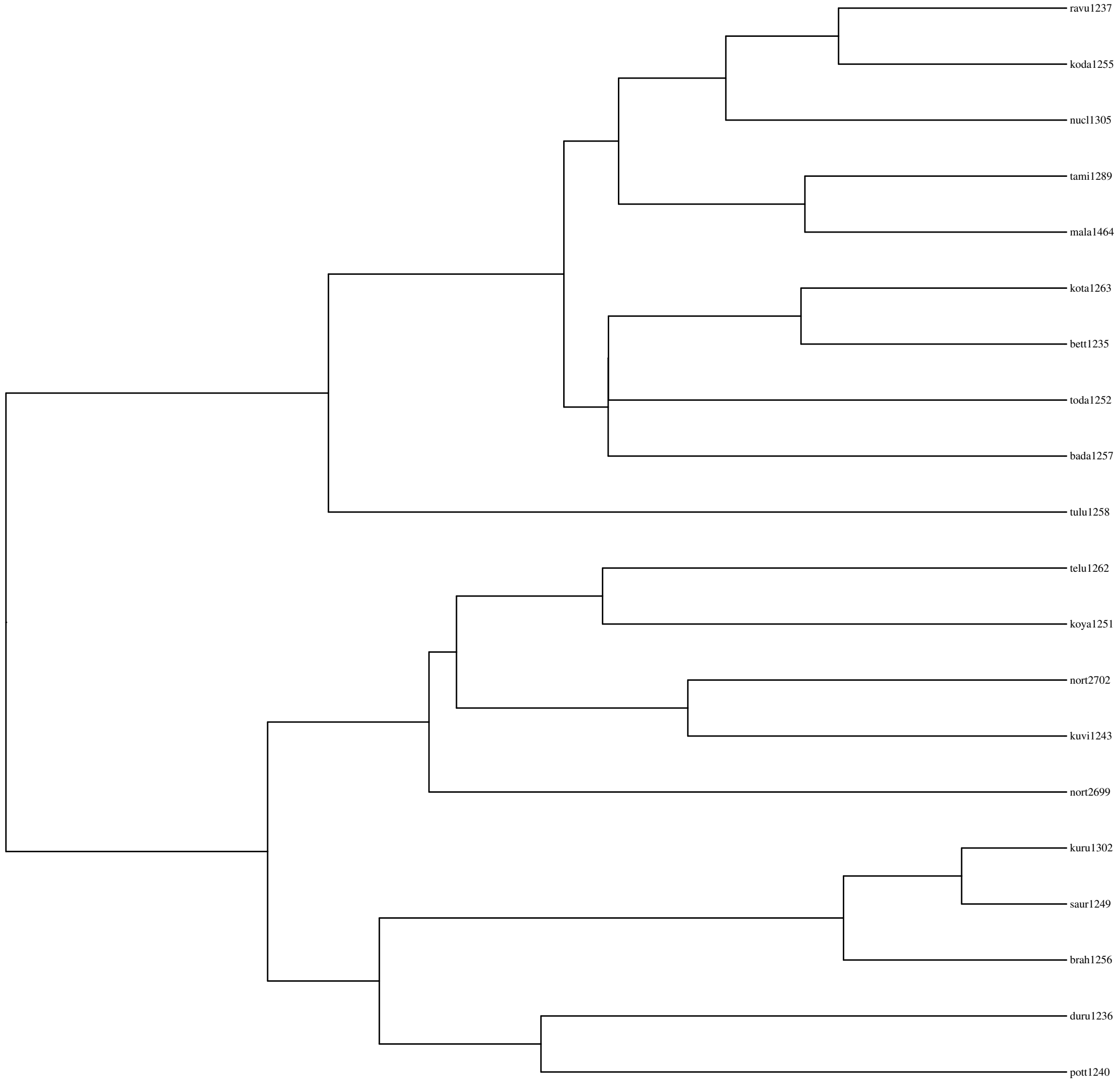

Figure SI 8: Dravidian MCC tree [39], with languages retained for study.

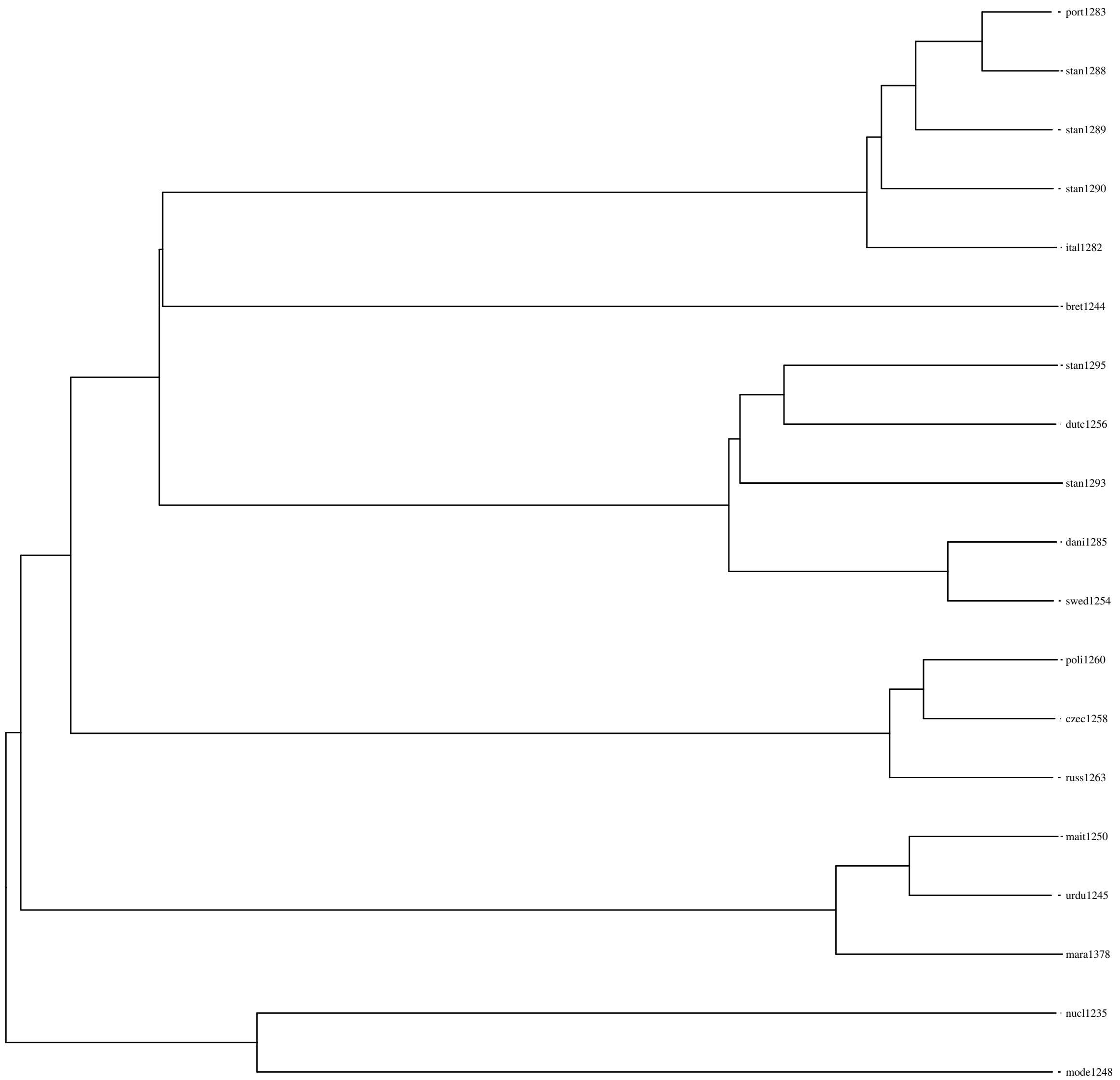

Figure SI 9: Indo-European MCC tree [30], with languages retained for study.

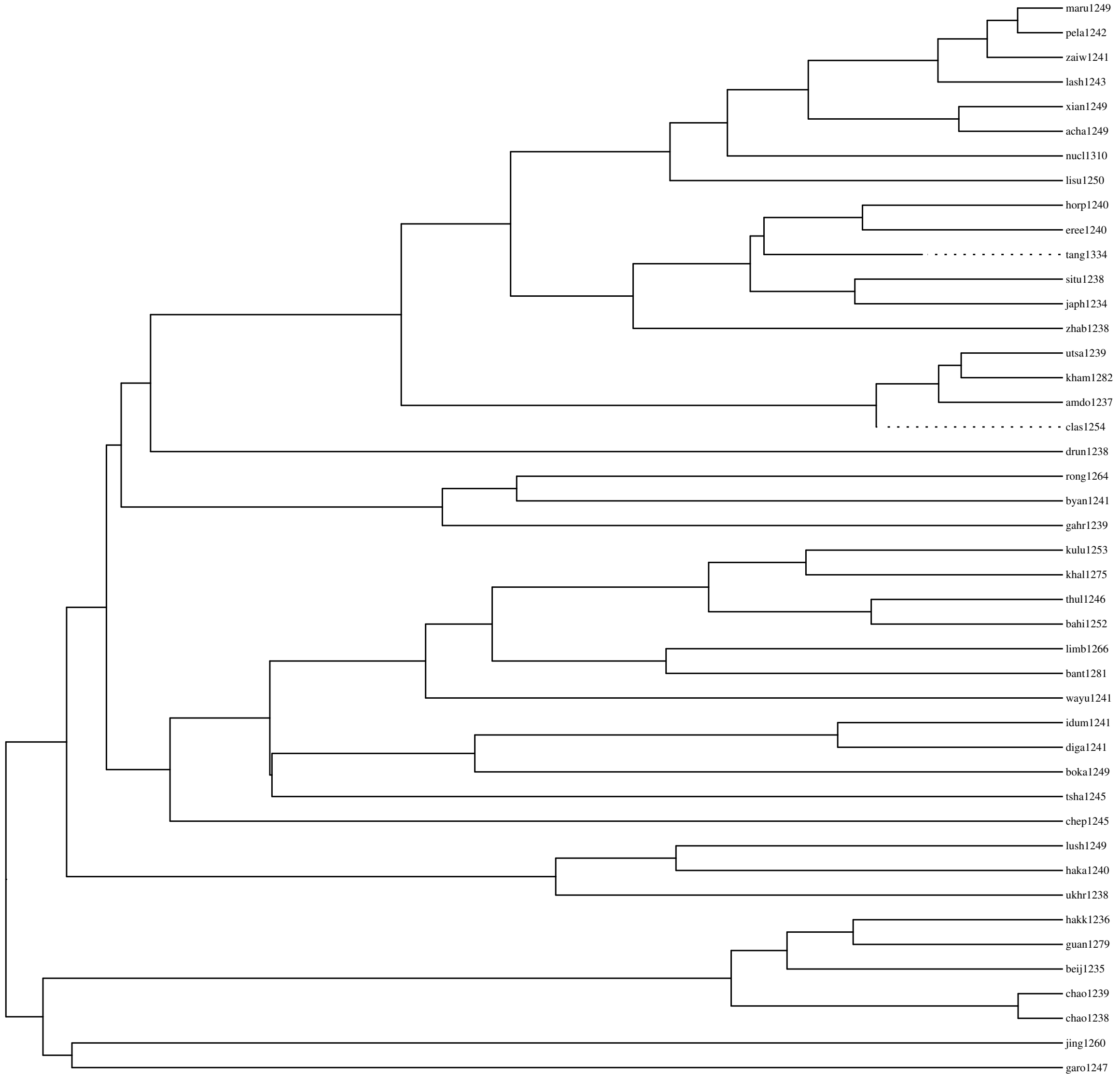

Figure SI 10: Sino-Tibetan MCC tree [43], with languages retained for study.

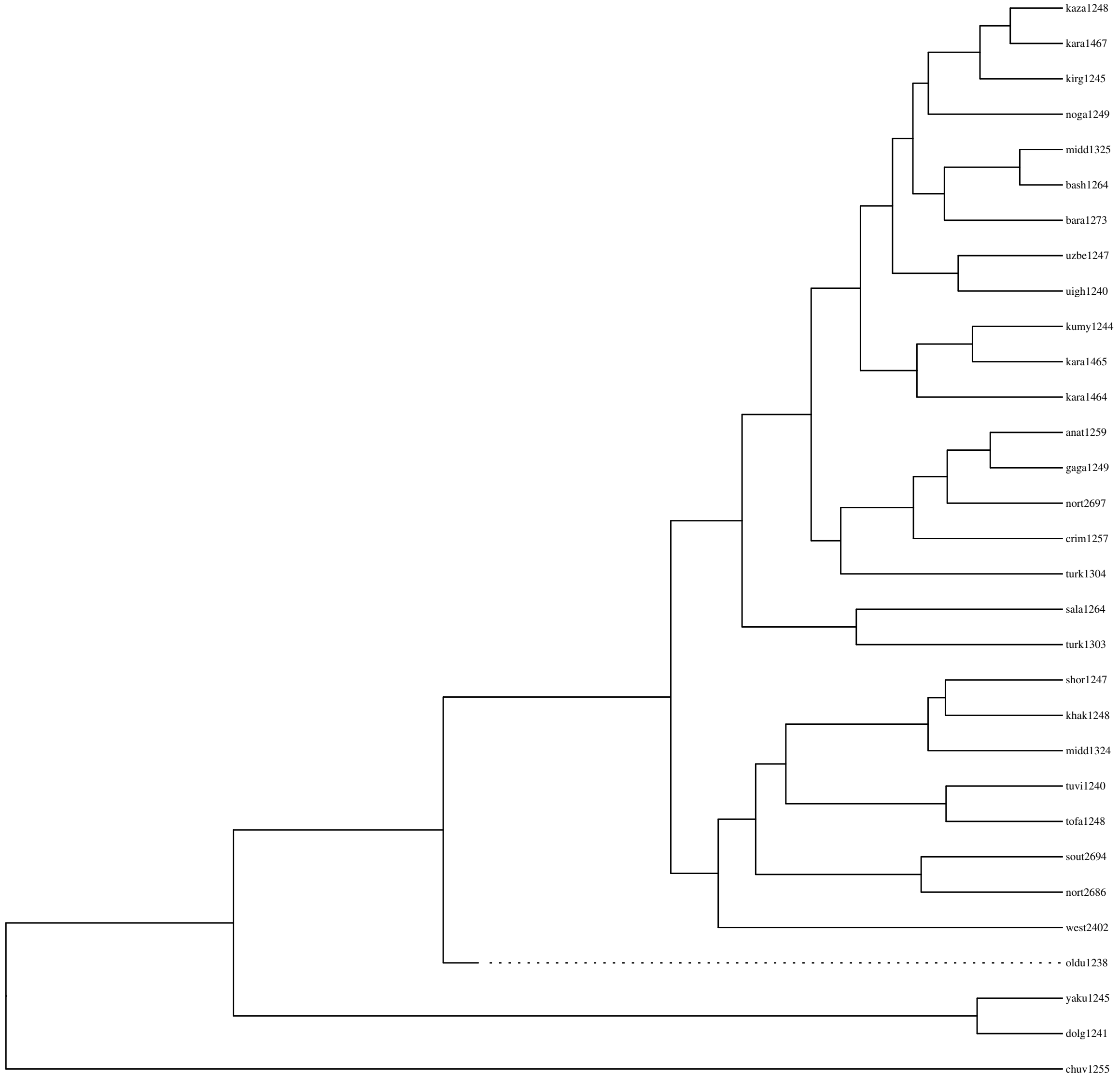

Figure SI 11: Turkic MCC tree [44], with languages retained for study.

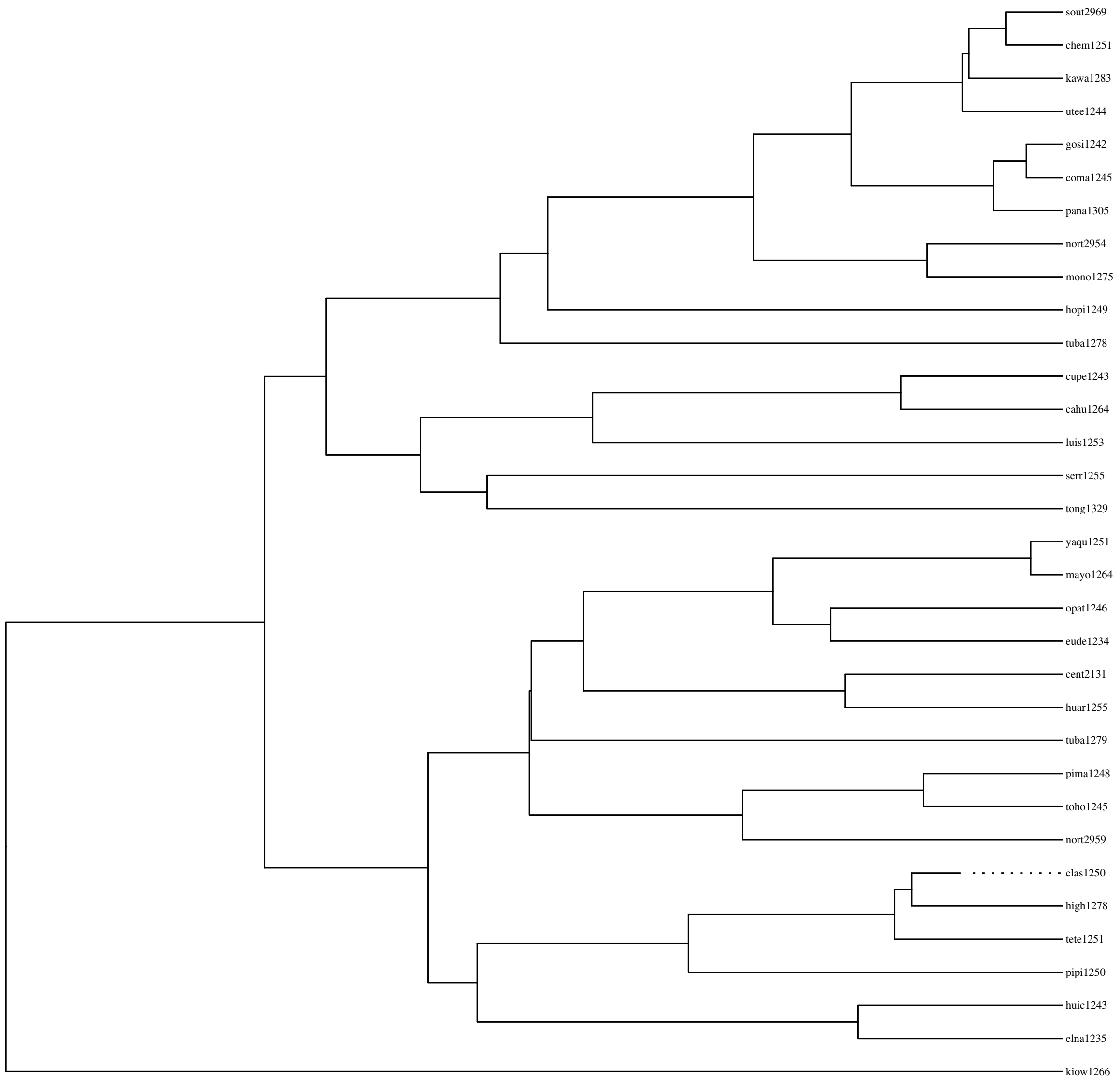

Figure SI 12: Uto-Aztecan MCC tree [46], with languages retained for study.

1.2.3 Phylogenetic analysis of cognate-concept traits

Cognate-concept traits were modeled as evolving according to a CTM process. Since they are homoplastic (i.e., a cognate class can come to express the same meaning independently on two different lineages), standard models used to analyze morphologically dependent traits are applicable without the need to account for the single-birth criterion.

As above, hierarchical models were used to jointly analyze the evolution of cognate-concept traits jointly within separate families. Transition rates were assumed to vary at the concept level; the likelihood for a given cognate-concept trait with index $d \in \{1,...,D\}$ under a phylogeny Ψ, $P(x_d|\Psi, Q_{\text{concept}[d]})$ depends on the transition rates for the concept which the trait expresses and can be computed using the pruning algorithm.

For each concept $c \in \{1,...,C\}$, transitions between the states $\{$ABSENT, $-$IC, $+$IC$\}$ take place according to the following rate matrix:

$$Q_c = \begin{pmatrix} \text{—} & \lambda_c^- & \lambda_c^+ \\ \mu_c^- & \text{—} & \rho_c^{-+} \\ \mu_c^+ & \rho_c^{+-} & \text{—} \end{pmatrix}$$

Here, all rates (including the birth rates λ^- and λ^+) vary across concepts, since cognate-concept traits are homoplastic, and concept-cognate traits for certain concepts may arise more frequently than for others.

Priors over the parameters λ_0^-, λ_0^+, ρ_0^{-+}, ρ_0^{+-}, μ_0^-, μ_0^+, which represent log baseline rates, follow the standard normal distribution. For a given concept with index $c \in \{1,...,C\}$, evolutionary rates are distributed as follows:

$\lambda_c^- \sim \text{LogNormal}(\lambda_0^-, \sigma^{\lambda^-})$

$\lambda_c^+ \sim \text{LogNormal}(\lambda_0^+, \sigma^{\lambda^+})$

$\rho_c^{-+} \sim \text{LogNormal}(\rho_0^{-+}, \sigma^{\rho^{-+}})$

$\rho_c^{+-} \sim \text{LogNormal}(\rho_0^{+-}, \sigma^{\rho^{+-}})$

$\mu_c^- \sim \text{LogNormal}(\mu_0^-, \sigma^{\mu^-})$

$\mu_c^+ \sim \text{LogNormal}(\mu_0^+, \sigma^{\mu^+})$

HalfNormal$(0,1)$ priors are placed over standard deviation parameters σ. The rate parameters for concept-cognate trait $d \in \{1,...,D\}$ are equal to the rate parameters for concept$[d]$, if x_d attests all three states $\{$ABSENT,$-$IC,$+$IC$\}$; otherwise, ρ_d^{-+} and ρ_d^{+-} are set to zero, as in the previous study.

Trait likelihoods were corrected for ascertainment bias in the manner described above. Here, x_{ABS} represents a trait likelihood matrix with the value ABSENT for all tips in the phylogeny. Code used in model inference is available here: `https://github.com/chundrac/idcc/blob/main/cognate_concept_models/run_model.R`

1.2.4 Baselines for cognate-concept traits

1.2.4.1 Baseline birth rates of cognate class traits Under a null model in which basic vocabulary items are sampled from the general (i.e., basic and nonbasic) vocabulary at random, with no sensitivity to the sound patterns displayed by individual forms, the ratio of birth rates of cognate-concept traits without versus with sequences of identical consonants should be comparable to the ratio between forms without and with identical consonants in the lexicon from which basic vocabulary items are sampled.

These ratios are estimated for languages in each family under study on the basis of large word lists comprising basic as well as non-basic items. Dravidian, Indo-European and Turkic ratios were estimated from NorthEuraLex [49]. Sino-Tibetan ratios were estimated from the Sino-Tibetan Etymological Dictionary and Thesaurus [50]. Uto-Aztecan ratios were estimated from available digitized resources for Nahuatl [51], Yaqui [52] and the Bridgeport dialect of Northern Paiute [53]. For each language, the number of forms lacking sequences of identical consonants was divided by the number of forms containing sequences of identical consonants.

1.2.4.2 Baseline $+$IC $\rightarrow$ $-$IC vs. $-$IC $\rightarrow$ $+$IC mutation rates This simulation procedure was carried out as described for cognate class traits, with the difference that sound changes were applied only to the 100 basic vocabulary items under analysis rather than larger word lists.

1.3 Inference

Data were processed using Python 3 as well as version 0.6-99 of the R package phytools [54]. Models were fitted using RStan version 2.26.13 [55], running the No U-Turn Sampler (NUTS) over 4 chains for 2000 iterations, with the first half discarded as burn-in. The Stan function `map_rect` was used in order to enable multi-threading, as parallelization speeds up inference considerably. The argument `adapt_delta` of the `stan` function was set to 0.99 in order to reduce the number of divergent transitions after warmup, which can lead to convergence problems. Model convergence was assessed via the potential scale reduction factor [56], with values under 1.1 taken to indicate convergence. To incorporate phylogenetic uncertainty, the model was run on 25 trees from each tree sample and the resulting posterior samples for runs that reached convergence were concatenated

Table SI 3: Interpretation of parameters used in analyses of cognate class and cognate-concept traits, along with the research questions they are used to address as well as answers. Subscript zeros indicate that parameters represent log mean rates around which rates for individual traits are log-normally distributed (with the exception of $\lambda_0^{\pm}$ for cognate class traits; see text). Each hypothesis is assessed by computing the ratio between rates 1 and 2 after exponentiating them.

Cognate class traits

Rate 1	Rate 2	Question addressed
λ_0^-<br>(birth rate, $-$IC)	λ_0^+<br>(birth rate, $+$IC)	Do word forms without IC arise more frequently than forms with IC?<br>(Yes, more frequently than at chance)
ρ_0^{+-}<br>(mut. rate, $+$IC$\rightarrow$ $-$IC)	ρ_0^{-+}<br>(mut. rate, $+$IC$\rightarrow$ $-$IC)	Are $+$IC$\rightarrow$ $-$IC changes more frequent than $-$IC$\rightarrow$ $+$IC changes?<br>(Yes, but not more frequently than at chance)
μ_0^+<br>(loss rate, $+$IC)	μ_0^-<br>(loss rate, $-$IC)	Are forms with $+$IC more likely to die out than forms with $-$IC?<br>(No)

Cognate-concept traits

Rate 1	Rate 2	Question addressed
λ_0^-<br>(birth rate, $-$IC)	λ_0^+<br>(birth rate, $+$IC)	Do word forms without IC enter the basic vocabulary more frequently than forms with IC?<br>(Yes, but not more frequently than chance in 4/5 families)
ρ_0^{+-}<br>(mut. rate, $+$IC$\rightarrow$ $-$IC)	ρ_0^{-+}<br>(mut. rate, $+$IC$\rightarrow$ $-$IC)	Are $+$IC$\rightarrow$ $-$IC changes more frequent than $-$IC$\rightarrow$ $+$IC changes in basic vocabulary items?<br>(Greater than chance in only 1/5 families)
μ_0^+<br>(loss rate, $+$IC)	μ_0^-<br>(loss rate, $-$IC)	Are forms with $+$IC phased out of basic meaning functions more often than forms with $-$IC?<br>(Yes)

together, yielding a maximum of 100000 samples per model. 95% HDIs were computed using the R package HDInterval [57].

2 Results

2.1 Cognate class traits

Bayesian phylogenetic models were used to disentangle the mechanisms that shape the evolutionary trajectories of individual cognate classes (e.g., forms descending from Proto-Malayo-Polynesian *dapdap) in three families (Austronesian, Semitic, and Uralic). Over the course of a language family's phylogenetic history, ancestral word forms are born, undergo processes of word form mutation and differentiation (as the speech varieties in which they exist diversify phylogenetically), and die out on different phylogenetic lineages. Analyses of the evolution of morpheme-internal identical consonants within cognate class traits in three language families were carried out using a

hierarchical phylogenetic model containing six parameters of interest (schematized in Figure 1 and further defined in Table 3): λ_0^-, the log birth rate of forms without identical consonants; λ_0^+, the log birth rate of forms with identical consonants; ρ_0^{-+}, the log mean rate at which sequences of identical consonants arise within forms; ρ_0^{+-}, the log mean rate at which sequences of identical consonants are lost within forms; μ_0^-, the log mean loss rate of forms without identical consonants; and μ_0^+, the log mean loss rate of forms with identical consonants. The hierarchical model used allows parameters to vary at the level of individual cognate classes, which undergo change according to evolutionary rates that are log-normally distributed around the mean parameters ρ_0^{-+}, ρ_0^{+-}, μ_0^-, μ_0^+, or in the case of birth rates, according to which all cognate classes arise and which are shared across all cognate classes, set to $\exp\left(\lambda_0^-\right)$ and $\exp\left(\lambda_0^+\right)$. Parameters that vary at the level of individual cognate classes are analogous to random effects in mixed-effects regression models, in that they account for individual cognate-level idiosyncrasies, while the mean parameters listed above are comparable to fixed effects, as they capture global trends in the evolutionary system. Pairwise comparisons between parameters allow us to assess whether forms with and without identical consonants are born at different rates (λ_0^+ vs. λ_0^-), whether identical consonants are gained and lost within forms at different rates (ρ_0^{-+} vs. ρ_0^{+-}), and whether forms with and without identical consonants are lost at different rates (μ_0^+ vs. μ_0^-). Strengths of differences in rates were quantified by taking the ratio of the two mean rates in question, i.e., by inspecting the posterior distributions of the quantities $\exp\left(\lambda_0^- - \lambda_0^+\right)$, $\exp\left(\rho_0^{+-} - \rho_0^{-+}\right)$, and $\exp\left(\mu_0^+ - \mu_0^-\right)$. Evidence for a difference is taken to be decisive if the 95% highest density interval of ratios does not contain values representing the null hypothesis [58]. A standard null value is 1: ratios greater than 1 indicate that one change type is more frequent than another. However, in some cases, skewed distributions are expected even under null models of language generation [59]. For this reason, posterior ratios are also compared to quantities representing baseline asymmetries in frequencies of change types that would be expected under neutral processes of language evolution.

Figure 13 shows posterior distributions of ratios of interest. Distributions are annotated with the percentage of posterior samples for which the ratio is greater than one (represented by dashed lines). Distributions of ratios pertaining to birth rates and mutation rates are also annotated with values representing ranges of ratios (and median values thereof) that would be expected under neutral models of language change. These quantities are estimated from data from each family under analysis, assuming that distributions of features found in contemporary languages are representative of those encountered during the history of the language family to which they belong [60]. Under a neutral process in which words are generated by randomly sampling segments with uniform probabilities, the ratio of words born without versus with sequences of identical consonants is

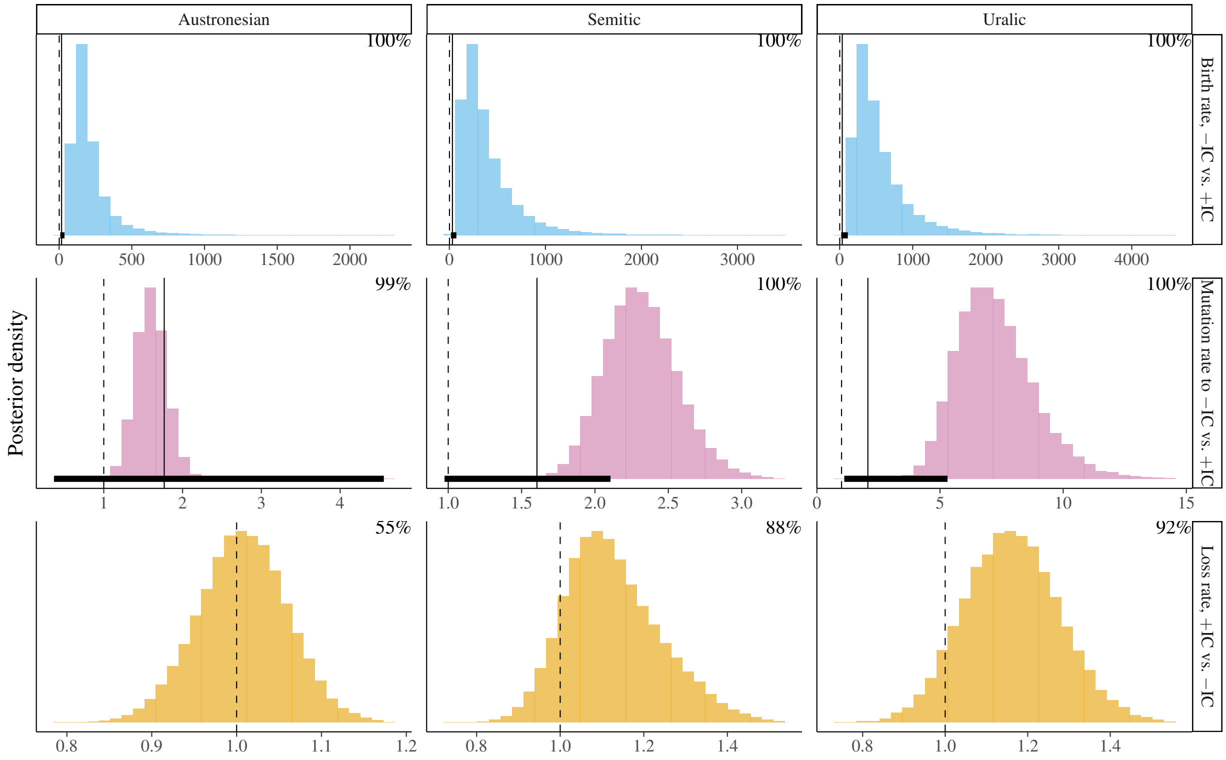

Figure SI 13: Histograms from analyses of cognate traits displaying posterior distributions of ratios of parameters of interest for different families: birth rate of words with value −IC (no identical consonants) vs. +IC (with identical consonants; top), rate of +IC → −IC vs. −IC → +IC change (middle), and loss rate of words with +IC vs. −IC (bottom). Histograms are annotated with percentages of samples for which ratios are greater than 1 (given by vertical dashed lines). Solid black vertical lines in upper two rows represent median baseline quantities; horizontal lines represent ranges of baseline quantities.

no greater than the number of consonants in a language's segmental inventory, minus one. This quantity is provided for languages in each family for which such data are available. Baseline values for ratios between mutations that remove versus introduce sequences of identical consonants are estimated by simulating the effects of neutral models of sound change [61, 62] using word lists of languages in the families under study. For loss rates, a baseline value of 1 is sufficient for the purpose of interpreting posterior ratios.

Across all three families, there is decisive evidence that forms without identical consonants are born more frequently than those with identical consonants (median: 171.49, 95% HDI: [51.67, 526.09]; 303.07, [53.17, 1017.29]; and 428.73, [89.93, 1365.98] times more frequently in Austronesian, Semitic, and Uralic, respectively). Additionally, there is decisive evidence that these

ratios are greater than would be expected under a chance baseline based on sizes of segmental inventories, as posterior HDIs are consistently greater in value than ranges of baselines expected under a neutral process of word generation (median: 17, total range: [8, 37]; 32, [16, 73]; and 35, [21, 112] times more frequently in Austronesian, Semitic, and Uralic, respectively). Mutational changes to forms that remove sequences of identical consonants are decisively more frequent than mutations that introduce them, although the ratios between transition rates pertaining to these changes are far lower than asymmetries in birth rates of forms with and without identical consonants (1.6, [1.23, 1.99]; 2.30, [1.85, 2.82]; and 7.09, [4.44, 10.45] in Austronesian, Semitic, and Uralic, respectively). 95% HDIs overlap with ranges of ratios expected under neutral models of sound change in all three families (1.76, [0.36, 4.55]; 1.60, [0.97, 2.10]; and 2.06, [1.11, 5.30] more frequently in Austronesian, Semitic, and Uralic, respectively), indicating that these ratios do not clearly exceed what is expected at chance levels. Posterior distributions do not support the idea that forms with sequences of identical consonants die out more frequently than those without them (1.01, [0.9, 1.11]; 1.11, [0.92, 1.37]; and 1.16, [0.94, 1.39] in Austronesian, Semitic, and Uralic, respectively.

These results indicate that asymmetries in birth rates of words play a major and consistent role in the under-representation of sequences of identical consonants in word forms, and to a weaker extent processes that mutate word forms, though this latter effect is not found in all families studied when interpreted according to a principled, conservative baseline. Crucially, however, word forms containing such sequences are no more likely to fall entirely out of use than those without: they exhibit as much longevity as their counterparts that do not contain identical consonants, though it is not clear from these results whether they survive in more marginal functions and restricted distributions.

2.2 Cognate-concept traits

A related set of phylogenetic models were used to analyze the evolution of morpheme-internal sequences of identical consonants within cognate-concept traits in five language families (Dravidian, Indo-European, Sino-Tibetan, Turkic, and Uto-Aztecan). These analyses shed light on the conditions under which cognate word forms enter and fall out of use in basic meaning functions, and the nature of the processes affecting word forms during the time in which they occupy such roles. Analyses focused on cognate-concept traits pertaining to one hundred concepts representing basic vocabulary items, chosen to maximize comparability of results across families [48]. Parameters of interest have similar interpretations as for the models described in the previous section (see Figure 1, Table 3). As above, posterior parameter values were compared to assess whether word forms without identical

consonants enter basic vocabulary meaning functions more frequently than those without (λ_0^- vs. λ_0^+), whether identical consonants are lost within forms used in the basic vocabulary more frequently than they are gained (ρ_0^{+-} vs. ρ_0^{-+}), and whether forms containing identical consonants are removed from the basic vocabulary more frequently than those without (μ_0^+ vs. μ_0^-). The baselines against which ratios for birth and mutation rates are compared differ from those employed for cognate class traits. Ratios of birth rates (i.e., between the rates at which forms without and with identical consonants enter languages' basic vocabulary) are compared to ratios between numbers of forms without versus containing identical consonants in contemporary languages' basic and non-basic vocabularies; this comparison tells us whether forms with identical consonants enter the basic vocabulary at a rate lower than would be expected from a neutral process in which basic vocabulary items are sampled randomly from the lexicon of a language. Ratios between mutation rates are compared to baselines generated via simulations of neutral sound change, as for cognate class traits, but restricted to forms expressing the one hundred concepts under analysis. As with cognate class traits, ratios between rates at which forms with and without identical consonants are removed from the basic vocabulary do not require interpretation against a baseline other than the standard null value of 1.

Figure 14 shows posterior distributions of ratios of interest. Distributions are annotated as in Figure 13. All families show decisive evidence that forms without identical consonants enter the basic vocabulary more frequently than forms with identical consonants (Dravidian: 17.85, [6.03, 35.98]; Indo-European: 15.85, [7.36, 29.69]; Sino-Tibetan: 26.56, [13.33, 46.81]; Turkic: 11.59, [3.41, 27.09]; Uto-Aztecan: 21.95, [9.47, 47.15]); however, these distributions overlap with ranges of ratios expected under a random sampling process from the lexicon in all families (Dravidian: 28, [16.5, 59]; Indo-European: 25.3, [7.78, 46.2]; Sino-Tibetan: 23.0, [5.56, 73]; Turkic: 31.1, [20.6, 34.2]; Uto-Aztecan: 7.89, [7.42, 9.46]) except for Uto-Aztecan, where usable digitized word lists comprising basic and non-basic vocabulary items were available for only three languages.

Indo-European is the only family exhibiting decisive evidence that mutational processes remove sequences of identical consonants from basic vocabulary items more frequently than they introduce them (Dravidian: 1.77, [0.18, 4.81]; Indo-European: 7.14, [1.55, 15]; Sino-Tibetan: 5.66, [0.95, 11.81]; Turkic: 1.38, [0.17, 3.76]; Uto-Aztecan: 1.7, [0.25, 3.67]); for Sino-Tibetan, the less conservative 89% HDI ([1.24, 10.18]) does not overlap with one. Indo-European posterior ratios do not overlap with ranges that would be expected under neutral processes of sound change affecting the basic vocabulary (Dravidian: 1.02, [0.94, 1.10]; Indo-European: 1.01, [0.92, 1.19]; Sino-Tibetan: 1.00, [0.97, 1.28]; Turkic: 1.05, [0.97, 1.16]; Uto-Aztecan: 1.09, [0.94, 1.42]), but there is considerable overlap for other families.

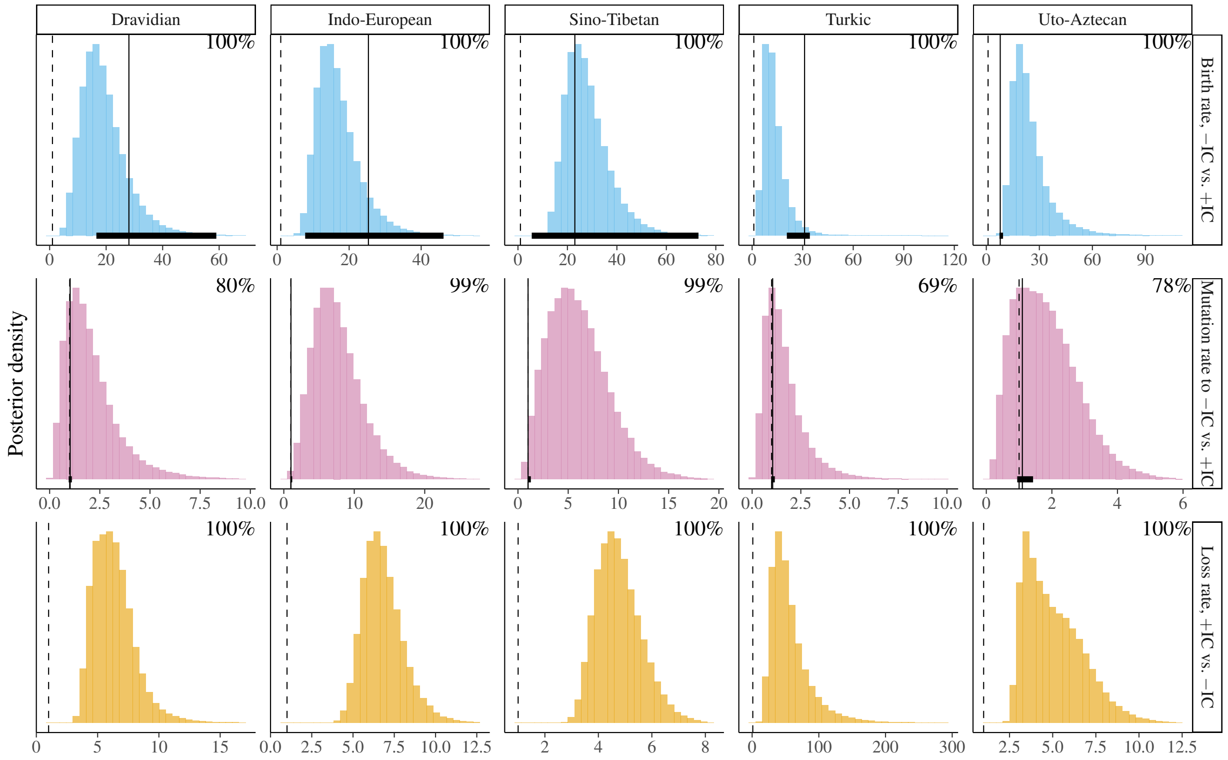

Figure SI 14: Histograms from analyses of cognate-concept traits displaying posterior distributions of ratios of parameters of interest for different families: birth rate of cognate-concept traits with −IC vs. +IC (top), rate of +IC → −IC vs. −IC → +IC change (middle) within cognate-concept traits, and loss rate of cognate-concept traits with +IC vs. −IC (bottom). Histograms are annotated with percentages of samples for which ratios are greater than 1 (given by vertical dashed lines). Solid black vertical lines in upper two rows represent median baseline quantities; horizontal lines represent ranges of baseline quantities.

All families show decisive support for the idea that cognate-concept traits are lost more frequently when the form expressing the concept in question contains identical consonants than when it does not (Dravidian: 6.11, [3.59, 9.59]; Indo-European: 6.67, [4.65, 9.36]; Sino-Tibetan: 4.68, [3.27, 6.44]; Turkic: 49, [16.56, 118.88]; Uto-Aztecan: 4.77, [2.65, 8.34]). This indicates that while word forms with identical consonants do not exhibit less overall longevity than word forms without identical consonants, they are phased out of basic meaning functions more frequently than those without.

2.2.1 Inspection of concept-level rates

The rates reported above characterize the dynamics of lexical replacement within the basic vocabulary (as represented by the 100 word list) as a whole. Variation among rates was inspected at the concept level, to investigate whether asymmetries of the sort described above are found for all concepts, and whether relative strengths of these asymmetries differ across concepts. 95% HDIs for asymmetries in birth rates of cognate-concept traits contain values greater than 1 in most concepts in most families, with the exception of Turkic (Dravidian: 93 out of 93 traits, Indo-European: 96/96, Sino-Tibetan: 83/83, Turkic: 43/90, Uto-Aztecan: 92/92; Figures 16–20). 95% HDIs for asymmetries in mutation rates which remove versus introduce sequences of identical concepts within cognate-concept traits do not display the same behavior, with relatively few 95% HDIs excluding values less than 1 (Dravidian: 0/93, Indo-European: 11/96, Sino-Tibetan: 6/83, Turkic: 0/90, Uto-Aztecan: 0/92; Figures 21–25) even in the Indo-European and Sino-Tibetan families, where asymmetries in mean rates are decisively greater than 1. Asymmetries in concept-level loss rates have HDIs including only values greater than 1 for most concepts in most families (Dravidian: 88/93, Indo-European: 94/96, Sino-Tibetan: 82/83, Turkic: 83/90, Uto-Aztecan: 91/92; Figures 26–30). In sum, birth and loss rates indicate a decisive preference in favor of forms without identical consonants in most concepts.

Visual inspection (Figures 16–30) shows that distributions of asymmetries in concept-level loss rates exhibit more variation and less tendency to overlap than concept-level birth rates or concept-level mutation rates. I quantify pairwise comparisons in asymmetry strength between concepts using a method proposed for hierarchical models, which involves computing the percentage of samples for which an asymmetry is greater in one concept than another, with evidence for a contrast taken to be decisive for percentages of 95% or more [63]. Few comparisons for birth rates exhibit decisive evidence for a difference (Dravidian: 0 out of 4278 pairwise comparisons; Indo-European: 0/4560; Sino-Tibetan: 1/3403; Turkic: 83/4005; Uto-Aztecan: 0/4186), along with mutation rates (Dravidian: 0/4278; Indo-European: 30/4560; Sino-Tibetan: 48/3403; Turkic: 2/4005; Uto-Aztecan:

6/4186). Loss rates exhibit a higher number of decisive contrasts (Dravidian: 1469/4278; Indo-European: 2122/4560; Sino-Tibetan: 2038/3403; Turkic: 666/4005; Uto-Aztecan: 2132/4186), indicating that while loss rates tend on the whole to favor items without identical consonants, the strength of this preference differs considerably across concepts.

A question that arises is whether the frequency with which words containing identical consonants are replaced in specific meaning functions is related to the need probability of concepts [64], which can generally be estimated via the frequencies with which concepts occur in corpora. Operationalizing concept-level need probability in a manner that is applicable to all languages of the five phylogenies analyzed here is challenging, since reliable corpus resources are not available for all languages in our sample. Furthermore, need probabilities are dynamic and evolve over time, making it difficult to capture all evolutionary aspects of this attribute with a static measure. Here, I use two means of operationalizing concept-level need probability.

The first of these is the BASICNESS score [65] of the NorthEuraLex database v. 0.9 [49], which combines a cross-linguistically applicable form simplicity metric with a measure of stability based on cross-linguistic distances between forms (represented by the variable `position_in_ranking`). This metric consists of a single cross-linguistic rank value for each concept analyzed in this paper. Basicness and stability correlate with frequency [66], so this metric approaches frequency only indirectly. At the same time, the form simplicity metric is based on average information content, which is closely related to word length. This may introduce some confounds, since shorter words are less likely to contain sequences of identical consonants than longer ones, and if longer words are more likely to be replaced in certain meaning functions than shorter ones [67, 68], there is a higher likelihood that words containing identical consonants will be replaced than those without.

As a cross check, I use cross-linguistic frequency values of Swadesh list basic vocabulary items collected by [69], available via the Concepticon [36]. This data set consists of log frequencies for 200 basic vocabulary items in up to 17 different languages, of which 9 are Indo-European languages originating in Europe. To reduce the dimensionality of this data set, concept-level log frequencies across languages were submitted to principle component analysis using the R function `prcomp`. Following the procedure employed by the authors of the initial study, missing concept frequencies in a given language are replaced with the mean frequency in all languages for which concept frequencies are available. Of the resulting components, PC1 explains 74.6% of the variance, and correlates negatively with log frequency values in each language (N.B.: PC1 correlates positively with frequency in the original source from which the data are taken, but this is trivial). PC1 is positively correlated with basicness for all concepts in both data sets (Spearman's $\rho = 0.47$, $p < 0.001$).

Figure 15 displays correlations between median ratios (log-transformed to aid in visibility) between loss rates with and without IC and the two metrics described above (basicness: top row, PC1: bottom row). Higher log median ratio values indicate that words for a different concept are more likely to be replaced when they contain identical consonants as opposed to when they do not. Higher values for the two metrics described above mean that a concept is less basic/frequent. Negative correlations between log median rates and each metric indicate that the tendency to replace forms with identical consonants (relative to those without identical consonants) is weaker in less basic/frequent concepts than in more basic/frequent ones. The basicness metric correlates negatively with median log ratios in all five families under analysis, though not all correlations are significant under a significance level of $\alpha = 0.005$ ($= 0.05/10$ comparisons), adjusted for multiple testing via the Bonferroni correction. PC1 correlates negatively with median log ratios in three out of five families, but none of these correlations reach significance when accounting for multiple testing.

It is not immediately clear why basicness shows a strong correlation with log median ratio for the most part and PC1 (reflecting concept frequency) does not. One possibility is that despite being an aggregate measure, basicness is based on a broader, more phylogenetically diverse sample as opposed to the more skewed data set available for frequencies, and better captures aspects of need probability than the frequency sample. A more troubling possibility is that the simplicity metric involved in the basicness measure is related to word length, and fails to control for this particular confound.

From these analyses, the evidence that concepts with lower need probability are more likely to tolerate identical consonants (in terms of vocabulary replacement rates) are mixed. Part of the difficulty in providing convincing evidence for this hypothesis stems from the lack of appropriate resources capturing the dynamics of need probability on a broad cross-linguistic scale. An important point is the following: there is the greatest degree of inter-concept variation in terms of vocabulary replacement rates (i.e., loss rates for +IC vs. −IC). Therefore, it appears to be the case that vocabulary replacement rates are the component of change most capable of allowing +IC to be fostered in some cognate-concept traits but not others, and ultimately preventing cognate forms with IC from dying out more frequently than cognate forms without IC.

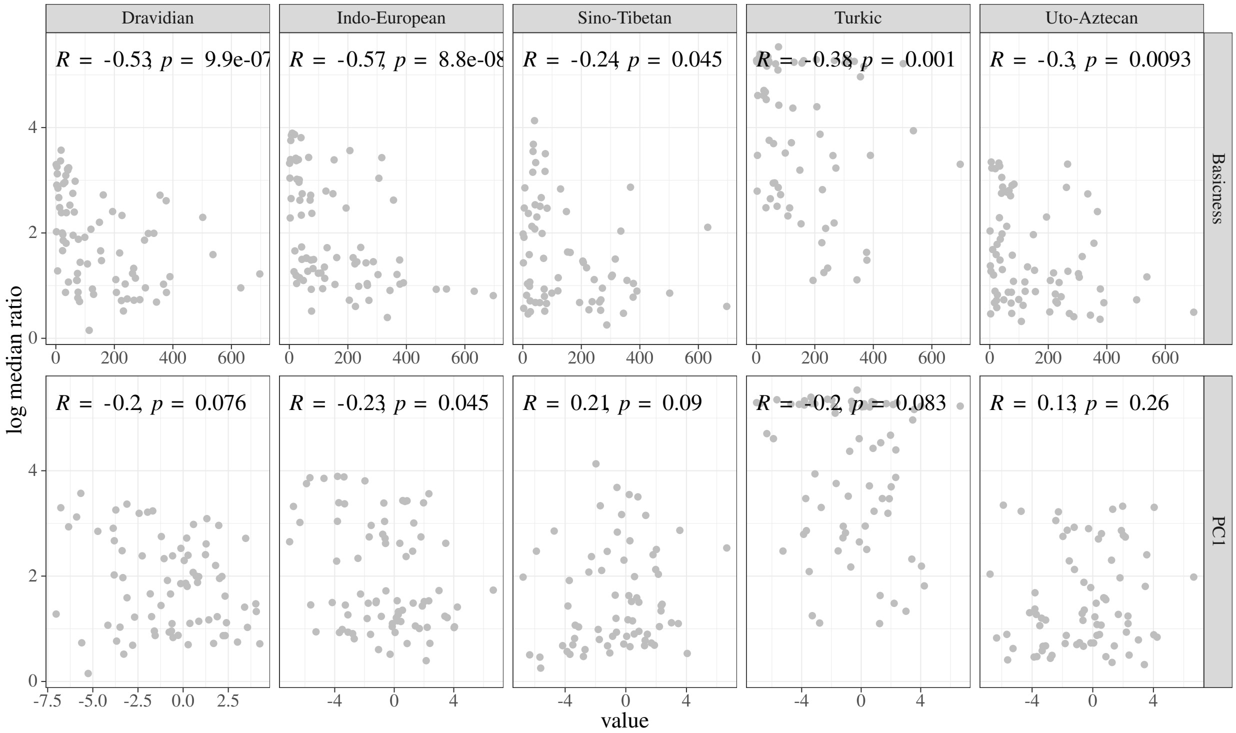

Figure SI 15: Correlations (Spearman's ρ) between median log ratios between cognate-concept loss rates with vs. without IC and basicness (top row) and the first principal component of cross-linguistic concept frequencies (bottom row), for all five families analyzed in this paper. Negative correlations between log median rates and each metric indicate that the tendency to replace forms with identical consonants (relative to those without identical consonants) is weaker in less basic/frequent concepts than in more basic/frequent ones. Correlations and significance values were generated and annotated using the R package `ggpubr` [70].

Appendix A HDIs of concept-level transition rate ratios

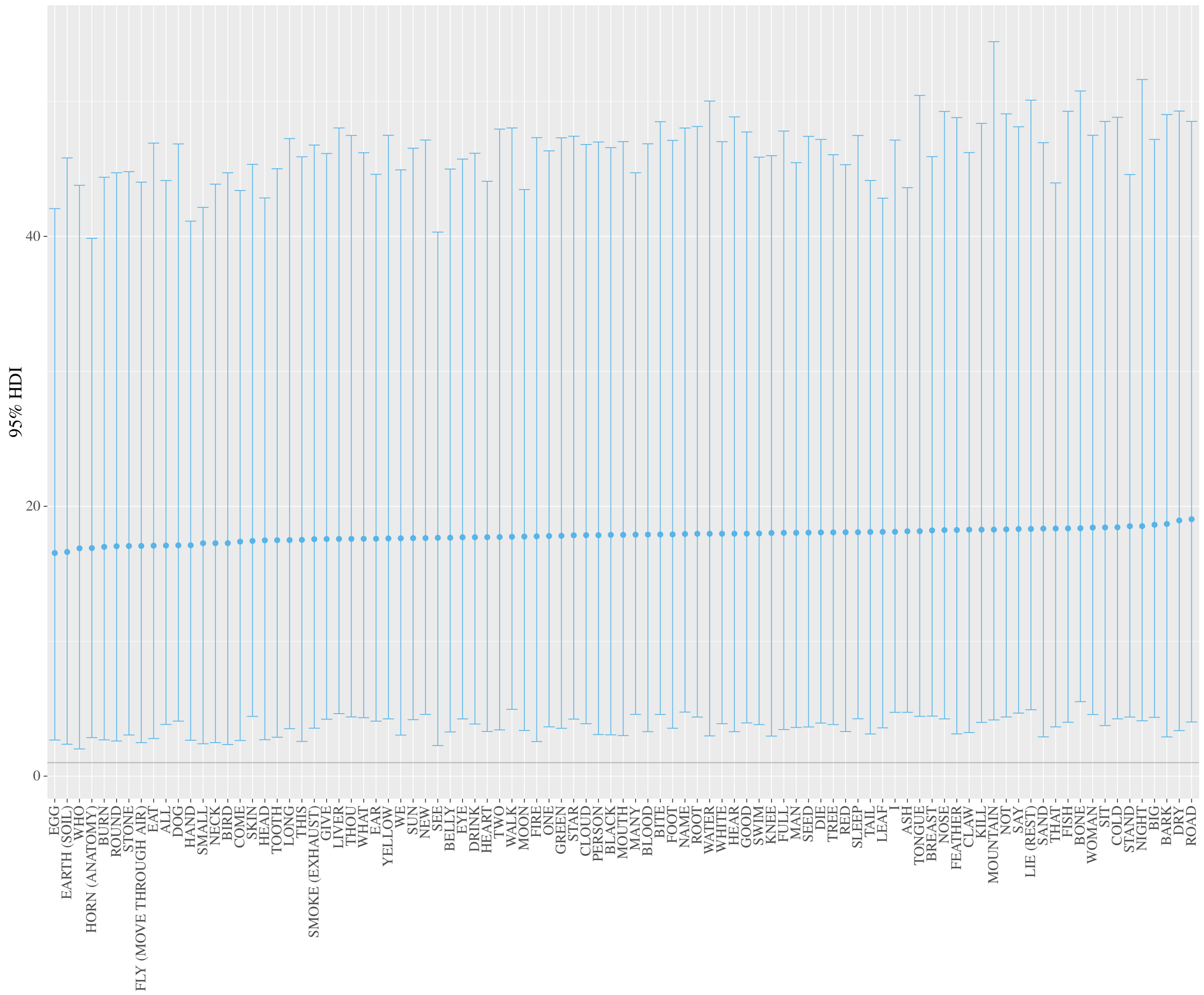

Figure SI 16: Concept-level ratios between birth rates without vs. with IC for cognate-concept traits in Dravidian

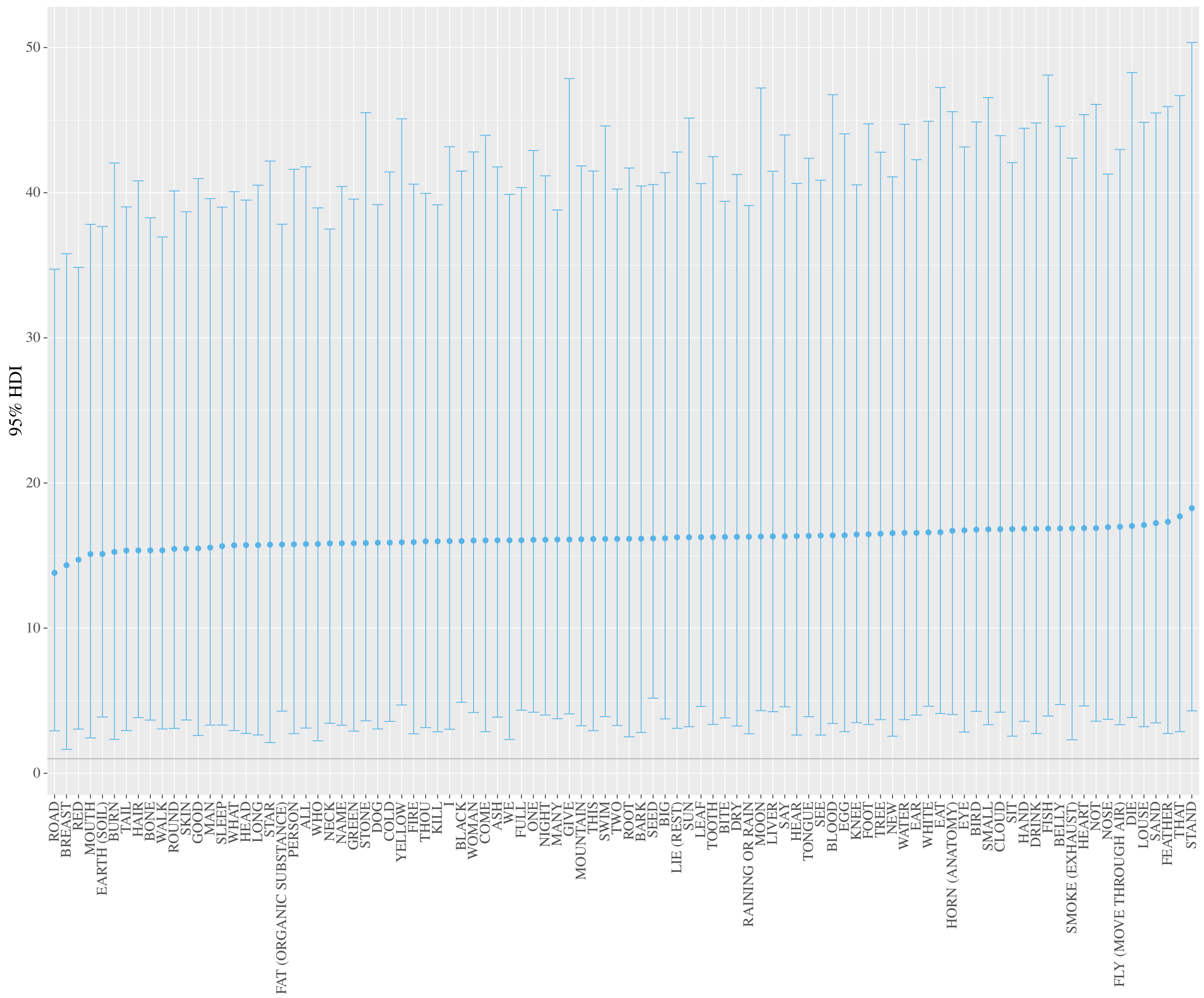

Figure SI 17: Concept-level ratios between birth rates without vs. with IC for cognate-concept traits in Indo-European

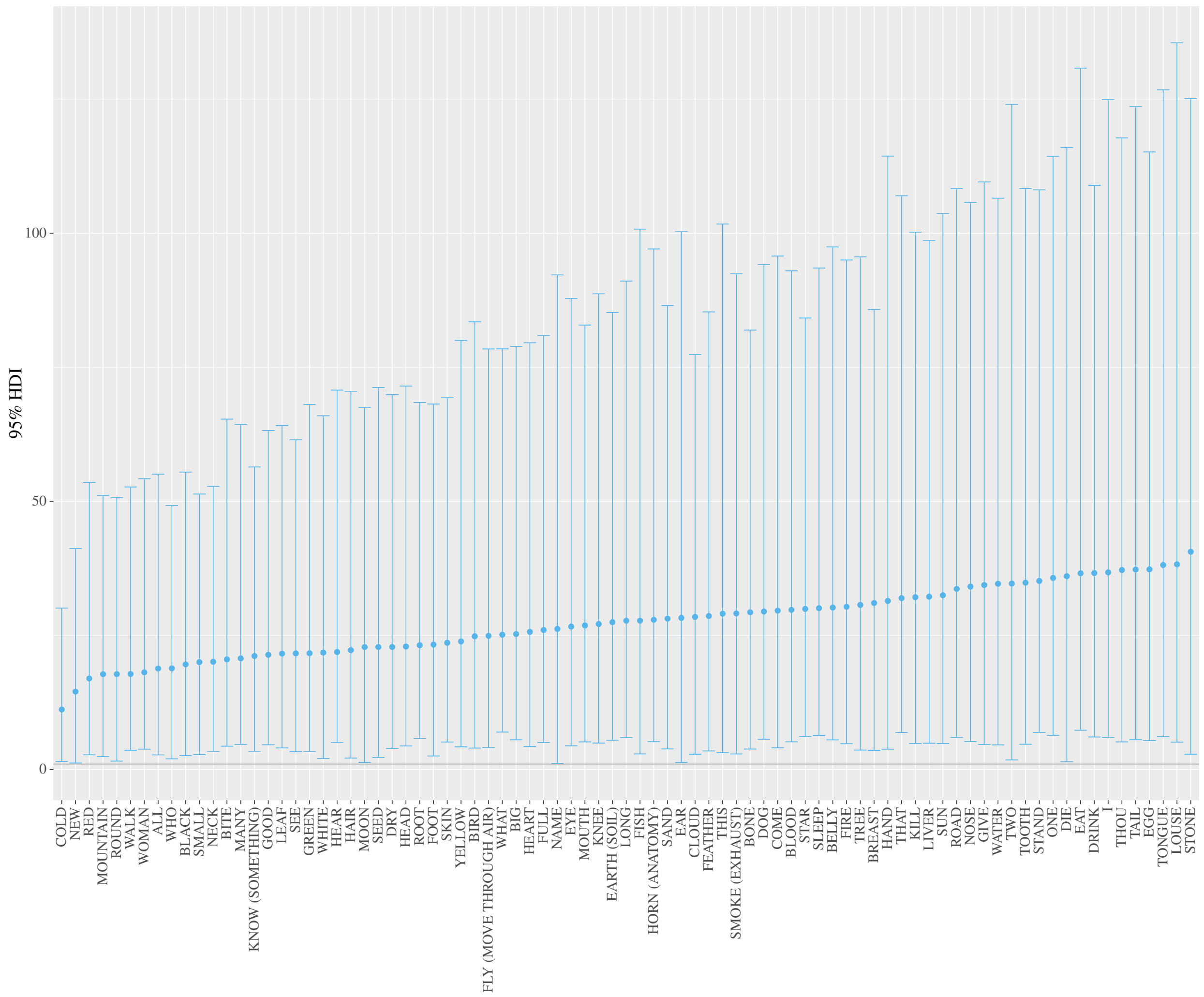

Figure SI 18: Concept-level ratios between birth rates without vs. with IC for cognate-concept traits in Sino-Tibetan

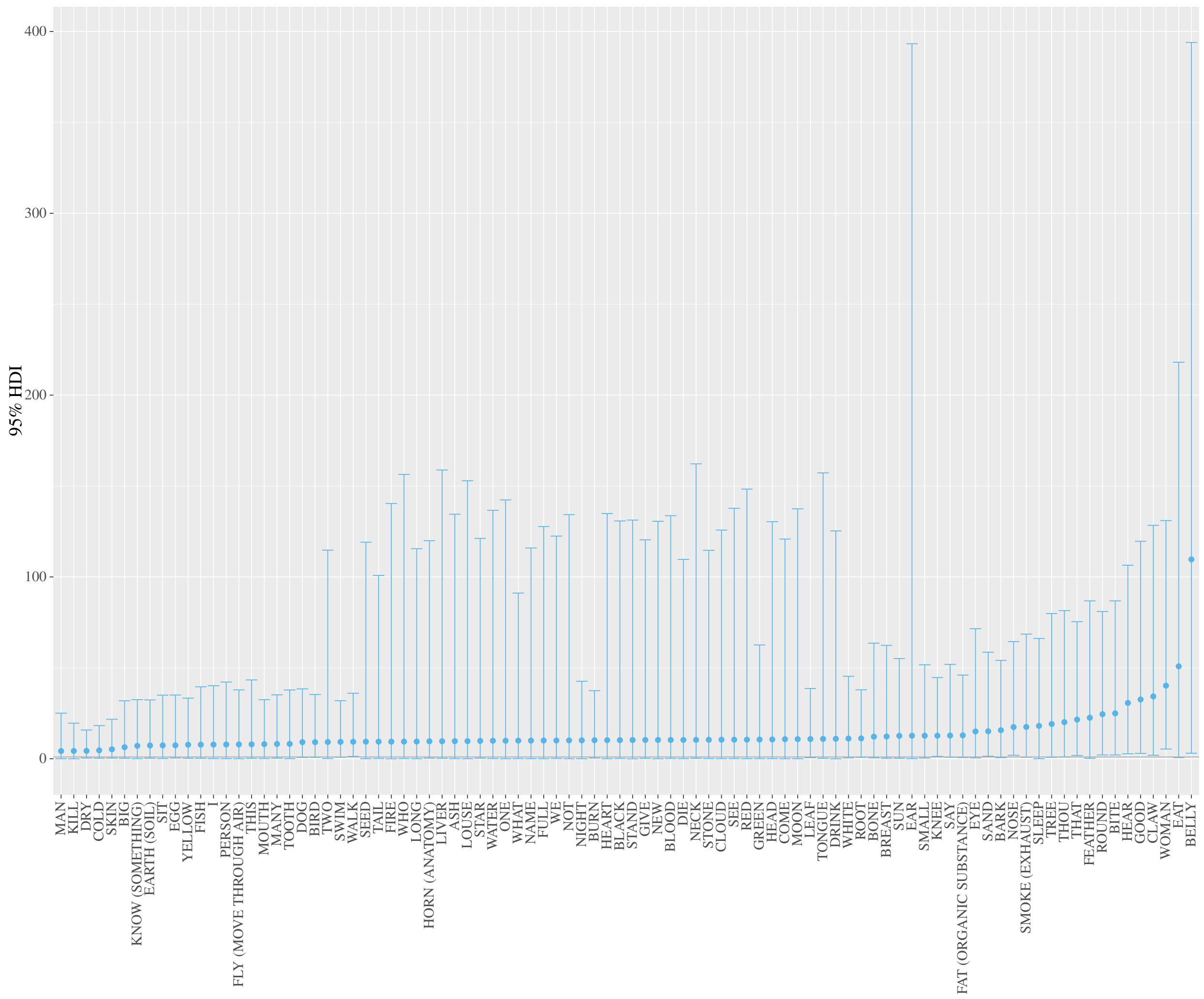

Figure SI 19: Concept-level ratios between birth rates without vs. with IC for cognate-concept traits in Turkic

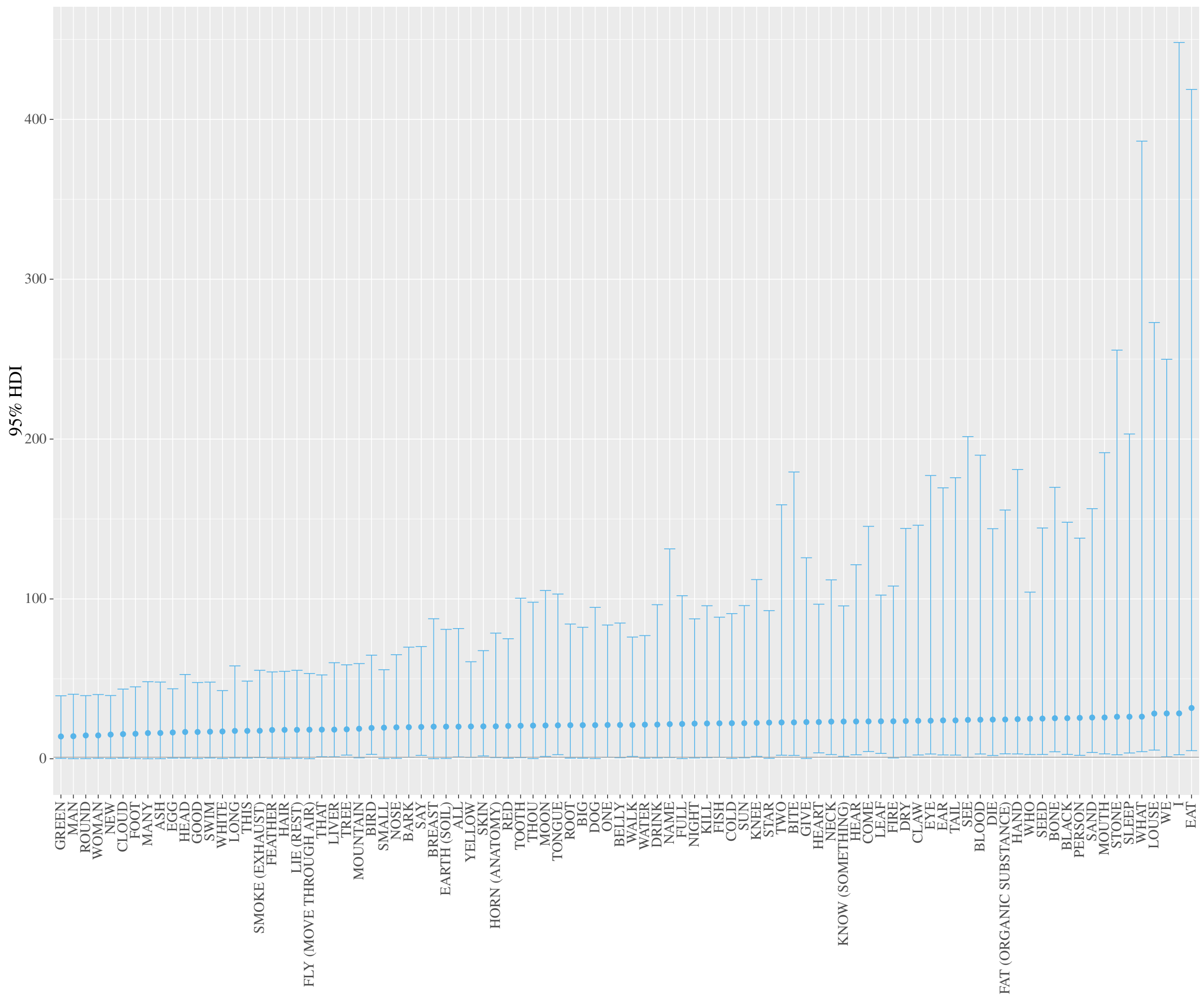

Figure SI 20: Concept-level ratios between birth rates without vs. with IC for cognate-concept traits in Uto-Aztecan

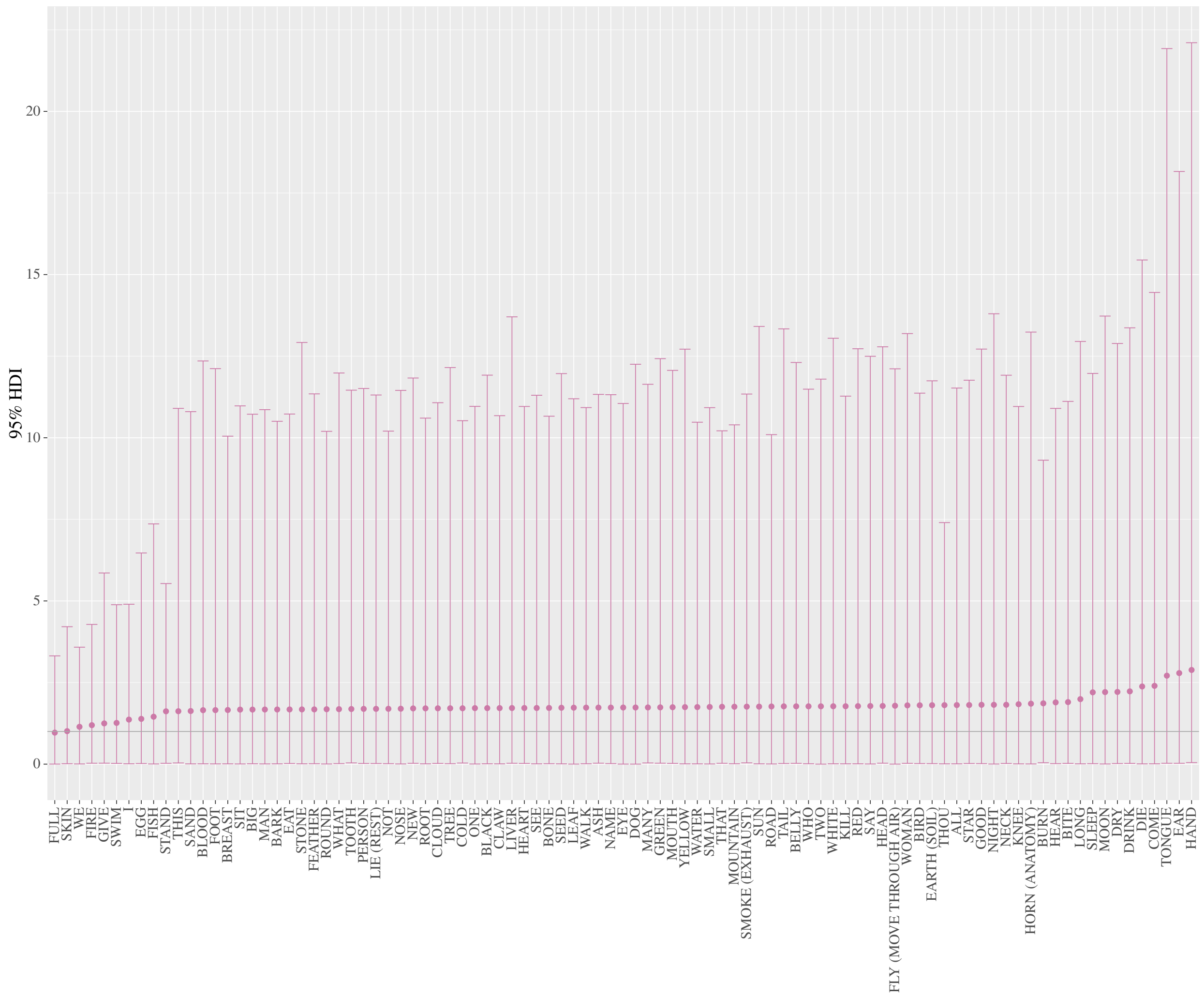

Figure SI 21: Concept-level ratios between mutation rates removing vs. introducing IC for cognate-concept traits in Dravidian

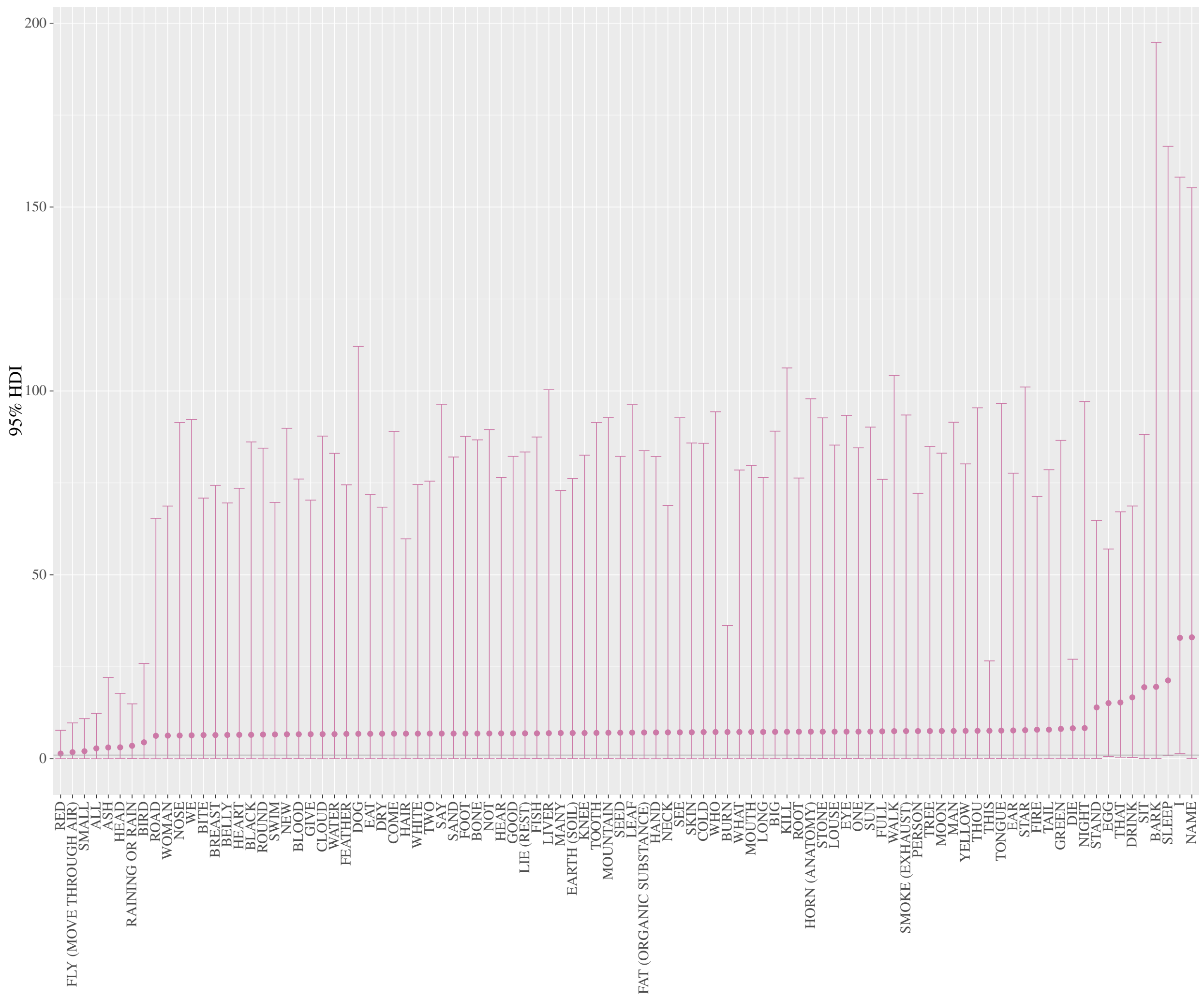

Figure SI 22: Concept-level ratios between mutation rates removing vs. introducing IC for cognate-concept traits in Indo-European

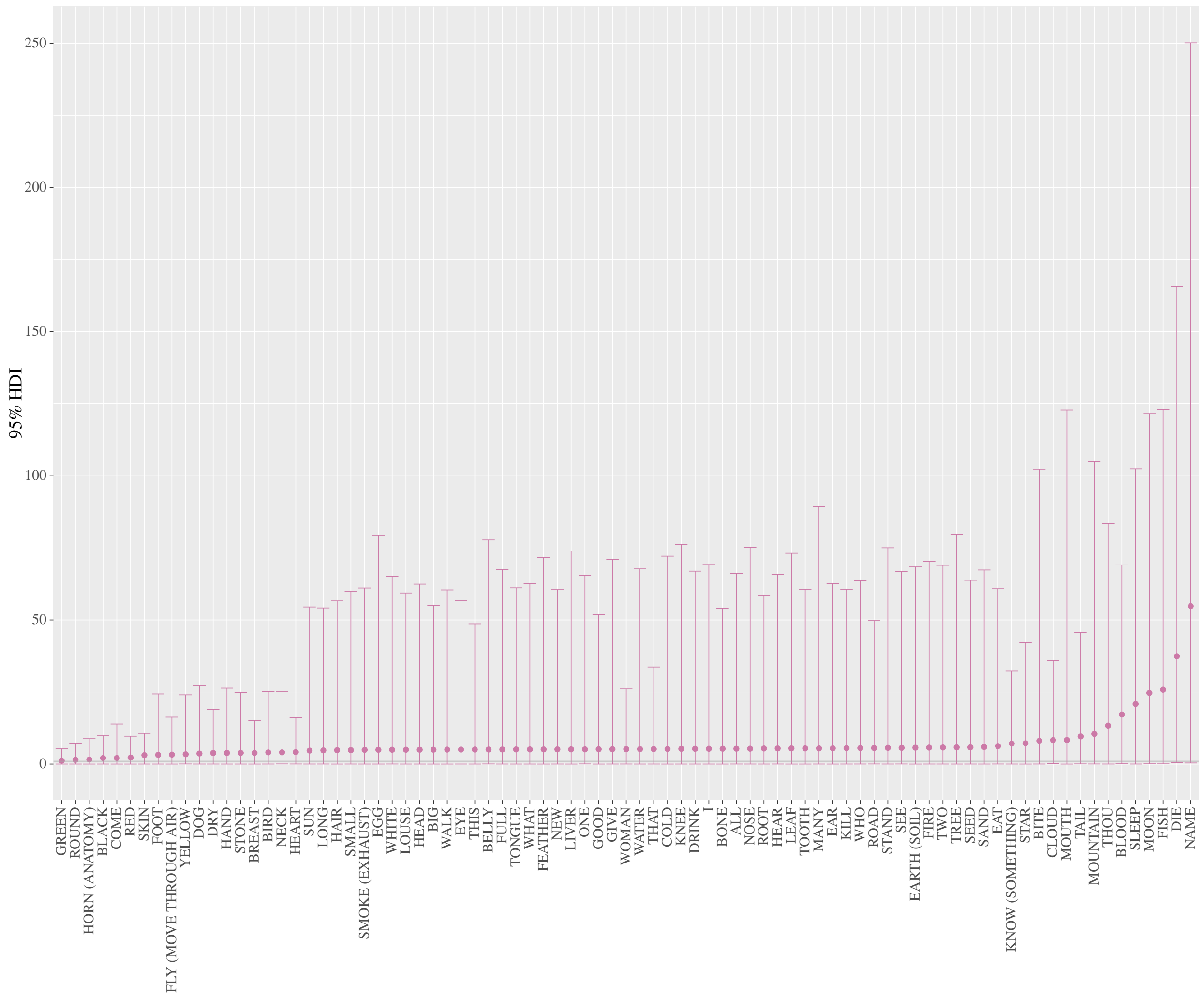

Figure SI 23: Concept-level ratios between mutation rates removing vs. introducing IC for cognate-concept traits in Sino-Tibetan

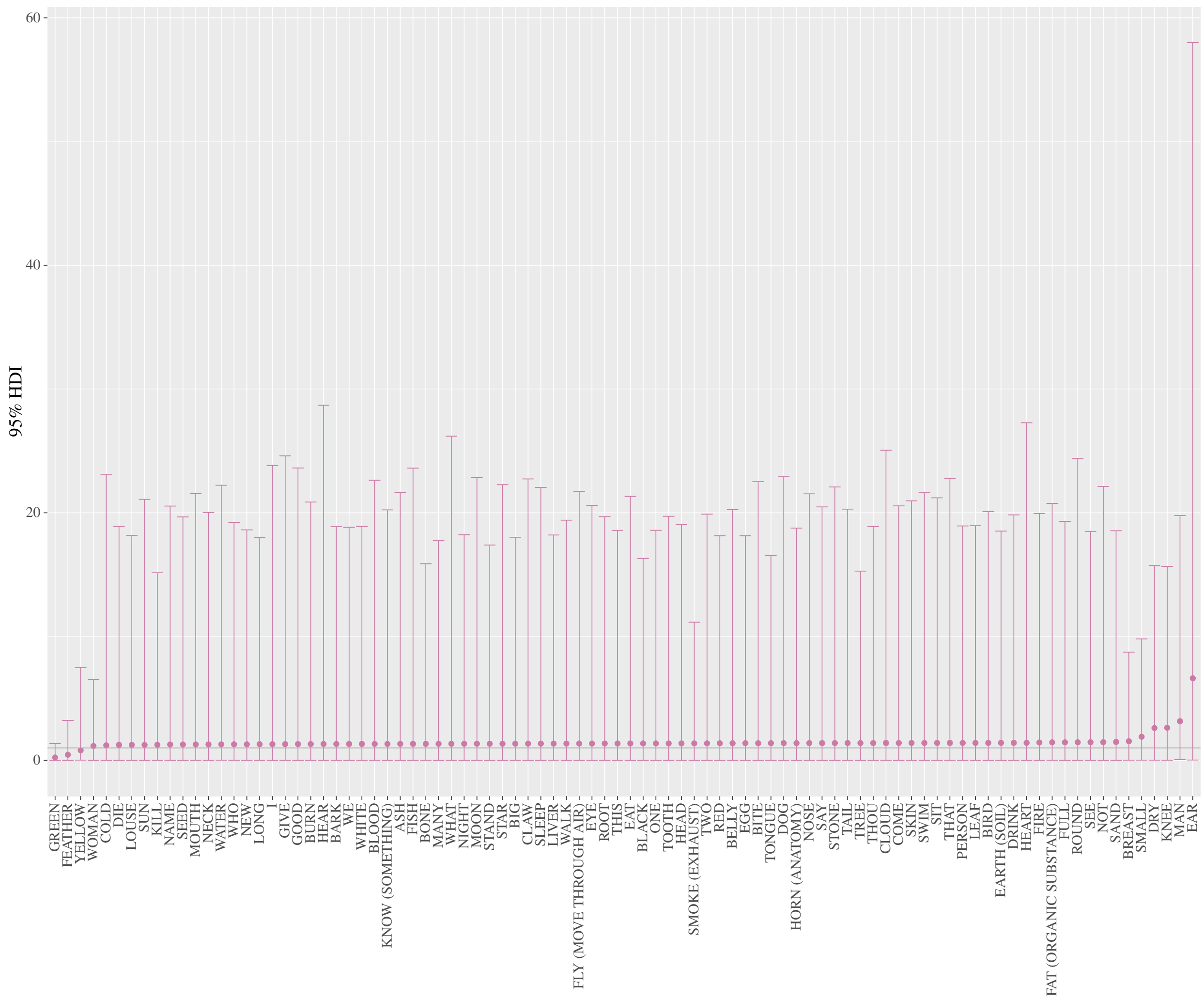

Figure SI 24: Concept-level ratios between mutation rates removing vs. introducing IC for cognate-concept traits in Turkic

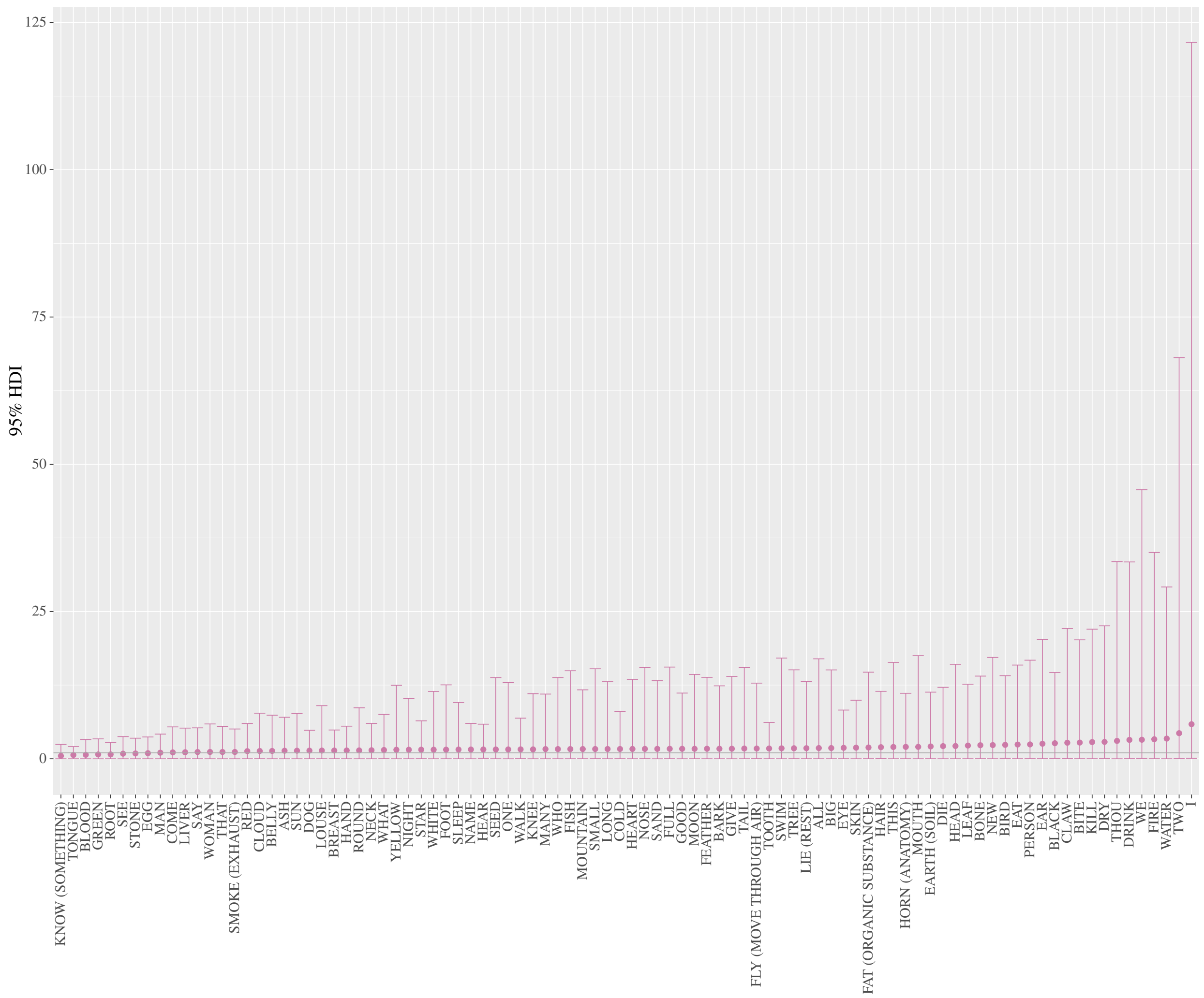

Figure SI 25: Concept-level ratios between mutation rates removing vs. introducing IC for cognate-concept traits in Uto-Aztecan

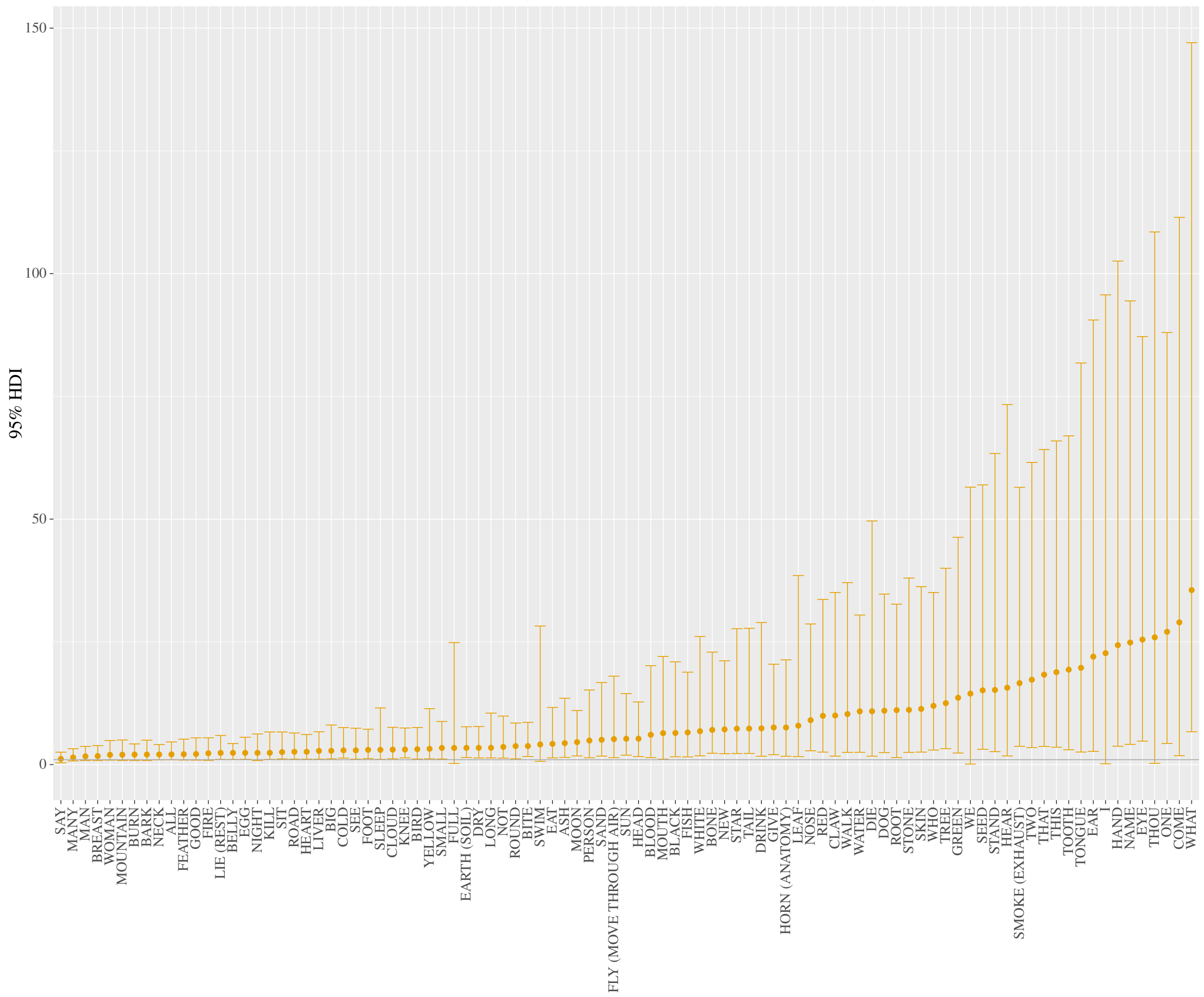

Figure SI 26: Concept-level ratios between loss rates with vs. without IC for cognate-concept traits in Dravidian

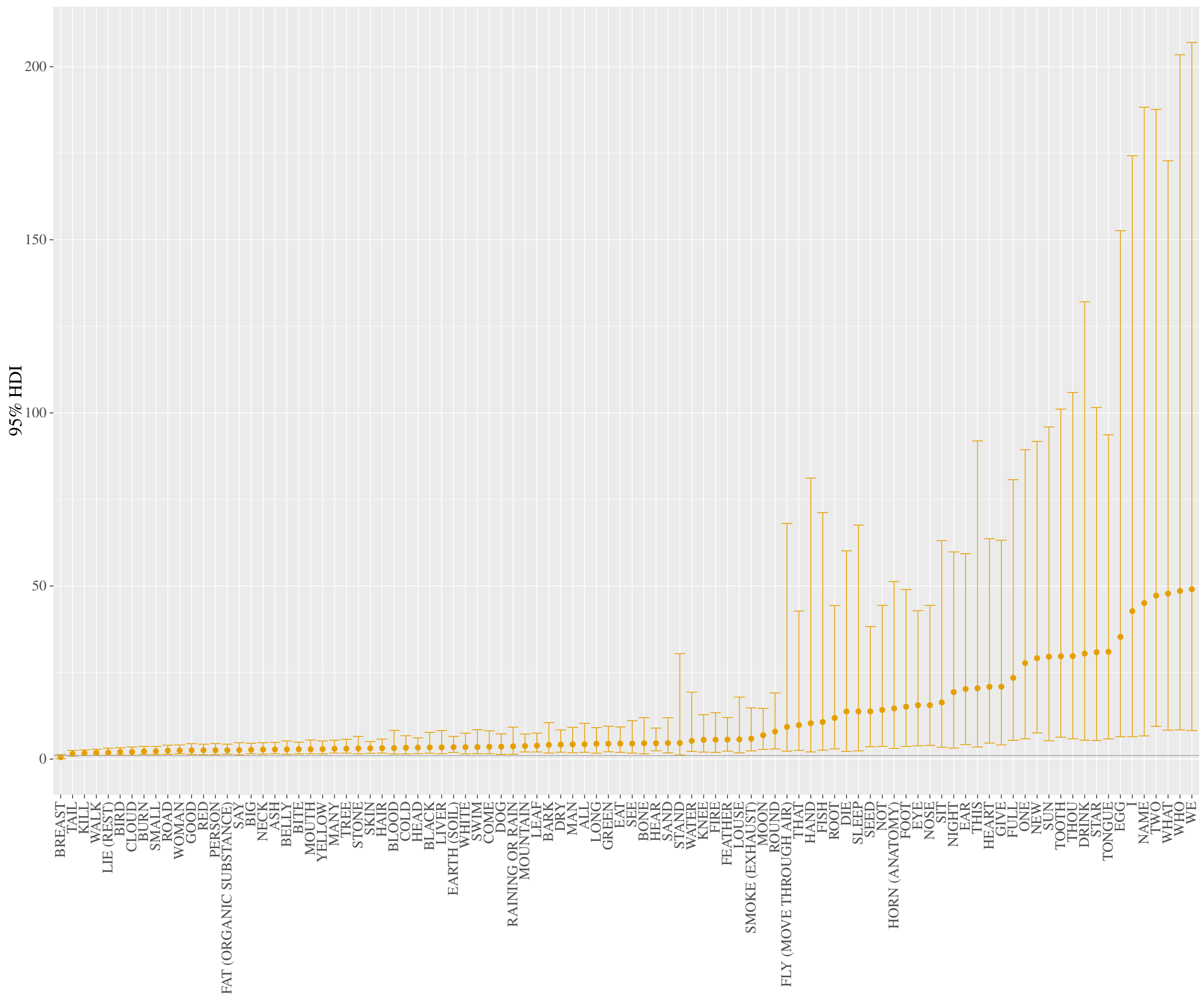

Figure SI 27: Concept-level ratios between loss rates with vs. without IC for cognate-concept traits in Indo-European

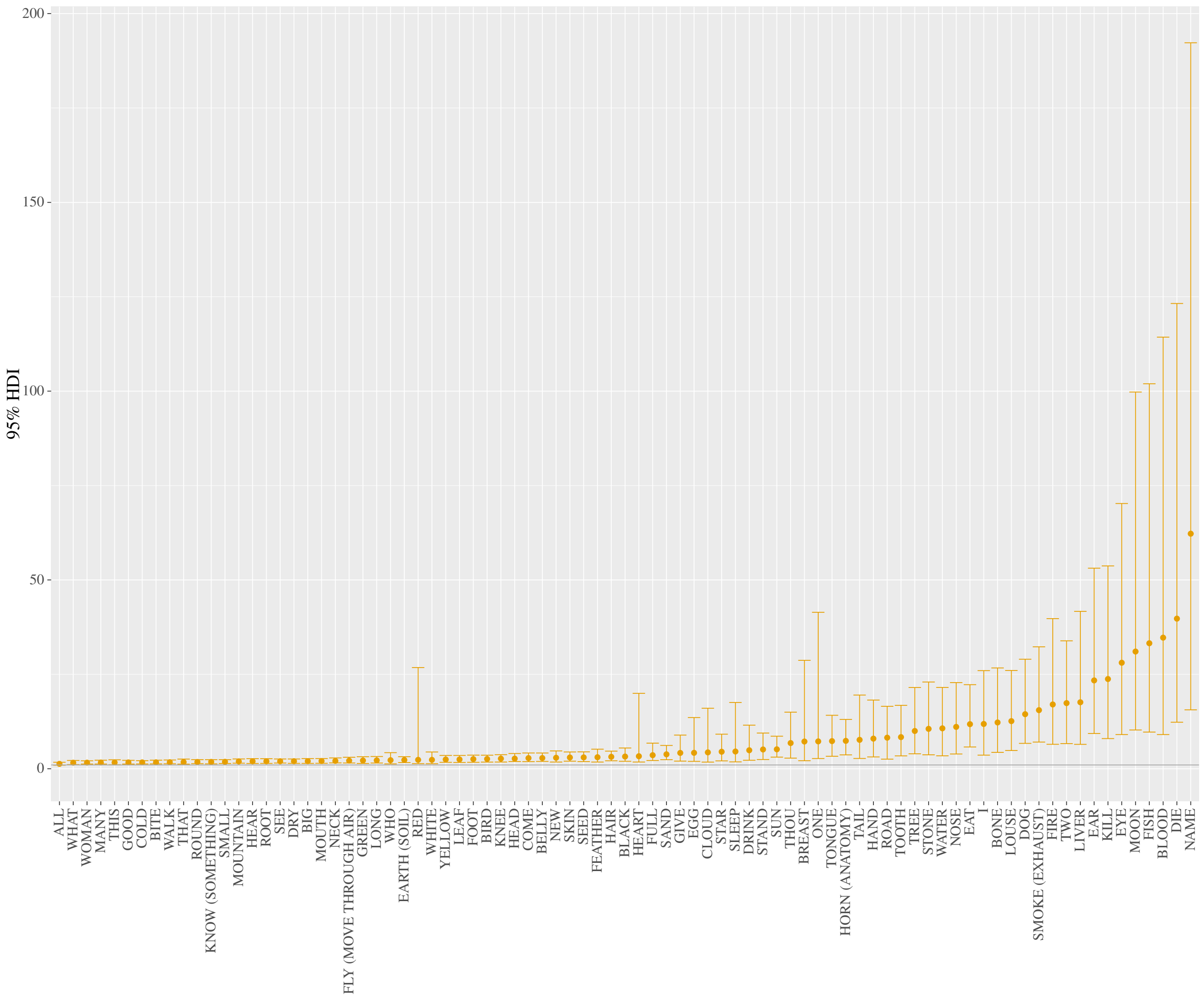

Figure SI 28: Concept-level ratios between loss rates with vs. without IC for cognate-concept traits in Sino-Tibetan

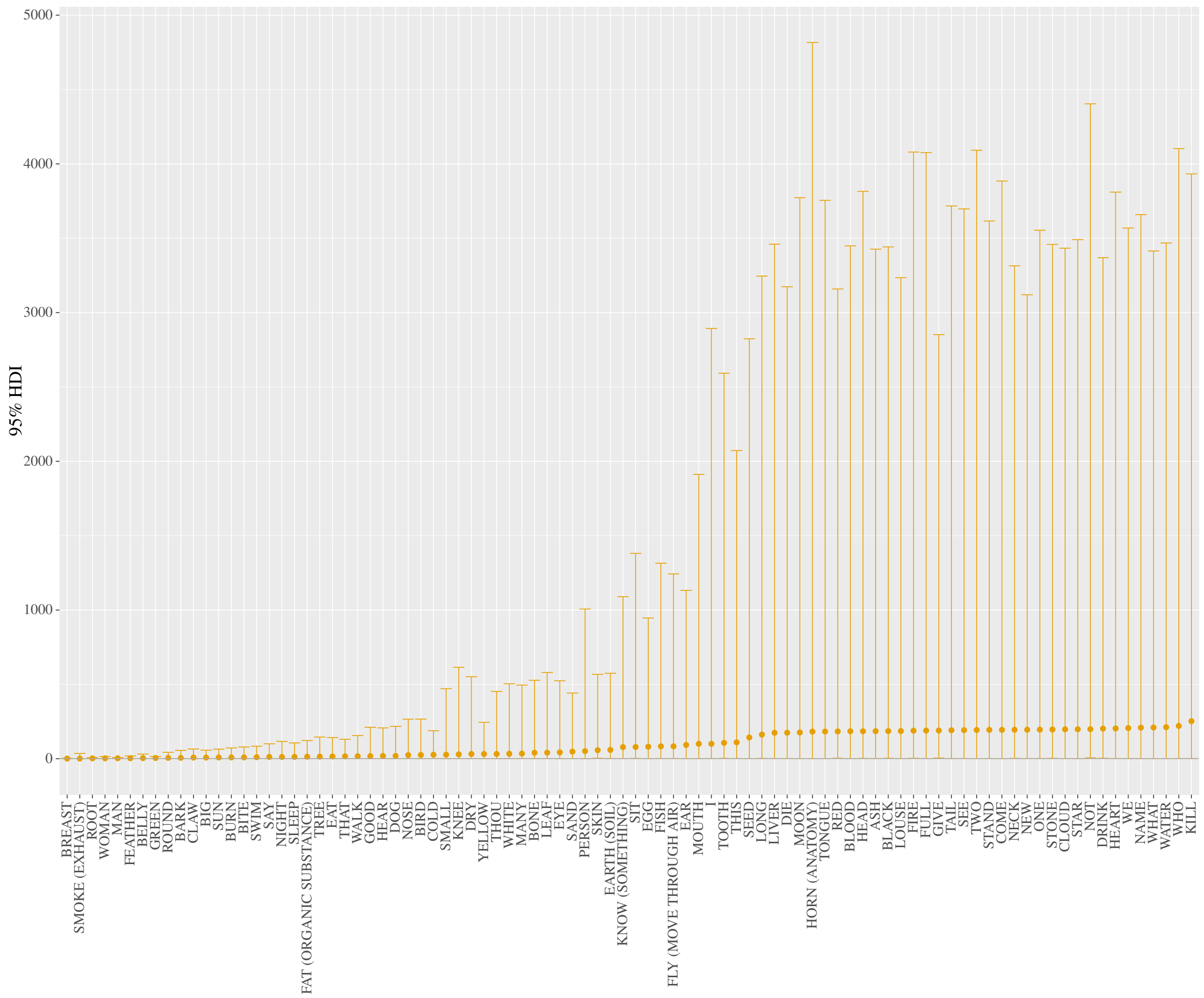

Figure SI 29: Concept-level ratios between loss rates with vs. without IC for cognate-concept traits in Turkic

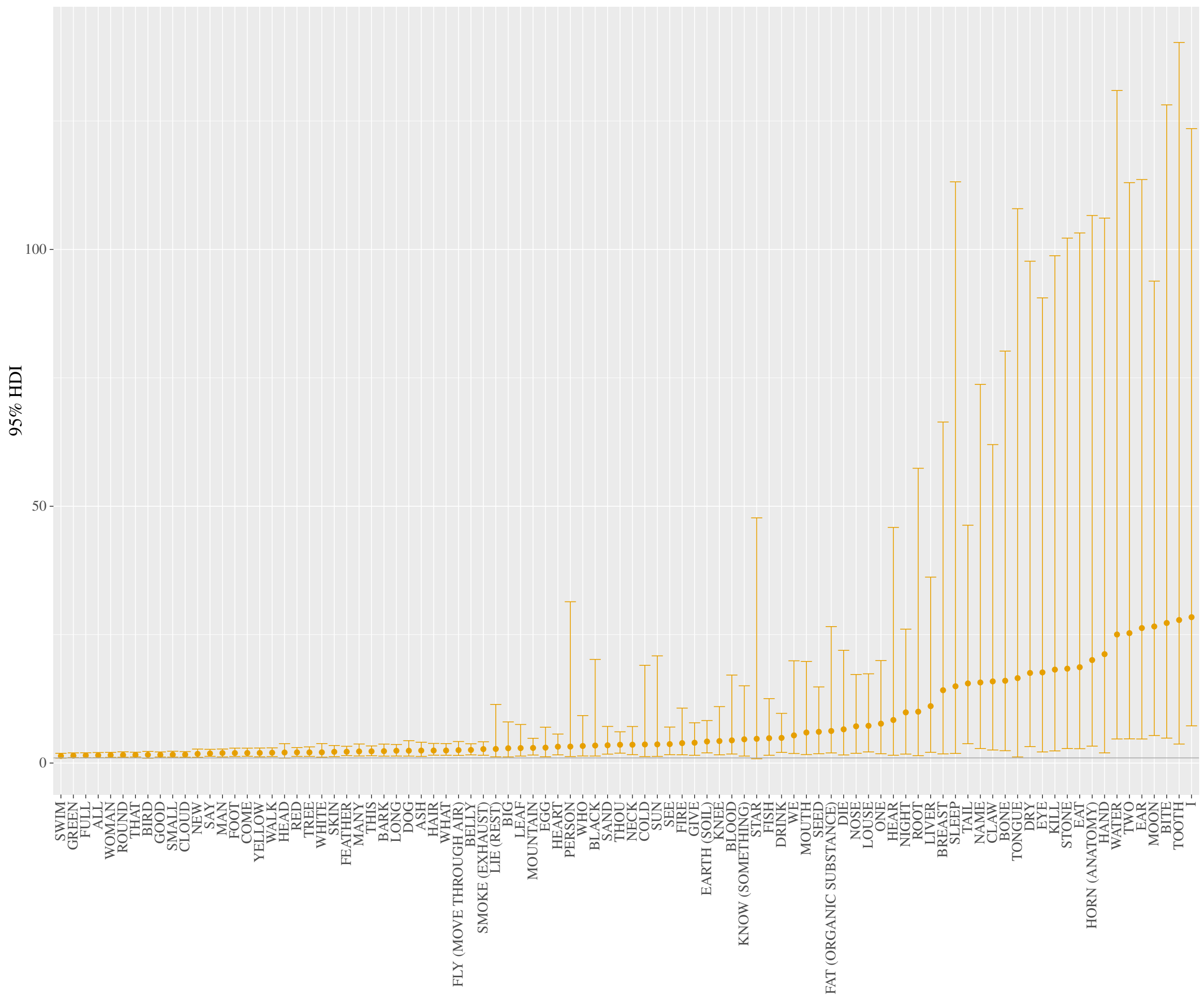

Figure SI 30: Concept-level ratios between loss rates with vs. without IC for cognate-concept traits in Uto-Aztecan

Appendix B Simulation-based validation

The analyses carried out in this paper assume that it is possible for a continuous-time Markov model of character evolution to accurately distinguish asymmetries between birth rates, mutation rates, and loss rates characterizing the evolution of a lexical trait. At the same time, given the fact that Bayesian phylogenetic models infer rates on the basis of character data and phylogenetic trees alone, it is possible that such models do not accurately recapitulate properties of the evolutionary process that are of interest to the research questions explored in this paper.

To investigate this issue, I simulated data sets consisting of 100 characters evolving over a

random ultrametric tree with 100 tips according to a shared CTM process. Under such a process, birth, mutation, and loss rates can be symmetric (i.e., share the same randomly generated transition rate) or asymmetric (i.e., sharing two independently and identically distributed transition rates), yielding eight possible settings of symmetry/asymmetry for birth, mutation and loss rates. I simulated random data and inferred the transition rates for each of these eight settings over 100 iterations. This yielded $8 \times 100 \times 3 = 2400$ simulated rate comparisons. The simulation and inference setups were restricted to homoplastic characters (as in this paper's analyses of cognate-concept traits) simpler than the hierarchical models used in this paper, since it is computationally unfeasible to investigate the behavior of similarly complex models for several hundred simulated data sets. Nonetheless, these studies provide a sense of whether models of this sort capture the properties of evolution that they are intended to capture.

For comparison, posterior rates were converted into ratios in the manner described in this paper's analysis of results. The 95% HDI of each inferred ratio's posterior distribution was computed, along with the ratio of the true rates used to simulate the data. A comparison was coded as a true negative if the true ratio was equal to 1 and the HDI contained 1. A comparison was coded as a false negative if the true ratio was not equal to 1 but the HDI contained 1. A comparison was coded as a true positive if the true ratio was not equal to 1 and the HDI did not contain 1 (but was on the same side of 1 as the true ratio value); a comparison was coded as a sign error if the true ratio was not equal to 1 and the HDI did not contain 1 (but was *not* on the same side of 1 as the true ratio value). Finally, a comparison was coded as a false positive if the true ratio was equal to 1 but the HDI did not contain 1. Of these attempts to recapitulate parameters on the basis of which data were simulated, 44.6% consisted of true negatives, 41.7% consisted of true positives, 8.14% consisted of false negatives, 5.1% consisted of false positives, and 0.29% consisted of sign errors. This corresponds to a precision value of 0.89 and recall value of 0.83, and an F1 score of 0.86. Of the true positive results — results where the HDI was on the same side of 1 as the true ratio value — the HDI excluded the true value in 24.8% of instances. Of these instances, the inference procedure infers a more extreme posterior distribution over ratios (i.e., further away from 1) in only 8.9%. In sum, the greater tendency on the part of inferred rates toward false negatives (versus false positives) and a tendency to underestimate rather than overestimate values speaks to a degree of conservativeness on the part of the methodology used in this paper, but not perhaps an excessive one. Since some of the results reported in this paper are negative ones, this simulation-based finding may be a mild cause for concern, but it is worth also recalling that the data sets employed are quite large — if false negatives and conservative estimates are an artifact of smaller sample sizes and low statistical power, this may not be a major concern here. Furthermore, this paper's models

display remarkable consistency in their results. Code used to run simulations can be found at `https://github.com/chundrac/idcc/tree/main/simulations/simulation.R`.

References

[1] Robert Blust and Stephen Trussel. The Austronesian comparative dictionary: a work in progress. *Oceanic Linguistics*, 52(2):493–523, 2013.

[2] Johann-Mattis List, Robert Forkel, Simon J Greenhill, Christoph Rzymski, Johannes Englisch, and Russell D Gray. Lexibank, a public repository of standardized wordlists with computed phonological and lexical features. *Scientific Data*, 9(1):1–16, 2022.

[3] Leonid Kogan and Ilya Arkhipov. Semitic etymological database online (`http://sed-online.ru`).

[4] István Bátori. Uralische Etymologische Datenbasis `http://uralothek.uni-koblenz.de:8080/Uralothek/pdom/basis.html`, 2010.

[5] Uralonet `http://uralonet.nytud.hu/`.

[6] Károly Rédei. *Uralisches Etymologisches Wörterbuch*. Akadémiai Kiadó, Budapest, 1986-1991.

[7] Ante Aikio. Uralic etymological dictionary (draft version of entries a–ć). *URL: https://www. academia. edu/41659514/URALIC_ETYMOLOGICAL_DICTIONARY_draft_version_of_entries_A_Ć_*, 2020.

[8] Joseph H Greenberg. The patterning of root morphemes in semitic. *Word*, 6(2):162–181, 1950.

[9] Gustav Herdan. The patterning of semitic verbal roots subjected to combinatory analysis. *Word*, 18(1-3):262–268, 1962.

[10] Zygmunt Frajzyngier. Notes on the r1r2r3. stems in semitic. *Journal of semitic studies*, 24(1):1–12, 1979.

[11] Eulàlia Vernet. Semitic root incompatibilities and historical linguistics. *Journal of Semitic studies*, 56(1):1–18, 2011.

[12] Péter Rácz, Jennifer Hay, Jeremy Needle, Jeanette King, and Janet B Pierrehumbert. Gradient māori phonotactics. *Te Reo*, 59, 2016.

[13] Kie Zuraw and Yu-An Lu. Diverse repairs for multiple labial consonants. *Natural Language & Linguistic Theory*, 27(1):197–224, 2009.

[14] R. David Zorc. A study of the Aklanon dialect, volume two: Dictionary (of root words and derivations), Aklanon to English. 1969.

[15] Saul B. Needleman and Christian D. Wunsch. A general method applicable to the search for similarities in the amino acid sequence of two proteins. *Journal of Molecular Biology*, 48:443–53, 1970.

[16] Gerhard Jäger. Phylogenetic inference from word lists using weighted alignment with empirical determined weights. *Language Dynamics and Change*, 3:245–291, 2013.

[17] Joseph Felsenstein. *Inferring phylogenies*. Sinauer Associates, Sunderland, Mass., 2004.

[18] Russell D Gray, Alexei J Drummond, and Simon J Greenhill. Language phylogenies reveal expansion pulses and pauses in Pacific settlement. *Science*, 323(5913):479–483, 2009.

[19] Andrew Kitchen, Christopher Ehret, Shiferaw Assefa, and Connie J Mulligan. Bayesian phylogenetic analysis of semitic languages identifies an early bronze age origin of semitic in the near east. *Proceedings of the Royal Society B: Biological Sciences*, 276(1668):2703–2710, 2009.

[20] T. Honkola, O. Vesakoski, K. Korhonen, J. Lehtinen, K. Syrjänen, and N. Wahlberg. Cultural and climatic changes shape the evolutionary history of the uralic languages. *Journal of Evolutionary Biology*, 26(6):1244–1253, 2013.

[21] Harald Hammarström, Robert Forkel, and Martin Haspelmath. Glottolog 3.3. Max Planck Institute for the Science of Human History, 2017.

[22] Wayne P. Maddison. Missing data versus missing characters in phylogenetic analysis. *Systematic Biology*, 42(4):576–581, 1993.

[23] Sergei Tarasov. Integration of anatomy ontologies and evo-devo using structured markov models suggests a new framework for modeling discrete phenotypic traits. *Systematic biology*, 68(5):698–716, 2019.

[24] Geoff K Nicholls and Russell D Gray. Quantifying uncertainty in a stochastic model of vocabulary evolution. *Phylogenetic methods and the prehistory of languages*, pages 161–171, 2006.

[25] Alexander V. Alekseyenko, Christopher J. Lee, and Marc A. Suchard. Wagner and Dollo: A Stochastic Duet by Composing Two Parsimonious Solos. *Systematic Biology*, 57(5):772–784, 10 2008.

[26] Joseph Felsenstein. Evolutionary trees from DNA sequences: A maximum likelihood approach. *Journal of Molecular Evolution*, 17(6):367–76, 1981.

[27] Remco R Bouckaert and Martine Robbeets. Pseudo Dollo models for the evolution of binary characters along a tree. *BioRxiv*, 2017.

[28] Joseph Felsenstein. Phylogenies from restriction sites: a maximum-likelihood approach. *Evolution*, 46(1):159–173, 1992.

[29] R. Bouckaert, P. Lemey, M. Dunn, S. J. Greenhill, A. V. Alekseyenko, A. J. Drummond, R. D. Gray, M. A. Suchard, and Q. D. Atkinson. Mapping the origins and expansion of the Indo-European language family. *Science*, 337(6097):957–960, 2012.

[30] William Chang, Chundra Cathcart, David Hall, and Andrew Garrett. Ancestry-constrained phylogenetic analysis supports the Indo-European Steppe Hypothesis. *Language*, 91(1):194–244, 2015.

[31] Steven Moran and Daniel McCloy, editors. *PHOIBLE 2.0*. Max Planck Institute for the Science of Human History, Jena, 2019.

[32] Martin Kümmel. *Konsonantenwandel*. Dr. Ludwig Reichert Verlag, Wiesbaden, 2007.

[33] Donald Ringe, Tandy Warnow, and Ann Taylor. Indo-European and computational cladistics. *Transactions of the Philological Society*, 100(1):59–129, 2002.

[34] Luay Nakhleh, Donald Ringe, and Tandy Warnow. Perfect phylogenetic networks: A new methodology for reconstructing the evolutionary history of natural languages. *Language*, 81(2):382–420, 2005.

[35] Johann-Mattis List, Robert Forkel, Simon J. Greenhill, Christoph Rzymski, Johannes Englisch, and Russell D. Gray. Lexibank: A publicly available repository of standardized lexical datasets

with automatically computed phonological and lexical features for more than 2000 language varieties, August 2021.

[36] Johann Mattis List, Annika Tjuka, Christoph Rzymski, Simon Greenhill, and Robert Forkel, editors. *CLLD Concepticon 3.0.0*. Max Planck Institute for Evolutionary Anthropology, Leipzig, 2022.

[37] Vishnupriya Kolipakam, Michael Dunn, Fiona M Jordan, and Annemarie Verkerk. Dravlex: A dravidian lexical database:(version v1. 0.0)[data set]. 2018.

[38] Vishnupriya Kolipakam. CLDF dataset derived from Kolipakam et al.'s "DravLex:" from 2018, July 2021.

[39] Vishnupriya Kolipakam, Fiona M Jordan, Michael Dunn, Simon J Greenhill, Remco Bouckaert, Russell D Gray, and Annemarie Verkerk. A bayesian phylogenetic study of the dravidian language family. *Royal Society open science*, 5(3):171504, 2018.

[40] Michael Dunn. Indo-european lexical cognacy database (ielex). *Nijmegen: Max Planck Institute for Psycholinguistics*, 2012.

[41] Michael Dunn. Cldf dataset derived from dunn's "ielex" from 2012, July 2021.

[42] Laurent Sagart, Guillaume Jacques, Yunfan Lai, and Johann-Mattis List. Sino-tibetan database of lexical cognates, 2019.

[43] Laurent Sagart, Guillaume Jacques, Yunfan Lai, Robin J Ryder, Valentin Thouzeau, Simon J Greenhill, and Johann-Mattis List. Dated language phylogenies shed light on the ancestry of sino-tibetan. *Proceedings of the National Academy of Sciences*, 116(21):10317–10322, 2019.

[44] Alexander Savelyev and Martine Robbeets. Bayesian phylolinguistics infers the internal structure and the time-depth of the turkic language family. *Journal of Language Evolution*, 5(1):39–53, 2020.

[45] Alexander Savelyev. CLDF dataset derived from Savelyev and Robbeet's "Internal Structure of the Turkic Language Family" from 2020, July 2021.

[46] Simon J Greenhill, Hannah J Haynie, Robert M Ross, Angela M Chira, Johann-Mattis List, Lyle Campbell, Carlos A Botero, and Russell D Gray. A recent northern origin for the uto-aztecan family. *Language*, 2023.

[47] Cormac Anderson, Tiago Tresoldi, Thiago Chacon, Anne-Maria Fehn, Mary Walworth, Robert Forkel, and Johann-Mattis List. A cross-linguistic database of phonetic transcription systems. In *Yearbook of the Poznan Linguistic Meeting*, volume 4, pages 21–53, 2018.

[48] Morris Swadesh. Towards greater accuracy in lexicostatistic dating. *International Journal of American Linguistics*, 21:121–137, 1955.

[49] Johannes Dellert, Thora Daneyko, Alla Münch, Alina Ladygina, Armin Buch, Natalie Clarius, Ilja Grigorjew, Mohamed Balabel, Hizniye Isabella Boga, Zalina Baysarova, et al. NorthEuraLex: a wide-coverage lexical database of Northern Eurasia. *Language resources and evaluation*, 54(1):273–301, 2020.

[50] James A Matisoff. Sino-tibetan etymological dictionary and thesaurus (stedt). *Berkeley: Sino-Tibetan Etymological Dictionary and Thesaurus Project. stedt. berkeley. edu/dissemination/STEDT. pdf (14 September, 2018)*, 2015.

[51] Mary Ritchie Key and Field work: 1948-1955. Nahuatl (sierra de zacapoaxtla variety). In Mary Ritchie Key and Bernard Comrie, editors, *The Intercontinental Dictionary Series*. Max Planck Institute for Evolutionary Anthropology, Leipzig, 2023.

[52] Zarina Estrada Fernández. Yaqui. In Martin Haspelmath and Uri Tadmor, editors, *World Loanword Database*. Max Planck Institute for Evolutionary Anthropology, Leipzig, 2009.

[53] Northern Paiute Language Project. Northern paiute language project (dictionary), 2005-2023.

[54] Liam J. Revell. phytools: An r package for phylogenetic comparative biology (and other things). *Methods in Ecology and Evolution*, 3:217–223, 2012.

[55] Bob Carpenter, Andrew Gelman, Matthew D. Hoffman, Daniel Lee, Ben Goodrich, Michael Betancourt, Marcus Brubaker, Jiqiang Guo, Peter Li, and Allen Riddell. Stan: A probabilistic programming language. *Journal of statistical software*, 76(1)(1):1–32, 2017.

[56] Andrew Gelman and Donald B. Rubin. Inference from iterative simulation using multiple sequences. *Statistical Science*, 7(4):457–472, 1992.

[57] Mike Meredith and John Kruschke. *HDInterval: Highest (Posterior) Density Intervals*, 2022. R package version 0.2.4.

[58] John K Kruschke. Bayesian analysis reporting guidelines. *Nature Human Behaviour*, 5(10):1282–1291, 2021.

[59] Fermin Moscoso del Prado. The missing baselines in arguments for the optimal efficiency of languages. In *Proceedings of the Annual Meeting of the Cognitive Science Society*, volume 35, 2013.

[60] Roger Lass. How real(ist) are reconstructions. In Charles Jones, editor, *Historical linguistics: Problems and perspectives*, pages 156–189. Routledge, London and New York, 1993.

[61] Gašper Beguš. Estimating historical probabilities of natural and unnatural processes. *Phonology*, 37(4):515–549, 2020.

[62] Andrea Ceolin. *Neutral Models of Sound Change*. PhD thesis, University of Pennsylvania, 2020.

[63] Andrew Gelman, Jennifer Hill, and Masanao Yajima. Why we (usually) don't have to worry about multiple comparisons. *Journal of research on educational effectiveness*, 5(2):189–211, 2012.

[64] Yang Xu, Emmy Liu, and Terry Regier. Numeral systems across languages support efficient communication: From approximate numerosity to recursion. *Open Mind*, 4:57–70, 2020.

[65] Johannes Dellert and Armin Buch. A new approach to concept basicness and stability as a window to the robustness of concept list rankings. *Language Dynamics and Change*, 8(2):157–181, 2018.

[66] Mark Pagel, Quentin D. Atkinson, and Andrew Meade. Frequency of word-use predicts rates of lexical evolution throughout Indo-European history. *Nature*, 449:717–720, 2007.

[67] George Kingsley Zipf. *The psycho-biology of language*. Houghton Mifflin Company, 1935.

[68] George Kingsley Zipf. *Human behavior and the principle of least effort*. Addison-Wesley Press, Cambridge, MA, 1949.

[69] Andreea S Calude and Mark Pagel. How do we use language? shared patterns in the frequency of word use across 17 world languages. *Philosophical Transactions of the Royal Society B: Biological Sciences*, 366(1567):1101–1107, 2011.

[70] Alboukadel Kassambara. *ggpubr: 'ggplot2' Based Publication Ready Plots*, 2023. R package version 0.6.0.
